# A hybrid pipeline for reconstruction and analysis of viral genomes at multi-organ level

**DOI:** 10.1093/gigascience/giaa086

**Published:** 2020-08-20

**Authors:** Diogo Pratas, Mari Toppinen, Lari Pyöriä, Klaus Hedman, Antti Sajantila, Maria F Perdomo

**Affiliations:** Department of Virology, University of Helsinki, Haartmaninkatu 3, Helsinki, 00290, Finland; Department of Electronics, Telecommunications and Informatics, University of Aveiro, Campus Universitario de Santiago, 3810-193 Aveiro, Portugal; Institute of Electronics and Informatics Engineering of Aveiro, University of Aveiro, Campus Universitario de Santiago, 3810-193 Aveiro, Portugal; Department of Virology, University of Helsinki, Haartmaninkatu 3, Helsinki, 00290, Finland; Department of Virology, University of Helsinki, Haartmaninkatu 3, Helsinki, 00290, Finland; Department of Virology, University of Helsinki, Haartmaninkatu 3, Helsinki, 00290, Finland; HUSLAB, Helsinki University Hospital, Topeliuksenkatu 32, 00290 Helsinki, Finland; Department of Forensic Medicine, University of Helsinki, Kytösuontie 11, 00300, Helsinki, Finland; Forensic Medicine Unit, Finnish Institute of Health and Welfare, PO Box 30 FI-00271 Helsinki, Finland; Department of Virology, University of Helsinki, Haartmaninkatu 3, Helsinki, 00290, Finland

**Keywords:** efficient pipeline, multi-organ sequencing, viral genomes, genome analysis, parvovirus B19, JC polyomavirus, mitochondrial DNA

## Abstract

**Background:**

Advances in sequencing technologies have enabled the characterization of multiple microbial and host genomes, opening new frontiers of knowledge while kindling novel applications and research perspectives. Among these is the investigation of the viral communities residing in the human body and their impact on health and disease. To this end, the study of samples from multiple tissues is critical, yet, the complexity of such analysis calls for a dedicated pipeline. We provide an automatic and efficient pipeline for identification, assembly, and analysis of viral genomes that combines the DNA sequence data from multiple organs. TRACESPipe relies on cooperation among 3 modalities: compression-based prediction, sequence alignment, and *de novo* assembly. The pipeline is ultra-fast and provides, additionally, secure transmission and storage of sensitive data.

**Findings:**

TRACESPipe performed outstandingly when tested on synthetic and *ex vivo* datasets, identifying and reconstructing all the viral genomes, including those with high levels of single-nucleotide polymorphisms. It also detected minimal levels of genomic variation between different organs.

**Conclusions:**

TRACESPipe's unique ability to simultaneously process and analyze samples from different sources enables the evaluation of within-host variability. This opens up the possibility to investigate viral tissue tropism, evolution, fitness, and disease associations. Moreover, additional features such as DNA damage estimation and mitochondrial DNA reconstruction and analysis, as well as exogenous-source controls, expand the utility of this pipeline to other fields such as forensics and ancient DNA studies. TRACESPipe is released under GPLv3 and is available for free download at https://github.com/viromelab/tracespipe.

## Introduction

The field of virology has experienced a revolution along with the introduction of next-generation sequencing technologies (NGS) as the number of emerging and newly discovered viruses continues to rise at near-exponential rates. Advantages of NGS over traditional methods include multiplex capability, analytical resolution, and unbiased exploration of microbial metagenomic composition. Thanks to NGS, long-standing questions on the virome and on its interactions with the host can now be investigated. These include the study of the types and genetic diversities of the viral populations residing in different organs of the human body [1]. To this end, the examination of samples from multiple tissues of an individual is essential, yet, the integration and analysis of such data has a high degree of complexity.

Along with its unquestionable impact, NGS has also brought up new challenges due to the volume of data derived. This has rendered necessary the design of automatic workflows, or pipelines, that use high-level algorithms to connect multiple instructions and tools in unique and custom-based architectures. Building a pipeline is far from trivial because multiple factors need to be taken into account, such as sequencing technologies, biological targets, research aims, compatibility between tools, databases, and computational resources.

For processing of virus sequencing data, several pipelines exist (e.g., VIP [2], VirFinder [3], ViromeScan [4], HoloVir [5], iVirus [6], VirMAP [7], FastViromeExplorer [8], and GenomeDetective [9]). However, these tools are not optimized for the analysis of data derived from multiple organs, leaving each tissue to be analysed individually and independently, at the expense of much computational time.

In this article, we describe TRACESPipe, the first NGS pipeline for identification, analysis, and assembly of viral DNA at multi-organ level. For robust mapping, TRACESPipe uses a hybrid approach that combines the results of reference-based and reference-free methods. Moreover, it includes the analysis of human mitochondrial DNA (mitogenomes), a valuable phylogeographycal marker, to assist in the interpretation of viral findings. Additional features include secure transmission and storage of sensitive data, quality controls, DNA damage estimation, and human Y-chromosome analysis.

## Methods

**Figure 1:**
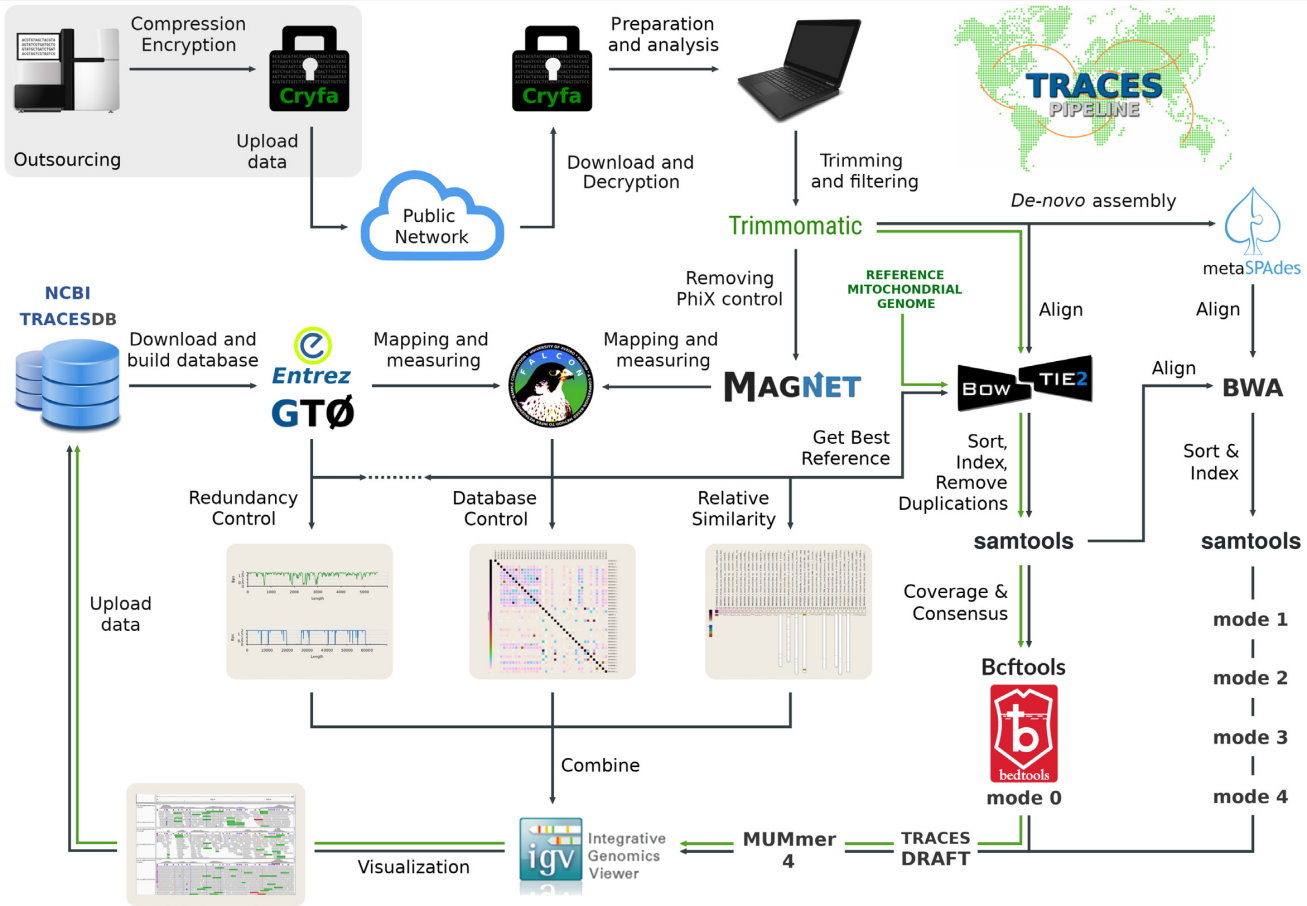
The architecture of TRACESPipe for identification, reconstruction, and analysis of viral and human mitogenomes at multi-organ level. The tools are represented with the respective logos and names. Green arrows indicate mitogenomes, and black arrows indicate viral flowline.

TRACESPipe's workflow (Fig. 1) begins with encryption using Cryfa [10] to protect sensitive information such as human genomic data. This is a unique feature that is not commonly embedded in existing pipelines but is critical when dealing with, e.g., clinical or forensic samples. After quality control, the analysis of viral sequences is driven via 2 parallel approaches: the first one initially applies FALCON-meta [11] to scan the viral reference genomes with highest similarity to the data, followed by alignment of the reads to the identified best references using Bowtie2 [12] and generation of a consensus sequence with Bcftools [13]. The second approach consists of *de novo* assembly (metaSPAdes [14]) to reconstruct *in silico* viral genomes by building scaffolds from overlapping reads. The alignments and scaffolds derived from each approach are at last combined with a competitive alignment-based approach using BWA [15] and global measures to build a high-quality genome draft. Finally, the multi-organ analysis takes places through a sensitive consensus of the available organ data for each virus. Although the pipeline is completely automatic, the multiple intermediary-alignment phases can be interactively supervised with Integrative Genomics Viewer (IGV) [16].

Figure 1 depicts the architecture of TRACESPipe, where green arrows indicate the human mitochondrial flowline. This pipeline has been tested in the analysis of data derived from Illumina HiSeq and NovaSeq platforms. The operating systems required are Linux or Unix. Cygwin [17] can be used as an alternative for Windows operating systems. The installation and configuration procedures, as well as the commands for the runs and structure of the output data, are detailed in Supplementary Section S2 (Reproducibility).

Below we describe the functionalities and options of TRACESPipe, namely, data privacy, storage, preparation, and the creation and maintenance of the viral database. Moreover, we describe the TRACESPipe core, the respective controls, and additional features.

### Data privacy

TRACESPipe provides secure encryption of genomic data using Cryfa [10]. This tool follows industry recommendations for upholding the security of in-transit and at-rest genomic data. Cryfa securely encrypts FASTQ files by a packing transformation after which the information is shuffled and encrypted. The core encryption method uses Advanced Encryption Standard (AES). With this tool TRACESPipe guarantees preservation of the confidentiality, integrity, and authenticity of personal sequencing data.

### Data storage

The amount of data resulting from high-throughput sequencing poses a challenge for its immediate and long-term storage. Possible solutions are to discard non-important data, when possible, and/or data compression [18]. The choice of the compressor always comes with a trade-off between compression capacity and/or speed. We opted for relying substantially upon speed.

In TRACESPipe, all temporary data are erased after use, while permanent data are stored using binary file formats (BAM, Bcf) or compressed with lossless approaches. For the data compression, general-purpose tools (Gzip and Bzip2) as well as Cryfa [10] are used.

### Data preparation

Prior to analysis, the reads need to be trimmed and cleaned from sequence-control genomes (PhiX) and/or reads that are too short, contain sequencing errors, or have low quality scores [19].

TRACESPipe uses Trimmomatic [20] to cut the adapter and other Illumina-specific sequences from the reads. Technically, it removes content from an adapters' list having a maximum mismatch that allows a full match of 2. The palindrome and simple clip threshold are set at 30 and 10, respectively. The minimum quality score required to keep a base at the beginning and at the end are fixed at 3. Also, it is set to filter low-quality data (sliding window of 4 with an average quality of 15). Reads with lengths <25 bases are discarded. This threshold was selected to optimize the analysis of highly fragmented DNA from ancient archaeological or forensic samples; yet, these parameters can be tuned to specific needs.

Moreover, TRACESPipe uses MAGNET [21] to remove reads from the PhiX control below a certain threshold of similarity. In TRACESPipe, MAGNET runs with a mixture of 3 Markov chain models.

### Database

High-quality and diverse viral databases increase the accuracy of reference-based assembly, comparative genomics, and authentication in metagenomics. TRACESPipe uses 4 approaches to create and maintain its own database. The default approach downloads automatically all viral sequences from the nucleotide NCBI database into a multi-FASTA using GTO [22] and Entrez [23] through the accession codes. The second approach downloads NCBI (only) references using the same process. The third approach enables a new genome to be added at any moment using the accession code or a FASTA file, while the fourth permits multiple genomes to be added from a file containing accession codes.

Upon reconstruction of assembled viral sequences, the user has the option of adding them to the TRACES database (using the third approach), to increase the diversity and quality of the database. For the reconstruction and analysis of non-human viral and mitogenomes, TRACESPipe has also enabled the possibility of manually creating the database and selecting the viruses by host or database type, among other features, using the new NCBI viral interface [24].

### TRACESPipe core

The TRACESPipe core assumes that all the previous steps were taken, i.e., the data preparation and database building. The data analysis combines 3 modalities:

- compression-based prediction;
- sequence alignment;
- *de novo* assembly.

The final output is a hybrid approach that merges the viral genome reconstructions derived from these methods.

#### Compression-based prediction

The alignment of FASTQ reads (e.g., from a Novaseq run) to each of the sequences of the NCBI viral database (~200,000) would take months (assuming parallelization). The same task becomes almost unfeasible when analysing multiple FASTQ reads from different organs (it would take years). Therefore, an ultra-fast method that identifies and aligns only the most representative references in the reads is essential.

To scan the reads with highest similarity to the reference database, we use FALCON-meta [11], an alignment-free tool [25]. This tool loads the reads into several Markov and tolerant Markov models [26] under relative compression, and then it freezes those models. Subsequently, it uses context mixing for similarity estimation. Built into this method is the flexibility to account for any polymorphisms or structural variants. The final output is a score representing the similarity of the reads to each reference sequence. The highest similarity values for different categories of viruses are then filtered by name and size, where the highest value stands for the best reference.

TRACESPipe uses FALCON-meta as similarity predictor for single or multiple organs. For the latter, the best reference for each virus is chosen among the most frequent in all the organs.

#### Alignments to the best reference

After assignment of the best reference by FALCON-meta, the reads are aligned using Bowtie2 [12] with very high sensitivity parameters. Extreme sensitivity parameters can also be applied although at substantial cost of computational time. Nevertheless, the analysis can be made with very high sensitivity parameters thanks to the selection of a best reference for each virus, instead of whole alignments to each of the existing viral references (linear vs quadratic complexity).

Subsequently, consensus sequences are built with Bcftools [13] using protocols with specific filters to handle single-nucleotide polymorphisms (SNPs) and indexing support from Tabix [27]. Bases of low quality are designated as N. The variants are stored in BED files using BEDtools [28].

#### De novo assembly

The *de novo* assembly takes place in the pipeline after trimming (data preparation phase) and serves the purposes of validation of the consensus sequences derived from the reference-based alignments and of complementing the viral genome when the reference is only partial or contains high levels of variation. TRACESPipe uses the core meta-assembler of metaSPAdes [14]. This assembler uses an iterative approach to implement a multisized de Bruijn graph algorithm with multiple *k*-mer sizes. The output of metaSPAdes, besides multiple channels of information (such as coverage), is a multi-FASTA file with scaffolds.

#### Hybrid reconstruction

Hybrid methodological approaches in genome assembly, i.e., reference-based combined with *de novo* assembly, provide higher sensitivity and resolution. When the reads are similar to a reference genome, the reference-based approach adds substantially more breadth and depth coverage than the *de novo* assembly, especially at the tips of the scaffolds or contigs. On the other hand, for novel regions or higher concentration of SNPs (or other variations), *de novo* assembly provides complementary information in the absence of aligned reads.

The viral genomes display high diversity [29] in terms of mutation rates and lengths. Thus, reconstruction methods need to efficiently adapt to deliver precise and accurate results. For this purpose, TRACESPipe automatically runs with 5 modes. The first (mode 0) reconstructs a genome exclusively with an alignment-based approach to the best reference, as previously described. This mode is ideal when the number of mutations is very low. The second (mode 1) uses the consensus resulting from the alignments and aligns the *de novo* scaffolds using BWA [15], while giving priority to the former. This approach is suitable for a low to moderate number of mutations. The third (mode 2) is built the same way as mode 1, but the priority is given instead to the *de novo* scaffolds. The alignments are produced with very high sensitivity, forcing the output to be more similar to the *de novo* when the consensus from the alignments is ambiguous or contains gaps. The fourth (mode 3) finds the scaffolds from the *de novo* assembly with highest similarities, as reported by FALCON-meta [21], and uses this as a candidate genome. This mode is ideal when a high-quality genome exists in the sample but has extremely high mutation rates. The fifth (mode 4) uses the scaffolds from mode 3 as reference and aligns the consensus sequence created in mode 1. After applying the 5 modes, TRACESPipe computes the number of bases produced by each mode (that do not contain gaps) and selects the sequence with the highest number of bases.

Although this process is completely automatic, both the alignments and the consensus sequences from all the modes can be visualized in IGV [16]. This way it is possible to detect and compare multi-organ variability, as well as to enable final reconstruction, supervision, and validation by human inspection.

#### Combining multi-organ data

When the within-host variability of viral genomes is very low, complete genome assemblies can be built by merging the consensus sequences from each of the organs. TRACESPipe combines multi-organ data using 2 levels. At the first, the pipeline identifies the most frequent reference among all the organs and forces its use in the analysis. This is essential for human supervision, as well as direct comparison of the data. The latter is then combined at the second level.

After viral reconstruction of each organ, zero-coverage regions can be combined with others of higher depth, from other organs. Hence, an improved and complete genome can be assembled using multiple alignments with very high sensitivity parameters in BWA [15]. Specifically, TRACESPipe enables the production of a consensus of the multi-organ reconstructed data automatically. This feature can be particularly useful in ancient-DNA studies, in which the DNA is frequently fragmented and has a high degree of damage.

### Data controls

The pipeline includes 3 main controls:

- redundancy control;
- database control;
- exogenous control.

These controls are essential to detect the source of abnormal patterns (i.e., high depth [D] with low breadth [B] coverage), excessive number of flagged genomes in the samples, and presence of exogenous genomes.

#### Redundancy control

Redundancy control is a way to estimate duplications or low-complexty regions in the sequences. Repetitive elements on the reference genomes may be over-represented by the same reads. Thus, if 2 regions are very similar, the reads will map to both, creating double the depth coverage. This phenomenon can also be caused by PCR duplicates and sister duplications, in which cases very-high-depth yet low-breadth coverage may be seen.

These events can be minimized by sequencing the flanking regions with longer reads (e.g., with a Pacific Biosciences sequencer), normalization at computational level, or inspection of known repetitive or low-complexity regions together with the depth and breadth coverage profiles. We chose the latter because, besides being very precise and low-cost, it is possible to cross-check the information with similar sub-regions of exogenous content that might be present in the samples.

We use GTO [22] to identify regions of low complexity [30]. It includes a DNA compressor that estimates the content along each genome. We then cross this information with the coverage profiles generated with BEDTools [28] as well as the data from the exogenous control. TRACESPipe includes the possibility of generating coverage profiles, where the depth scale can be set according to a specific value (normalization) for visualization purposes.

Additionally, TRACESPipe uses an optional mode to remove duplications in a traditional way, i.e., using the markdup function from Samtools [31]. When using this option, the alignments will not include reads that have been classified as duplications, instead of only marking them.

#### Database control

The database includes viruses that share high similarity to other family members (e.g., Polyomaviridae) or to the human host (e.g., Herpesviridae). The former may result in high-level mapping of the reads to various references. When the references are full genomes, the mapping automatically finds the best reference; however, when partial genomes are also included, the best reference may be attributed to a partial genome in which only conserved regions are present. To mitigate this, we apply FALCON-meta to measure the cross-similarity between the best references. By default, TRACESPipe uses a threshold of 40 genomes scoring the highest similarities. We found this value to be most optimal in terms of computational time and precision. However, it is flexible and can be modified to higher threshold values (up to 100 are still affordable), at the cost of longer computational time.

Regarding the cross-similarity to human DNA, a small number of reads may be assigned to a reference virus albeit of human origin. We apply FALCON-meta to measure and localize regions of high similarity between the viruses and human reference genome.

#### Exogenous control

Exogenous content, i.e., by fungi, bacteria, or plants, may display low levels of similarity to the viral or mitogenomes [32]. Thus, as a control, TRACESPipe estimates the content of exogenous sequences with FALCON-meta [11] using databases for each respective type. The download and construction of the reference databases are automatically driven by the pipeline using GTO [22] and Entrez [23]. The most representative genomes can be aligned according to the reference, for further consensus sequence construction and analysis.

### Additional features

To assist in the interpretation and analysis of the viral findings, TRACESPipe includes the analysis of human mitogenomes. The reads are aligned exclusively to the revised Cambridge Reference sequence (rCRS) [33, 34] using Bowtie2 [12], and a consensus sequence is generated with Bcftools [13]. Although the human-mitochondrial reference is used by default, TRACESPipe permits the setting of any reference using the genome identifier. Thus, our pipeline is also flexible for the analysis of viruses in other host species.

Also, to control for contamination, TRACESPipe quantifies the Y-chromosome levels through compression-based predictors [11]. The human Y-chromosome reference is compressed relative to the FASTQ reads and subsequently normalized by size in a logarithmic scale. This computation outputs a value between 0 and 1, where values near 1 indicate absence, and near 0, full presence. Additional alignments, consensus sequences, and coverage outputs for the Y-chromosome are available.

Moreover, TRACESPipe has mapDamage2 [35] built in for estimation of DNA damage patterns, i.e., the degree of specific alterations in the tips of the reads. This feature is particularly important in the authentication of ancient DNA.

The pipeline also includes a feature to enable specific alignments using automatic search. These alignments can be made according to a sequence identifier or specific pattern name contained in the database (by a FASTA header pattern). For each match, consensus sequences, variant call files, and coverage profiles are available.

TRACESPipe includes Blastn search [36] to identify the species most likely resembling the query. The database can be consulted locally, through automatic construction, or remotely. One of the applications of Blastn is the identification of the scaffolds derived from *de novo* assembly that do not match any viral or human DNA. This search also enables the finding of potential candidates for novel viruses.

Additional output breadth and depth coverage tables (2D matrix with organ as horizontal and viruses as vertical variables), relative similarity results for each organ, and others can be automatically sent by email (requires email configuration).

TRACESPipe also includes a logging system to record the output provided by each tool as well as debugging messages and system reports, which can be reset at any time.

Finally, there are performance settings, including the specification of the number of threads to be used by the tools. By default, the pipeline calculates and runs with the maximum number of threads available in the system.

## Tools

A compilation of the tools integrated into TRACESPipe with the respective home page and reference is available in Table 1. The installation of these tools is fully automated and provided through Conda using a combination of the channels Bioconda [37] and Cobilab [38].

**Table 1:** Tools integrated into the TRACESPipe with the respective name, home page, and reference.

Name	URL	Reference
Bcftools	www.htslib.org/doc/bcftools.html	[13]
BEDTools	bedtools.readthedocs.io	[28]
Blastn	https://blast.ncbi.nlm.nih.gov/	[36]
Bowtie2	bowtie-bio.sourceforge.net/bowtie2	[12]
BWA	bio-bwa.sourceforge.net/	[15]
Cryfa	github.com/cobilab/cryfa	[10]
Entrez	www.ncbi.nlm.nih.gov/genome	[23]
FALCON-meta	github.com/cobilab/falcon	[11]
GTO	bioinformatics.ua.pt/gto	[22]
IGV	software.broadinstitute.org/software/igv	[16]
MAGNET	github.com/cobilab/magnet	[21]
mapDamage2	ginolhac.github.io/mapDamage	[35]
metaSPAdes	cab.spbu.ru/software/meta-spades	[14]
MUMmer4	https://mummer4.github.io/	[39]
Samtools	samtools.sourceforge.net	[31]
Tabix	htslib.org/doc/tabix.html	[27]
Trimmomatic	www.usadellab.org/cms/?page=trimmomatic	[20]

## Analyses

We tested the performance of TRACESPipe in analysis of synthetic and real data. The synthetic data were generated using viral and mitogenomes to which specific additional exogenous content and mutation rates had been applied. The *ex vivo* data include DNA sequences from different organs collected in connection with post-mortem investigations. The procedure can be replicated using the instructions provided in the Supplementary Material, Reproduciblility section.

### Synthetic data

To test TRACESPipe's efficiency in reconstructing genomes, we created 10 datasets containing several reference viruses and mitogenomes with specified mutation rates. Then, we simulated the sequencing process with ART [40], configured to mimic reads from Illumina HiSeq 2500, paired-end data, and read length of 150. The fragmentation was defined at 200, and the deviation, at 10. The mutation rate, i.e., the simulation of specific SNP percentages, was set with GTO toolkit [22]. The conditions used are described in Table 2. After using TRACESPipe for genome reconstruction, we used dnadiff from the MUMmer4 package [39] to evaluate the identity and number of SNPs between the original and the reconstructed sequences. The breadth and depth coverage of the alignments are described in Supplementary Table S1.

**Table 2:** Benchmark of TRACESPipe in viral and mitogenomes assembly from 10 different organs using FASTQ data simulated with different SNPs and coverage rates; simulation using ART and GTO.

Sequence	Blood			Bone			Brain			Hair			Heart			Kidney			Liver			Lung			Skin			Teeth		
	F	D	S	F	D	S	F	D	S	F	D	S	F	D	S	F	D	S	F	D	S	F	D	S	F	D	S	F	D	S
B19V																														
Simulation	$\checkmark$	40	0	$\checkmark$	30	1	$\checkmark$	10	0	✗			$\checkmark$	20	20	$\checkmark$	20	0	✗			✗			$\checkmark$	25	0	$\checkmark$	30	5
Evaluation	$\checkmark$	100	0	$\checkmark$	100	2	$\checkmark$	100	0	✗	0	0	$\checkmark$	100	0	$\checkmark$	100	0	✗	0	0	✗	0	0	$\checkmark$	100	0	$\checkmark$	99.9	5
HHV2																														
Simulation	$\checkmark$	40	0	✗			✗			✗			✗			$\checkmark$	20	0	✗			✗			✗			$\checkmark$	30	0
Evaluation	$\checkmark$	100	0	✗	0	0	✗	0	0	✗	0	0	✗	0	0	$\checkmark$	100	0	✗	0	0	✗	0	0	✗	0	0	$\checkmark$	100	0
HHV3																														
Simulation	$\checkmark$	40	0	✗			✗			✗			✗			✗			✗			✗			$\checkmark$	25	0	✗		
Evaluation	$\checkmark$	100	0	✗	0	0	✗	0	0	✗	0	0	✗	0	0	✗	0	0	✗	0	0	✗	0	0	$\checkmark$	100	0	✗	0	0
HHV4																														
Simulation	✗			✗			$\checkmark$	10	0	$\checkmark$	5	0	✗			✗			$\checkmark$	20	1	$\checkmark$	10	1	✗			✗		
Evaluation	✗	0	0	✗	0	0	$\checkmark$	99.9	2	$\checkmark$	98.8	11	✗	0	0	✗	0	0	$\checkmark$	99.9	264	$\checkmark$	99.8	286	✗	0	0	✗	0	0
HHV8																														
Simulation	$\checkmark$	40	0	✗			✗			✗			✗			✗			✗			✗			✗			✗		
Evaluation	$\checkmark$	100	0	✗	0	0	✗	0	0	✗	0	0	✗	0	0	✗	0	0	✗	0	0	✗	0	0	✗	0	0	✗	0	0
HPV																														
Simulation	✗			✗			✗			$\checkmark$	5	10	$\checkmark$	20	10	✗			$\checkmark$	20	0	✗			✗			✗		
Evaluation	✗	0	0	✗	0	0	✗	0	0	$\checkmark$	98.8	0	$\checkmark$	100	0	✗	0	0	$\checkmark$	100	0	✗	0	0	✗	0	0	✗	0	0
TTV																														
Simulation	✗			$\checkmark$	30	10	✗			✗			✗	−	−	$\checkmark$	20	15	✗			✗			$\checkmark$	25	0	$\checkmark$	30	0
Evaluation	✗	0	0	$\checkmark$	100	0	✗	0	0	✗	0	0	✗	0	0	$\checkmark$	100	0	✗	0	0	✗	0	0	$\checkmark$	100	0	$\checkmark$	100	0
VARV																														
Simulation	$\checkmark$	40	0	✗			$\checkmark$	10	0	✗			$\checkmark$	20	5	✗			$\checkmark$	20	0	$\checkmark$	10	0	✗			✗		
Evaluation	$\checkmark$	100	0	✗	0	0	$\checkmark$	99.9	2	✗	0	0	$\checkmark$	100	21	✗	0	0	$\checkmark$	100	0	$\checkmark$	99.9	1	✗	0	0	✗	0	0
MT																														
Simulation	$\checkmark$	40	0	$\checkmark$	30	0	$\checkmark$	10	1	$\checkmark$	5	0	$\checkmark$	20	0	$\checkmark$	20	1	$\checkmark$	20	2	$\checkmark$	10	0	$\checkmark$	25	0	$\checkmark$	30	5
Evaluation	$\checkmark$	100	0	$\checkmark$	100	0	$\checkmark$	99.5	1	$\checkmark$	98.8	1	$\checkmark$	99.9	0	$\checkmark$	99.9	0	$\checkmark$	99.9	0	$\checkmark$	99.5	1	$\checkmark$	100	0	$\checkmark$	99.7	0

The grey background is the statistical ground truth (simulation conditions), while the white background represents the evaluation of TRACESPipe output using dnadiff. The F indicates the existence or not of the respective virus in the organ, where the check mark indicates viral or mitogenome detection in the sample, and ✗, the opposite. For the simulation conditions (grey background), the D stands for depth coverage and S for the applied percentage of SNPs. For the evaluation (white background), the D stands for the identity and S for the number of SNPs found after full genome reconstruction. The genome sequences were as follows: B19V: parvovirus B19; HHV: human herpesvirus (multiple types); HPV: human papillomavirus; TTV: torque teno virus; VARV: variola virus; and MT: human mitogenome. This experiment can be replicated using the script Benchmark.sh.

In some of the viruses and mitogenomes, up to 20% synthetic mutations were introduced, representing on average 20 SNPs per 100 bases. The whole experiment, including the automatic reconstruction of all genomes, took ~10 minutes on a laptop computer.

**Figure 2:**
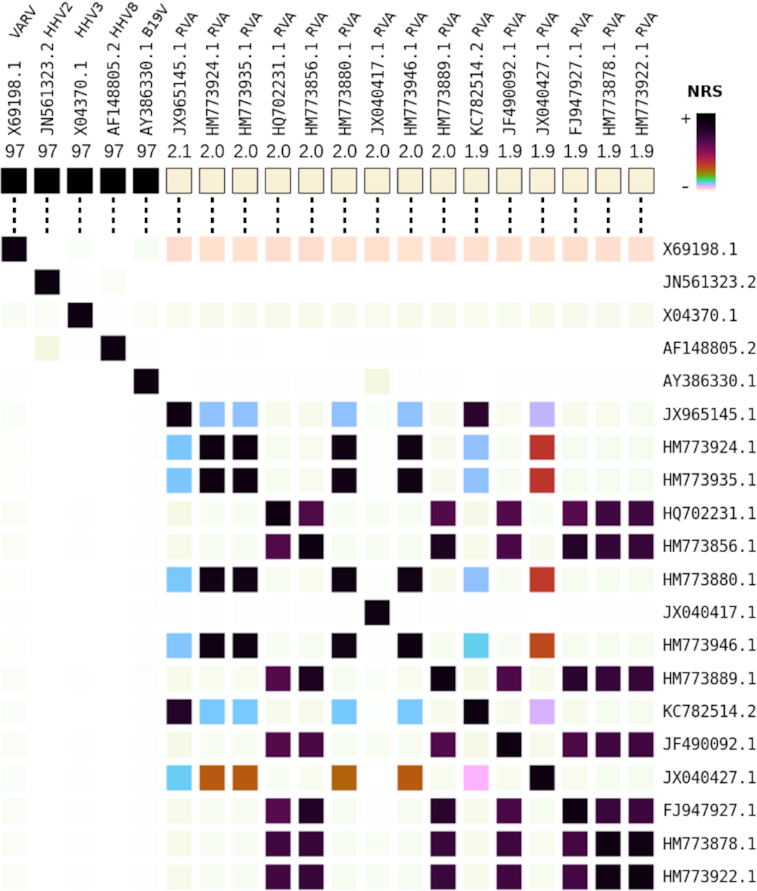
Normalized relative similarity (NRS) for the synthetic blood sample provided by TRACESPipe. The upper map depicts the highest NRS values of the reads according to the references and the lower map the cross-similarity between each reference pair.

As described in the methodology, after trimming and filtering, TRACESPipe proceeds with FALCON-meta [11] to find the best virus references for each organ sample. Figure 2 (upper map) shows an example of the output after candidate reference discovery. Here, the candidates were variola virus (VARV), human herpesvirus (HHV)2, HHV3, HHV8, and human parvovirus B19 (B19V) with normalized relative similarity (NRS) values >96%, while the remaining were ~2%. The bottom map of Figure 2 shows the similarities between candidate pairs. This is critical to detect low-level similarities between the respective references. In blood, the 5 genomes were easily detected. Subsequently, the reads were aligned to the best reference for each genome, a consensus sequence created and combined with *de novo* assembled scaffolds.

**Figure 3:**
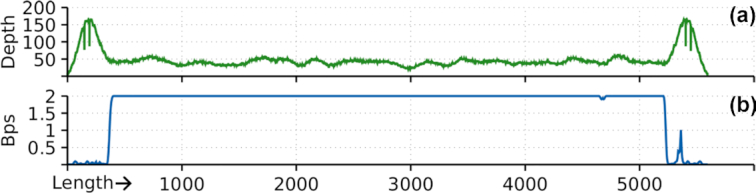
Redundancy controls with coverage (a) and complexity (b) profiles for a B19V DNA sequence. Depth indicates depth coverage, and Bps, bits per base. Lower Bps values mean higher redundancy. The length scale is in nucleotides.

We found that the highest SNP values reported by dnadiff were for HHV4. These corresponded to regions that are very complex to assemble, both by alignment-based and *de novo* approaches, owing to statistical ambiguity created by the sequencing noise (multiple repeats and regions of low complexity; Fig S4). Because the genome is near 170 kb long, the number of SNPs easily increases when noise or mutations are added. Moreover, in some cases, we found slightly higher coverage values than those simulated in the intermediary state of alignments (Supplementary Table S1). These were given by similarity between different regions because we opted neither to normalize the coverage nor to apply any equivalent method but instead to use complexity analysis after duplicate removal. Accordingly, we crossed the complexity profiles with the coverage profiles. In the tips of the B19V genome 2 areas of high depth coverage were distinguishable, the inverted terminal repeats (ITRs), classified as low by our complexity analysis. An example of this analysis is presented in Fig. 3, with the B19V DNA identified in blood.

When assessing the individual identity percentage (D—top sub-row of Table 2), for a simulated coverage depth of 40 (D—bottom sub-row of Table 2), we recovered full genomes without any alterations. Yet, even at low-depth coverage and high levels of SNPs, TRACESPipe was able to reconstruct the genomes with excellent identity. The lowest values were found for HHV4 in hair and liver (98.6). The hair dataset was simulated with 5× coverage, re-creating regions with gaps or base call ambiguity, while the lung was simulated with 10× coverage and 1% of random mutations. A high–mutation rate test was conducted for TTV in kidney, in which a 20× coverage and a 15% mutation rate were simulated. Also in this case, TRACESPipe was able to reconstruct the genome with 100% identity and without SNPs in relation to the original sequence. Furthermore, an extreme test was run for B19V in the heart, in which a 20× coverage and 20% mutation rate (1 SNP every 5 bases) were mimicked. Despite these conditions, TRACESPipe was able to efficiently reconstruct the B19V genome, showing an identity of 100% without SNPs according to the original sequence. For a representation of the dissimilarities between 0% and 1% SNPs, see Supplementary Fig. S1.

As reported in Table 2, all the viral and mitogenomes in the samples were identified and efficiently reconstructed (without false-positive results). A FASTA sequence for each genome was generated along with the necessary controls.

In addition, we evaluated the automatic detection and reconstruction of hybrid viral genomes (defined as combinations of viral sequences). For this purpose, we re-created concatenations of extractions from B19V and VARV sequences using different mutation rates in blood, brain, and bone. The simulation process presented in HybridSpecies.sh is described in Supplementary Section S2. Thereafter, we simulated the sequencing process as previously described and evaluated the differences between the original hybrid and the reconstructed genomes. The results are presented in Supplementary Table S2, showing full reconstruction with 100% identity.

Together, these results prove the efficiency of TRACESPipe in the identification and reconstruction of viral and human mitochondrial genomes, at multi-organ level, even when prompted with low coverage and high mutation rates.

### Real data

**Figure 4:**
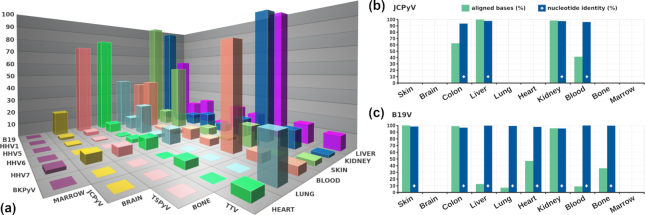
(a) Breadth coverage percentage (z-axis) of the (real) mapped reads against the best reference virus for each organ sample. The plot is restricted to viral types with a minimum similarity of 10% in ≥1 of the organs. The bottom corner had shallow values, which due to space constraints were not included. (b,c) Percentage of aligned bases (green) and nucleotide identity (blue) between the best reference and reconstructed genomes of JCPyV and B19V, respectively, calculated using dnadiff. Low breadth coverages may not have corresponding aligned-data values as they may have fallen under the minimal quality or similarity thresholds. The latter was set before the run to exclude noise.

**Figure 5:**
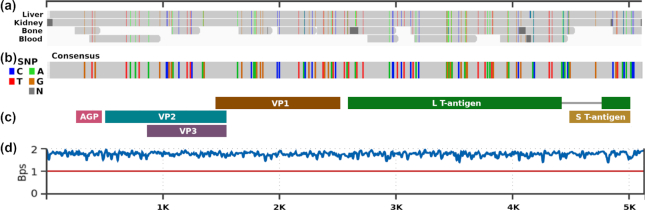
Visualization of the consensus alignments of JCPyV, with the basic structure and complexity profile. (a) JCPyV consensus sequences from 4 organs aligned to the U61771.1 reference using BWA. Vertical lines indicate SNPs with the respective nucleotide. The dark grey regions indicate gaps (N); (b) final consensus sequence merged from (a), with SNPs thickened for visualization purposes; (c) JCPyV structure with main proteins; (d) complexity profile; Bps values <1 correspond to repetitive data. The JCPyV consensus sequences were computed after duplicate removal. The (a,b) maps were adapted from the IGV after TRACESPipe computation. Bps: bits per base.

**Figure 6:**
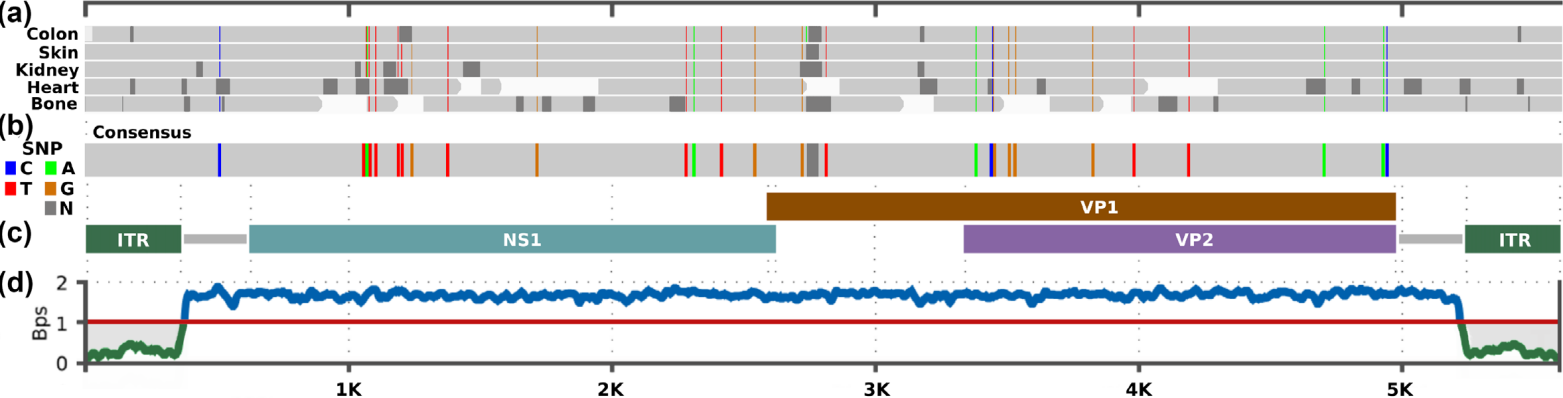
Visualization of the consensus alignments for B19V with the basic structure and complexity profile. (a) B19V consensus sequences from 5 organs aligned to the KM393164.1 reference using BWA. Vertical lines indicate SNPs with the respective nucleotide. The dark grey regions indicate gaps (N); (b) final consensus sequence built from (a), with SNPs thickened for visualization purposes; (c) B19V structure including main proteins and inverted terminal repeats (ITRs); (d) complexity profile, where lower regions (ITR) represent repetitive data (bits per base [Bps] < 1). The (a,b) maps were adapted from the IGV after TRACESPipe computation.

We tested the performance of TRACESPipe in the identification of viral DNA reads derived from different tissues of a recently deceased individual. The organs analyzed were bone, bone marrow, brain, heart, kidney, liver, lung, blood, and skin. Each sample was processed individually in the laboratory prior to sequencing in Novaseq (Illumina) with 150 paired-end reads. After de-multiplexing, the sequenced reads were split according to the organ of origin. TRACESPipe identified several genomes, of which JC polyomavirus (JCPyV) (Fig. 5), B19V (Fig. 6), and the human mitogenome are here presented as examples. The percentages of breadth coverage of the mapped reads against the best reference for each organ are depicted in Fig. 4a, together with the percentage of aligned bases and nucleotide identity for JCPyV and B19V in Fig. 4b and c, respectively. The alignments of the reads for JCPyV and the human mitogenome in selected organs can be seen in Supplementary Fig. S3.

A Blastn [41] search of the generated consensus sequences of JCPyV from kidney and liver showed an average nucleotide identity of 99% (only few gaps). All the SNPs were congruent between organs, with high coverage. In the skin, the number of reads that aligned to the reference was insufficient; yet, identical SNPs were detected. Figure 5 depicts the alignments and consensus of JCPyV for the organs with highest identity along with the genome map and complexity profile. JCPyV does not contain large redundant parts, enabling easier reconstruction of the complete genome.

Similar analysis was performed for B19V (Fig. 6), which displayed fewer SNPs than did JCPyV. Also in this case, B19V showed extremely low DNA variability between organs, allowing for reconstruction of a full consensus sequence derived from the merging of each of the organ sequences.

The mitogenome consensus sequences of 10 organs were almost identical (Supplementary Fig. S2). The only exception was colon, in which a single SNP was absent. We verified that the area where this SNP was located was only covered minimally. Thus, if we had relied on the data from colon exclusively, this mutation would have been missed. This finding emphasizes the benefits of comparing the data from different organs as part of the validation process. The final consensus sequence, derived from all the organs, showed 100% in both identity and aligned bases and the presence of 18 SNPs with respect to the reference (Supplementary Fig. S5 includes the positions and variations).

The genomes of B19V, JCPyV, and human mitogenome were fully assembled with coverages of >25×, >40×, and >80×, respectively. These genomes are available as supplementary material and were uploaded into the TRACES database, freely available as FASTA format files [42]. Additionally, these genomes have been updated into the GenBank with the following accession codes: MT682520 (B19V), MT682521 (JCPyV), MT682522 (mitogenome). The reads to generate the analysis are available in SRA under the code PRJNA644600.

The congruent patterns of SNPs across multiple tissues, both for the viral (B19V and JCPyV) and mitogenomes, suggests that the within-host varibility is minimal. This confers an advantage for the final output of the data, in terms of quality and resolution, and demonstrates the value of this pipeline in combining the information at the multi-organ level.

## Conclusions

TRACESPipe is an automatic and efficient pipeline for the reconstruction and analysis of viral genomes. It profits from the synergy between reference-based and reference-free approaches to increase the quality and certainty of prediction to a high level. Indeed, the pipeline performed outstandingly in assignment and reconstruction of viral genomes even when high mutation rates were simulated.

As a unique feature, it supports the merging of data from multiple organ samples. This gives an advantage over existing tools by permitting the evaluation of intra-host genomic diversity. In terms of the viral populations persisting in the body, this opens the way for the investigation of a diverse range of topics, such as viral tissue tropism, evolution, fitness, and disease associations.

Moreover, the extremely low within-host variability of viral genomes and human mitogenomes in different organs, as observed here, may signify an advantage for efficient and complete genome assembly. In fact, the quality of data could be significantly improved by merging complementary sequencing reads between organs towards a robust sequence genome. This may be particularly useful in the scenario of highly fragmented DNA samples, with genomic regions missing, degraded, or with high-degree damage, as is frequently the case for ancient DNA.

Another special component of TRACESPipe is the analysis of mitogenomes. Besides serving as a control for external contamination, the cross-association of the viral types with the geographical distribution of this marker can be extremely valuable in epidemiological or archaeovirological studies, as well as in forensic investigations, to evaluate the origins of unidentified individuals [43,44].

Additional features such as encryption and numerous quality and contamination controls make TRACESPipe a robust tool for comprehensive analysis of genomic data.

## Availability of Source Code and Requirements

- Project name: TRACES Pipeline
- Home page: https://github.com/viromelab/tracespipe
- Operating system(s): Linux/Unix
- Programming language: Shell
- License: GNU GPL3
- RRID:SCR_018831
- Biotools: tracespipe

## Availability of Supporting Data and Materials

Raw data are available at the SRA [45] (PRJNA644600). Genome assembly data products are available at the GenBank [46] with the codes MT682520 (B19V), MT682521 (JCPyV), and MT682522 (mitogenome). All supporting data and materials are available at the *GigaScience* database (GigaDB) [47].

## Additional Files

**Supplementary Figure S1**. Alignments using Bowtie2 of simulated mitochondrial reads relative to the reference genome. a) the sequence is mutated with 1% substitutions in a brain sample, simulated with 10x depth coverage; b) sequence without mutations from a bone sample with a simulated depth coverage of 30. The identified SNPs are highlighted with vertical colored stripes. Visual map extracted from IGV.

**Supplementary Figure S2**. Alignments using BWA of the consensus sequences of real mitochondrial sequences from multiple organs. The identified SNPs are highlighted with vertical colored stripes. Consensus SNPs: 72 T→A, 93 A→G, 263 A→G, 309 +CT, 722 C→A, 750 A→G, 1438 A→G, 2706 A→G, 3106 -C, 3549 C→T, 4580 G→A, 4769 A→G, 7028 C→T, 7444 G→A, 8860 A→G, 11899 T→C, 15326 A→G, 15904 C→T, 16153 G→A, 16298 T→C. Visual map extracted from IGV.

**Supplementary Figure S**3. Visualization of the read alignments of each organ sample for JCPyV and the human mitogenome (MT). The maps have been adapted from the IGV. Each map shows the complete alignments in the respective scale. The JCPyV maps include duplicate removal while the MT maps contain the duplications, as an example of TRACESPipe being able to filter or maintain the duplications (reads highlighted green by IGV show possible duplications). Vertical lines stand for SNPs. Red reads refer to an inferred insert size that is larger than expected (possible evidence of a deletion).

**Supplementary Figure S**4. Complexity profiles for several Human Herpesvirus (HHV1, HHV2, HHV3, HHV4, and HHV5). Lower regions correspond to close or distant repetitive regions. The profiles were computed with TRACESPipe using GTO.

**Supplementary Figure S**5. a) Number of SNPs; b) percentage of aligned bases (green) and nucleotide identity (blue) of the human mitogenome reference relative to the reconstructed sequence. The number of SNPs, percentage of aligned bases, and nucleotide identity have been automatically computed with TRACESPipe using dnadiff from the Mummer4 package.

**Supplementary Table S1**. Benchmark of TRACESPipe (depth and breadth coverage) in viral and mitogenomes from 10 different organs. In each SEQ line, the upper line is the statistical ground truth (simulation conditions), while the bottom line represents the TRACESPipe output. The F stands for the existence or not of the respective virus in the organ sample, where the correct symbol stands for viral or mitochondrial genome detection in the sample, while the x symbol for the opposite. The D stands for the depth coverage and S for the breadth coverage. To replicate use script Benchmark.sh from the repository.

**Supplementary Table S2**. Benchmark of TRACESPipe when hybrid viral species with mutated parts are present in the data. The FASTQ data was simulated with ART. The aligned bases, genome identity and numbers of SNPs refer to the comparison between the original and reconstructed sequences. To replicate use script HybridSpecies.sh from the repository.

giaa086_GIGA-D-20-00018_Original_Submission

giaa086_GIGA-D-20-00018_Revision_1

giaa086_GIGA-D-20-00018_Revision_2

giaa086_GIGA-D-20-00018_Revision_3

giaa086_Response_to_Reviewer_Comments_Original_Submission

giaa086_Response_to_Reviewer_Comments_Revision_1

giaa086_Response_to_Reviewer_Comments_Revision_2

giaa086_Reviewer_1_Report_Original_Submission Brett E. Pickett, Ph.D. -- 2/13/2020 Reviewed

giaa086_Reviewer_2_Report_Original_Submission Saima Sultana Tithi, Ph.D. -- 2/22/2020 Reviewed

giaa086_Reviewer_2_Report_Revision_1 Saima Sultana Tithi, Ph.D. -- 6/17/2020 Reviewed

giaa086_Supplemental_File

## Abbreviations

BLAST: Basic Local Alignment Search Tool; BWA: Burrows-Wheeler Aligner; B19V: human parvovirus B19; GPL: GNU Public License; HPV: human papillomavirus; HHV: human herpesvirus; IGV: Integrative Genomics Viewer; ITR: inverted terminal repeat; JCPyV: JC polyomavirus; MT: mitogenome; NCBI: National Center for Biotechnology Information; NGS: next-generation sequencing; NRS: normalized relative similarity; SNP: single-nucleotide polymorphism; TTV: torque teno virus; VARV: variola virus; VCF: variant call format.

## Ethical Approval

The study using tissues from autopsies performed at the Department of Forensic Medicine of Helsinki University was reviewed by the Ethics Committee of the Helsinki and Uusimaa Hospital district, dossier No. 164/13/03/00/114.

## Competing Interests

The authors declare that they have no competing interests.

## Funding

This work was partially funded by national funds through the FCT in the context of the project UIDB/00127/2020. Also by the Finnish Medical Foundation, Finnish Cultural Foundation, Juhani Aho Foundation for Medical Research, Jane ja Aatos Erkon Säätiö, Medicinska Understödsföreningen Liv och Hälsa, Kone Foundation, Magnus Ehrnrooth Foundation, the Finnish Society of Sciences and Letters, the Research Funds of University of Helsinki and Helsinki University Hospital. D.P. is funded by national funds through FCT - Fundação para a Ciência e a Tecnologia, I.P., under the Scientific Employment Stimulus - Institutional Call - CI-CTTI-94-ARH/2019.

## Authors' Contributions

D.P., A.S., and M.P. conceived and designed the experiments; D.P., M.T., and L.P. performed the experiments; D.P., M.T., L.P., K.H., A.S., and M.P. analyzed the data; D.P., M.T., L.P., K.H., A.S., and M.P. wrote the manuscript.
