# Supplementary material for: A hybrid pipeline for reconstruction and analysis of viral genomes at multi-organ level
Source: Gigascience. 2020 Aug 20;9(8):giaa086. doi: 10.1093/gigascience/giaa086 (PMC7439602; doi:10.1093/gigascience/giaa086)
Supplement: giaa086_GIGA-D-20-00018_Original_Submission [file giaa086_giga-d-20-00018_original_submission.pdf]

GigaScience

A hybrid pipeline for reconstruction and analysis of viral genomes at multi-organ level

--Manuscript Draft--

Manuscript Number:	GIGA-D-20-00018
Full Title:	A hybrid pipeline for reconstruction and analysis of viral genomes at multi-organ level
Article Type:	Technical Note
Funding Information:	Fundação para a Ciência e a Tecnologia (PTCD/EEI-SII/6608/2014) — PhD Diogo Pratas<br>Fundação para a Ciência e a Tecnologia (UID/CEC/00127/2014) — PhD Diogo Pratas<br>Suomen Lääketieteen Säätiö (FI) — Not applicable<br>Jane ja Aatos Erkon Säätiö — Not applicable<br>Medicinska Understödsföreningen Liv och Hälsa — Not applicable<br>Suomalainen Tiedeakatemia — Not applicable<br>Helsingin Yliopiston Tiedesäätiö — Not applicable<br>Juhani Aho Foundation for Medical Research — Not applicable
Abstract:	Background: Advances in sequencing technologies have enabled the characterization of multiple microbial and host genomes, opening new frontiers of knowledge while kindling novel applications and research perspectives. Among these, is the investigation of the viral communities residing in the human body and their impact on health and disease. To this end, the study of samples from multiple tissues is critical, yet, the complexity of such analysis calls for a dedicated pipeline. We provide an automatic and efficient pipeline for identification, assembly and analysis of viral genomes, that combines the DNA sequence data from multiple organs.<br>TRACESPipe relies on cooperation between three modalities: compression-based prediction, sequence alignment, and de-novo assembly. The pipeline is ultra-fast and provides, additionally, secure transmission and storage of sensitive data.<br>Findings: TRACESPipe performed outstandingly when tested on synthetic and ex-vivo datasets, identifying and reconstructing all the viral genomes, including those with high levels of single nucleotide polymorphisms, as well as detecting even minimal levels of genomic variation between different organs.<br>Conclusions: TRACESPipe introduces the possibility to evaluate within-host variability with its uniqueness to process and analyze simultaneously samples from different sources. This opens up the possibility to investigate viral tissue tropism, evolution, fitness and disease associations. Moreover, additional features such as DNA damage estimation, mitochondrial DNA analysis and exogenous-source controls expand the utility of this pipeline to other fields such as forensics and ancient DNA studies.<br>TRACESPipe is released under GPLv3 and is available for free download at https://github.com/viromelab/tracespipe.
Corresponding Author:	Diogo Pratas<br><br>PORTUGAL
Corresponding Author Secondary Information:	
Corresponding Author's Institution:	
Corresponding Author's Secondary Institution:	
First Author:	Diogo Pratas
First Author Secondary Information:	
Order of Authors:	Diogo Pratas

Powered by Editorial Manager® and ProduXion Manager® from Aries Systems Corporation

	Mari Toppinen
	Lari Pyöriä
	Klaus Hedman
	Antti Sajantila
	Maria Perdomo
**Order of Authors Secondary Information:**	
**Additional Information:**	
**Question**	**Response**
Are you submitting this manuscript to a special series or article collection?	No
**Experimental design and statistics**<br><br>Full details of the experimental design and statistical methods used should be given in the Methods section, as detailed in our Minimum Standards Reporting Checklist. Information essential to interpreting the data presented should be made available in the figure legends.<br><br>Have you included all the information requested in your manuscript?	Yes
**Resources**<br><br>A description of all resources used, including antibodies, cell lines, animals and software tools, with enough information to allow them to be uniquely identified, should be included in the Methods section. Authors are strongly encouraged to cite Research Resource Identifiers (RRIDs) for antibodies, model organisms and tools, where possible.<br><br>Have you included the information requested as detailed in our Minimum Standards Reporting Checklist?	Yes
**Availability of data and materials**<br><br>All datasets and code on which the conclusions of the paper rely must be	Yes

either included in your submission or deposited in publicly available repositories (where available and ethically appropriate), referencing such data using a unique identifier in the references and in the “Availability of Data and Materials” section of your manuscript.<br><br>Have you have met the above requirement as detailed in our Minimum Standards Reporting Checklist?	

Powered by Editorial Manager® and ProduXion Manager® from Aries Systems Corporation

Manuscript Click here to download Manuscript main.tex

OXFORD (GIGA)^n SCIENCE

GigaScience, 2020, 1–8

doi: xx.xxxx/xxxx
Manuscript in Preparation
TECHNICAL NOTE

TECHNICAL NOTE

A hybrid pipeline for reconstruction and analysis of viral genomes at multi-organ level

Diogo Pratas[1,4,5,*], Mari Toppinen[1], Lari Pyöriä[1], Klaus Hedman[1,3], Antti Sajantila[2,*] and Maria F. Perdomo[1,*]

[1]Department of Virology, University of Helsinki, Finland and [2]Department of Forensic Medicine, University of Helsinki, Finland and [3]HUSLAB, Helsinki University Hospital, Finland and [4]Department of Electronics Telecommunications and Informatics, University of Aveiro, Portugal and [5]Institute of Electronics and Informatics Engineering of Aveiro, University of Aveiro, Portugal

[*]pratas@ua.pt; antti.sajantila@helsinki.fi; maria.perdomo@helsinki.fi

Abstract

Background: Advances in sequencing technologies have enabled the characterization of multiple microbial and host genomes, opening new frontiers of knowledge while kindling novel applications and research perspectives. Among these, is the investigation of the viral communities residing in the human body and their impact on health and disease. To this end, the study of samples from multiple tissues is critical, yet, the complexity of such analysis calls for a dedicated pipeline. We provide an automatic and efficient pipeline for identification, assembly and analysis of viral genomes, that combines the DNA sequence data from multiple organs. *TRACESPipe* relies on cooperation between three modalities: compression-based prediction, sequence alignment, and *de-novo* assembly. The pipeline is ultra-fast and provides, additionally, secure transmission and storage of sensitive data. **Findings:** TRACESPipe performed outstandingly when tested on synthetic and ex-vivo datasets, identifying and reconstructing all the viral genomes, including those with high levels of single nucleotide polymorphisms, as well as detecting even minimal levels of genomic variation between different organs. **Conclusions:** TRACESPipe introduces the possibility to evaluate within-host variability with its uniqueness to process and analyze simultaneously samples from different sources. This opens up the possibility to investigate viral tissue tropism, evolution, fitness and disease associations. Moreover, additional features such as DNA damage estimation, mitochondrial DNA analysis and exogenous-source controls expand the utility of this pipeline to other fields such as forensics and ancient DNA studies. TRACESPipe is released under GPLv3 and is available for free download at https://github.com/viromelab/tracespipe.

Key words: efficient pipeline; multi-organ sequencing; viral genomes; genome analysis; parvovirus B19; JC polyomavirus; mitochondrial DNA

Introduction

The field of virology has experienced a revolution along with the introduction of next generation sequencing technologies (NGS) as the number of emerging and newly discovered viruses continues to rise at near exponential rates. Advantages of NGS over traditional methods include multiplex capability, analytical resolution and unbiased exploration of microbial metagenomic composition. Thanks to NGS, long standing questions on the virome and on its interactions with the host can now be investigated. This includes the study of the types and genetic diversities of the viral populations residing in different organs of the human body [1]. To this end, the examination of samples from multiple organs of an individual is essential, yet, the integration and analysis of such data has a high degree of complexity.

Compiled on: January 17, 2020.
Draft manuscript prepared by the author.

Along with its unquestionable impact, NGS has also brought up new challenges due to the volume of data from it derived. This has rendered necessary the design of automatic workflows, or pipelines, that use high-level algorithms to connect multiple instructions and tools in unique and custom-based architectures. Building a pipeline is far from trivial as multiple factors need to be taken into account such as sequencing technology, biological targets, research aim, compatibility between tools, databases and computational resources.

For processing of virus sequencing data, several pipelines exist (e.g. VIP [2], VirFinder [3], ViromeScan [4], HoloVir [5], FastViromeExplorer [6] and GenomeDetective [7]). However, these tools are not optimized for the analysis of data derived from multiple-organ samples, leaving each tissue to be analysed individually and independently, at the expense of much computational time.

In this article, we describe TRACESPipe, the first next-generation sequencing pipeline for identification, analysis and assembly of viral DNA at multi-organ level. For robust mapping, TRACESPipe uses a hybrid approach that combines the results of reference -based and -free methods. Moreover, it includes the analysis of human mitochondrial DNA (mtDNA), a valuable marker with geographical pattern, to assist in the interpretation of viral findings. Additional features include secure transmission and storage of sensitive data, quality controls, DNA damage estimation and human Y-chromosome analysis.

Methods

TRACESPipe' workflow (Figure 1) begins with encryption using Cryfa [8] to protect sensitive information such as human genomic data. This is a unique feature not commonly embedded in existing pipelines but one that is critical when dealing with e.g. clinical or forensic samples. After quality control, the analysis of viral sequences is driven via two parallel approaches: the first one, applies initially FALCON-meta [9] to scan the viral reference genomes with highest similarity to the data, followed by alignment of the reads to the identified best references using Bowtie2 [10] and generation of a consensus sequence using BCFtools [11]. The second approach consists of *de-novo* assembly (metaSPAdes [12]) that reconstructs *in silico* viral genomes by building scaffolds from overlapping reads. The alignments and scaffolds derived from each approach are at last combined to build a high quality genome draft. The final construct is interactively supervised with Integrative Genomics Viewer (IGV) [13].

Figure 1 depicts the architecture of TRACESPipe, where the green line stands for the human mitochondrial flowline. This pipeline has been tested in the analysis of data derived from Illumina HiSeq and NovaSeq platforms. The operating systems required are Linux or Unix. The cygwin (https://www.cygwin.com/) can be used as an alternative for Windows operating systems.

Below we describe the functionalities and options of TRACESPipe, namely data privacy, storage, preparation, and the creation and maintenance of the viral database. Moreover, we describe the TRACESPipe core, the respective controls and additional features.

Data privacy

TRACESPipe provides secure encryption of genomic data using Cryfa [8]. This tool follows industry recommendations for upholding the security of in-transit and at-rest genomic data. Cryfa securely encrypts FASTQ files by a block transformation after which the information is shuffled and re-encrypted. With this tool TRACESPipe guarantees the preservation of the confidentiality, integrity, and authenticity of personal sequencing data.

Data storage

The amount of data resulting from high throughput sequencing poses a challenge for its immediate and long-term storage. Possible solutions to alleviate this are to discard non-important data, when possible, and/or data compression [14]. The choice of the compressor always comes with a trade-off between compression capacity and/or speed. We opted for relying substantially upon speed.

In TRACESPipe, all temporary data are erased after use, while permanent data are stored using binary file formats (BAM, Bcf) or compressed with lossless approaches. For the data compression, general purpose tools (Gzip and Bzip2) as well as Cryfa [8] are used.

Data preparation

Prior to analysis, the reads need to be trimmed and cleaned from sequence-control genomes (Phix) and/or reads that are too short, contain sequencing errors or have low quality scores [15].

TRACESPipe uses Trimmomatic [16] to cut the adapter and other Illumina-specific sequences from the reads. Technically, it removes content from an adapters' list having a maximum mismatch that allows a full match of 2. The palindrome and simple clip threshold are set at 30 and 10, respectively. The minimum quality-score required to keep a base at the beginning and end are set at 3. Also, it is set to filter low-quality data (sliding window of 4 with an average quality of 15). Reads with lengths below 25 bases are discarded. This threshold was selected to optimize the analysis of highly fragmented DNA from ancient archaeological or forensic samples; yet, these parameter can be tuned to specific needs.

Moreover, TRACESPipe uses MAGNET [17] to remove reads from the PhiX control below a certain threshold of similarity. In TRACESPipe, MAGNET runs with a mixture of three Markovian chain models.

Database

High-quality and diverse viral databases increase the accuracy of reference-based assembly, comparative genomics and authentication in metagenomics. TRACESPipe uses four approaches to create and maintain its own database. The default approach downloads automatically all viral sequences from the nucleotide NCBI database into a multi-FASTA using GTO [18] and Entrez [19] through the accession codes. The second approach downloads NCBI (only) references using the same process. The third approach enables adding at any moment a new genome using the accession code or a Fasta file, while the fourth permits to add multiple genomes from a file containing accession codes.

Upon reconstruction of assembled viral sequences, the user has the possibility to add them to the TRACES database (using the third approach), to increase the diversity and quality of the database.

TRACESPipe core

The TRACESPipe core assumes that all the previous steps were taken, i.e. the data preparation and database building. The

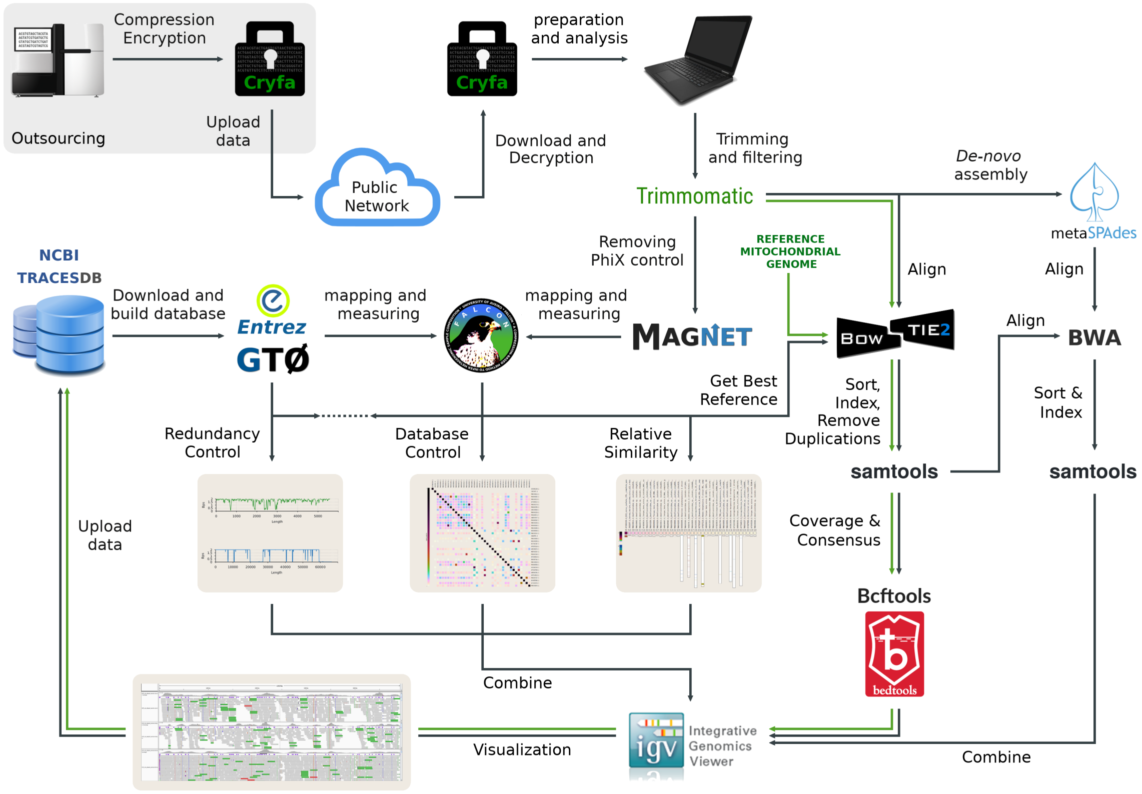

Figure 1. The architecture of TRACESPipe for identification and reconstruction of viral and human mitogenomes at multi-organ level. The tools are represented with the respective logos and names. The green link stands for mitogenomes while the dark for the viral flowline.

data analysis combines three modalities:

- compression-based prediction;
- sequence alignment;
- *de-novo* assembly.

The final output is a hybrid approach that merges the viral genome reconstructions derived from these methods.

Compression-based prediction

The alignment of FASTQ reads (e.g. from a Novaseq run) to each of the sequences of the NCBI viral database (around 200.000) would take months (assuming parallelization). The same task becomes almost unfeasible when analysing multiple FASTQ reads from different organs (it would take years). Therefore, an ultra-fast method that identifies and aligns only the most representative references in the reads is essential.

To scan the reads with highest similarity to the reference database, we use FALCON-meta [9], an alignment-free tool [20]. This tool loads the reads into several Markov, and Tolerant Markov models [21] under relative compression and, then, it freezes those models. Subsequently, it uses context mixing for similarity estimation. In-built in this method is the flexibility to account for SNPs, polymorphisms and structural variants. The final output is a score representing the similarity of the reads to a reference sequence. The highest similarity values for different categories of viruses are then filtered by name and size, where the highest value stands for the best reference.

Alignments to the best reference

After assignment of the best reference by FALCON-meta, the reads are aligned using Bowtie2 [10] with sensitive parameters. Extreme-sensitive parameters can also be applied although at the cost of substantial computational time.

Subsequently, consensus sequences are built with BCFtools [11] using protocols with specific filters to handle SNPs and indexing support from Tabix [22]. Bases with low quality are assigned as N. The variants are stored in BED files using BEDtools [23].

De-novo assembly

The *de-novo* assembly takes place in the pipeline after trimming (data preparation) and serves the purpose of validation of the consensus sequences product of the reference based alignments. Important features of this approach include the possibility to complement the viral genome when the reference is only partial or incomplete, the inclusion of reads that otherwise would be excluded due to high divergence to the reference and the possibility to discover new genomes. TRACESPipe uses the core meta assembler of metaSPAdes [12]. This assembler uses an iterative approach to implement a multisized de Bruijn graph algorithm with multiple k-mer sizes. The output of metaSPAdes, besides multiple channels of information (such as coverage), is a multi-FASTA file with scaffolds.

Hybrid reconstruction

Hybrid methodological approaches in genome assembly, i.e. reference-based combined with *de-novo* assembly provide higher sensitivity and resolution. When the reads are similar to a reference genome, the reference-based approach adds substantially more breadth and depth coverage than *de-novo* assembly, specially at the tips of the scaffolds or contigs. On the other hand, for novel regions or higher concentration of SNPs (or other variations), *de-novo* assembly provides complementary information in the absence of aligned reads.

At this point, all the scaffolds resulting from *de-novo* assembly are aligned to the best reference using BWA [24]. These are then combined with the reference-based alignments and consensus sequence in IGV [13]. The final reconstruction is supervised and validated by human inspection.

Data controls

The pipeline includes three main controls:

- redundancy control;
- database control;
- exogenous control.

These controls are essential to detect the source of abnormal patterns, (i.e. high depth (D) with low breadth (B) coverage), excessive number of flagged genomes in the samples, and presence of exogenous genomes.

Redundancy control

Redundancy control is a way to estimate duplications or low complexity sequences. Repetitive elements on the reference genomes may be over-represented by the same reads, in a fashion such as that, if two regions are very similar, the reads will map to both, creating double of the depth coverage. The clustering of reads around specific areas can also be caused by PCR duplicates and sister duplications, in which cases very high depth yet low breadth coverage may be seen.

These phenomena can be minimized by sequencing the flanking regions with longer reads (e.g. with a PacBio sequencer), normalization at computational level, or inspection of known repetitive or low complexity regions together with the depth and breadth coverage profiles. We chose the latter since besides being very precise and low-cost, it is possible to crosscheck the information with similar sub-regions of exogenous content that might be present in the samples.

We use GTO [18] to identify regions of low complexity [25]. It includes a DNA compressor that estimates the content along each genome. We then cross this information with the coverage profiles generated with BEDTools [23] as well as with the data from the exogenous control. For an example of the redundancy control, see Figure 2.

Additionally, TRACESPipe uses an optional mode to remove duplications in a traditional way, i.e. using the markdup function from Samtools [26]. When using this option, the alignments will not include reads that have been classified as duplications instead of only marking them.

Database control

The database includes viruses that share high similarity either between family members (e.g. *Parvoviridae*) or to the human host (e.g. *Herpesviridae*). This may result in low-level alignments of the reads to the best reference. In order to mitigate this, we apply FALCON-meta technology to measure the cross similarity between the best references. For this purpose, TRACESPipe uses a threshold to analyse only the 40 genomes with the highest similarity scores. This strategy enables us to save substantial computational time while maintaining sufficient precision.

Exogenous control

Exogenous content, i.e. by fungi, bacteria or plants, may display low levels of similarity to the viral or mitochondrial genomes (mitogenomes) [27]. Thus, as a control, TRACESPipe estimates the exogenous sequences content with FALCON-meta [9] using databases for each respective type. The download and construction of the reference databases are automatically driven by the pipeline using GTO and Entrez [19]. The most representative genomes can be aligned according to the reference for further consensus sequence construction and analysis.

Additional features

To assist in the interpretation and analysis of the viral findings, TRACESPipe includes the analysis of human mtDNA. The reads are aligned exclusively to the revised Cambridge Reference sequence (rCRS) [28, 29] using Bowtie2 [10] and a consensus sequence is generated with Bcftools [11].

Also to control for contamination, TRACESPipe has incorporated the quantification of Y-chromosome levels through compression-based predictors [9]. The human Y-chromosome reference is compressed relative to the FASTQ reads and subsequently normalized by size in a logarithmic scale. This computation outputs a value between zero and one, where values near one stand for absence, and near zero full presence. Additional alignments, consensus sequences, and coverage outputs for Y-chromosome are available.

Moreover, TRACESPipe has in-built mapDamage2 [30] for estimation of DNA damage patterns, i.e. the degree of specific alterations in the tips of the reads. This feature is particularly important in the authentication of ancient DNA.

The pipeline also includes a feature to enable specific alignments using automatic search. These alignments can be made according to a sequence identifier or specific pattern name contained in the database (by a FASTA header pattern). For each match, consensus sequences, variant call files and coverage profiles are available.

Additional output breadth and depth coverage tables (2 dimensional matrix with organ as horizontal and viruses as vertical variables), relative similarity results for each organ, and others can be automatically sent by email (requires email configuration).

Finally, there are performance settings, including the specification of the number of threads to be used by the tools. By default, the pipeline calculates and runs with the maximum number of threads available in the system.

Tools

A compilation of the tools integrated into TRACESPipe with the respective home page and reference is available in Table 1. The installation of these tools is fully automated and provided through Conda using a combination of the channels Bioconda [31] and Cobilab (https://github.com/cobilab).

Name	URL	REF
Bcftools	www.htslib.org/doc/bcftools.html	[11]
BEDTools	bedtools.readthedocs.io	[23]
Bowtie2	bowtie-bio.sourceforge.net/bowtie2	[10]
BWA	bio-bwa.sourceforge.net/	[24]
Cryfa	github.com/cobilab/cryfa	[8]
Entrez	www.ncbi.nlm.nih.gov/genome	[19]
FALCON-meta	github.com/cobilab/falcon	[9]
GTO	bioinformatics.ua.pt/gto	[18]
IGV	software.broadinstitute.org/software/igv	[13]
MAGNET	github.com/cobilab/magnet	[17]
mapDamage2	ginolhac.github.io/mapDamage	[30]
metaSPAdes	cab.spbu.ru/software/meta-spades	[12]
Samtools	samtools.sourceforge.net	[26]
Tabix	htslib.org/doc/tabix.html	[22]
Trimmomatic	www.usadellab.org/cms/?page=trimmomatic	[16]

Table 1. Tools integrated into the TRACESPipe with the respective name, home page (URL), and reference (REF).

	Blood			Bone			Brain			Hair			Heart			Kidney			Liver			Lung			Skin			Teeth		
SEQ	F	D	H	F	D	H	F	D	H	F	D	H	F	D	H	F	D	H	F	D	H	F	D	H	F	D	H	F	D	H
B19V	✓	40	100	✓	30	99	✓	10	100	✗	–	–	✓	20	80	✓	20	100	✗	–	–	✗	–	–	✓	25	100	✓	30	95
	✓	48.3	100	✓	36.0	100	✓	12.4	100	✗	0.0	0	✓	0.1	2.8	✓	24.9	100	✗	0.0	0	✗	0.0	0	✓	28.2	100	✓	29.6	100
HHV2	✓	40	100	✗	–	–	✗	–	–	✗	–	–	✗	–	–	✓	20	100	✗	–	–	✗	–	–	✗	–	–	✓	30	100
	✓	58.3	100	✗	0.0	0	✗	0.0	0	✗	0.0	0	✗	0.0	0	✓	28.6	100	✗	0.0	0	✗	0.0	0	✗	0.0	0	✓	43.4	100
HHV3	✓	40	100	✗	–	–	✗	–	–	✗	–	–	✗	–	–	✗	–	–	✗	–	–	✗	–	–	✓	25	100	✗	–	–
	✓	45.3	100	✗	0.0	0	✗	0.0	0	✗	0.0	0	✗	0.0	0	✗	0.0	0	✗	0.0	0	✗	0.0	0	✓	28.8	100	✗	0.0	0
HHV4	✗	–	–	✗	–	–	✓	10	100	✓	5	100	✗	–	–	✗	–	–	✓	20	99	✓	10	99	✗	–	–	✗	–	–
	✗	0.0	0	✗	0.0	0	✓	51.1	100	✓	24.4	97	✗	0.0	0	✗	0.0	0	✓	92.7	100	✓	51.8	100	✗	0.0	0	✗	0.0	0
HHV8	✓	40	100	✗	–	–	✗	–	–	✗	–	–	✗	–	–	✗	–	–	✗	–	–	✗	–	–	✗	–	–	✗	–	–
	✓	105.2	100	✗	0.0	0	✗	0.0	0	✗	0.0	0	✗	0.0	0	✗	0.0	0	✗	0.0	0	✗	0.0	0	✗	0.0	0	✗	0.0	0
HPV	✗	–	–	✗	–	–	✗	–	–	✓	5	90	✓	20	90	✗	–	–	✓	20	100	✗	–	–	✗	–	–	✗	–	–
	✗	0.0	0	✗	0.0	0	✗	0.0	0	✓	2.5	78	✓	9.1	92	✗	0.0	0	✓	19.9	100	✗	0.0	0	✗	0.0	0	✗	0.0	0
TTV	✗	–	–	✓	30	90	✗	–	–	✗	–	–	✗	–	–	✓	20	85	✗	–	–	✗	–	–	✓	25	100	✓	30	100
	✗	0.0	0	✓	13.8	83	✗	0.0	0	✗	0.0	0	✗	0.0	0	✓	1.6	27	✗	0.0	0	✗	0.0	0	✓	24.2	97	✓	29.1	100
VARV	✓	40	100	✗	–	–	✓	10	100	✗	–	–	✗	–	–	✗	–	–	✓	20	100	✓	10	100	✗	–	–	✗	–	–
	✓	43.7	100	✗	0.0	0	✓	10.9	100	✗	0.0	0	✗	0.0	0	✗	0.0	0	✓	22.5	100	✓	11.1	100	✗	0.0	0	✗	0.0	0
MT	✓	40	100	✓	30	100	✓	10	99	✓	5	100	✓	20	100	✓	20	100	✓	20	100	✓	10	100	✓	25	100	✓	30	95
	✓	39.2	100	✓	29.5	100	✓	9.8	99	✓	4.9	96	✓	19.7	100	✓	19.7	100	✓	19.7	100	✓	9.8	99	✓	24.6	100	✓	27.4	99

Table 2. Benchmark of TRACESPipe (depth and breadth coverage) in viral and mitogenomes from 10 different organs. The grey background is the statistical ground truth (simulation conditions), while the white background represents the TRACESPipe output. The F stands for the existence or not of the respective virus in the organ sample, where the ✓stands for viral or mitochondrial genome detection in the sample, while ✗ for the opposite. The D stands for the depth coverage and H for the breadth coverage. To replicate use script Benchmark.sh from the repository.

Analyses

We tested the performance of TRACESPipe in analysis of synthetic and real data. The synthetic data were generated using viral and mitogenomes to which specific additional exogenous content and mutation rates had been applied. The ex-vivo data includes DNA sequences from different organs collected in connection to postmortem investigations.

Synthetic Data

We used datasets from several viruses and mitogenomes and simulated the sequencing with ART [32]. The latter was configured to mimic reads from Illumina HiSeq 2500, paired-end data, and read length of 150. The fragmentation was set at 200, while the deviation at 10. The mutation rate, i.e. the simulation of specific SNP percentages, was set using the GTO toolkit. The characteristics of previous conditions are described in the grey-background lines of Table 2.

The blank-background lines of Table 2 present the identification and coverage (depth and breadth) results of TRACESPipe after reconstruction of the synthetic reads from multiple organs. In some of the viruses and mitogenomes, maximum synthetic mutation was set at 20% which means that there are, on average, 20 SNPs for every 100 bases. The whole experiment took less than 90 minutes on a laptop computer.

When assessing the individual depth coverage values (D), we found, in some cases, values higher than the simulation coverage. These were given by similarity between different regions, as we opted not to normalize the coverage or to apply any equivalent method, but instead to use complexity analysis after duplication removal. Accordingly, we crossed the complexity profiles with the coverage profiles. In the tips of this genome were distinguishable two areas of high depth coverage that correspond to the B19V hairpins and were identified as low by our complexity analysis. An example of this analysis can be seen in Figure 2, with the identified B19V DNA in a blood sample.

As shown in Table 2, all the viral and mitogenomes from the samples were identified and reconstructed (without false positives). A FASTA sequence for each genome was generated along with the necessary controls. This proof of concept shows the efficiency of TRACESPipe in the identification and reconstruction of viral and human mitochondrial genomes at multi-organ level even when high mutation rates in relation to a reference exist.

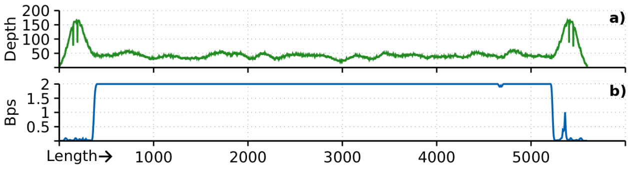

Figure 2. Redundancy controls with coverage (a) and complexity (b) profiles for a B19V DNA sequence identified in a blood sample. Depth stands for depth coverage while Bps for Bits per base. Lower values of Bps mean higher redundancy. The length scale in nucleotides.

Real Data

We tested the performance of TRACESPipe in the identification of viral DNA reads derived from different tissues of a recently deceased individual. The organs analyzed were bone, bone marrow, brain, heart, kidney, liver, lung, blood and skin. Each sample was processed individually in the laboratory prior to sequencing in Illumina Novaseq with 150 paired-end reads. After de-multiplexing, the sequenced reads were split according to the organs of origin. TRACESPipe identified human parvovirus B19 (ssDNA), JC polyomavirus (dsDNA), and the human mitogenome. The percentages of breadth coverage of the mapped reads against the best reference for each organ sample are depicted in Figure 3. The alignments of the reads for JCPyV and the human mitogenome in selected organs can be seen in Figure 4.

A Blast [33] search of the generated consensus sequences of JCPyV from kidney and liver showed an identity of 99% (some small regions without reads mapped). All the SNPs were congruent between organ samples with high coverage. In the skin sample, the number of reads that aligned to the reference were insufficient; yet, identical SNPs in relation to the reference were also detected. This pattern was also similar for B19V as

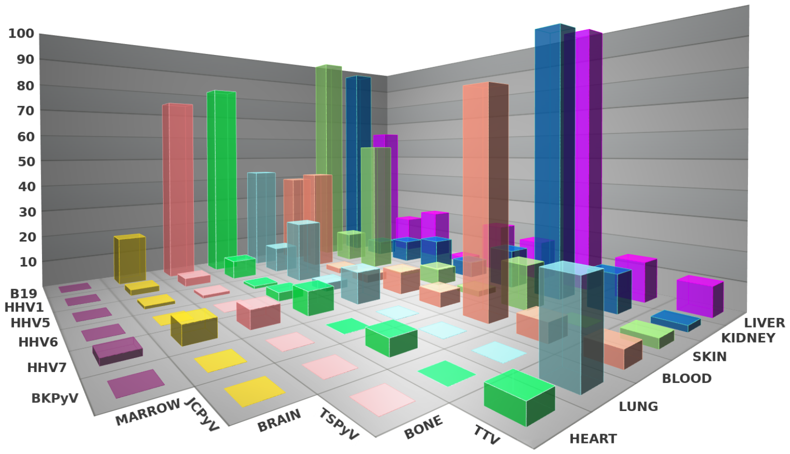

Figure 3. Breadth coverage percentage (z-axis) of the (real) mapped reads against the best reference virus for each organ sample. The plot is restricted to viral types with a minimum similarity of 10% in at least one of the organs. The bottom corner had shallow values, which given space constraints were not included.

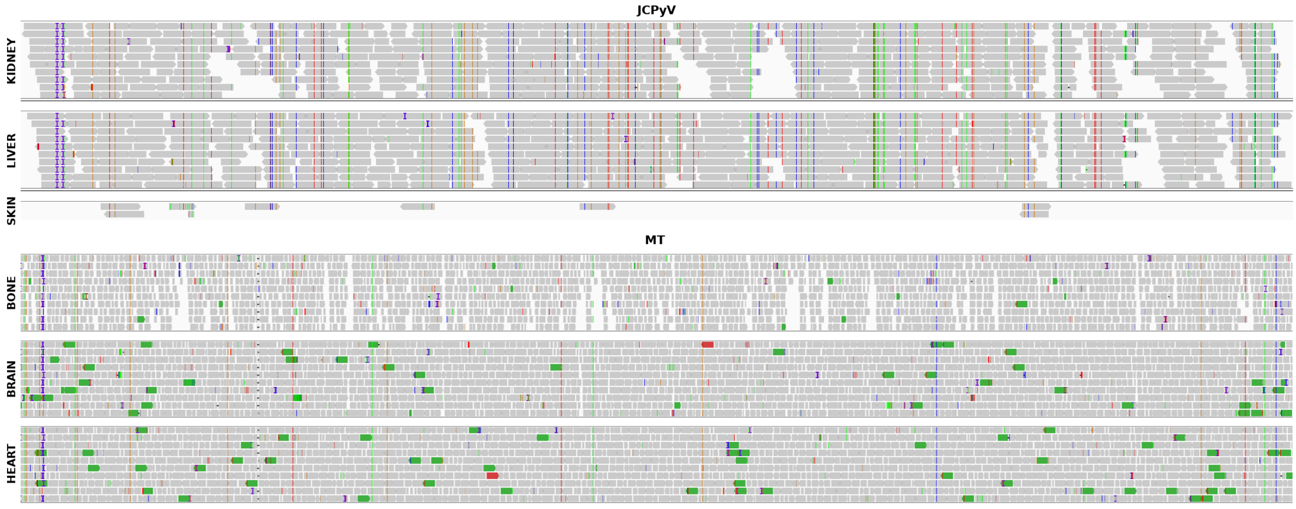

Figure 4. Visualization of the read alignments of each organ sample for JCPyV and human mitogenome (MT). The maps have been adapted from the IGV output after TRACESPipe computation. Each map shows the complete alignments in the respective scale. The JCPyV maps include removal of duplications while the MT maps do contain the duplications as an example of TRACESPipe being able to filter or maintain these duplications (reads highlighted green by the IGV show possible duplications). Vertical line stand for SNPs. Red reads stand for an inferred insert size that is larger than expected (possible evidence of a deletion).

well as the mitogenome, suggesting that the within-host variation of the viral genomes in different organs is minimal. This conclusion shows the importance of this pipeline to close the gap of combining the information at multi-organ level.

The genomes of B19V, JCPyV and human mitogenome were fully assembled with high coverage, namely >25x, >40x, and >80x, respectively. These genomes were uploaded into the TRACES database and deposited in the Nucleotide Archive with the accession names: NC_NUMBER1, NC_NUMBER2, NC_NUMBER3 (submitted).

Conclusions

TRACESPipe is an automatic and efficient pipeline for the reconstruction and analysis of viral genomes. It profits from the synergy between reference-based and -free approaches to rise the quality and certainty of prediction to a high level. Indeed, the pipeline performed outstandingly in assignment and reconstruction of viral genomes even when high mutation rates were simulated.

As a unique feature, it supports the merging of data from multiple organ samples. This gives an advantage in relation to existing tools by permitting the evaluation of intra-host genomic diversity. In terms of the viral populations persisting in the body, this opens the way for the investigation of a diverse range of topics, such as viral tissue tropism, evolution, fitness and disease associations.

Moreover, the seemingly extremely low within-host variability of viral genomes [34] and human mitogenomes in different organs may signify and advantage for efficient and complete genome assembly. In fact, the quality of data could be significantly improved by merging complementary sequencing reads between organs towards a robust sequence genome. This may be particularly useful in the scenario of highly fragmented DNA samples, with genomic regions missing, degraded or with high-degree damage, as is frequently the case of ancient DNA.

Another special component of TRACESPipe is the analysis of the mtDNA. Besides serving as a control for external contamination, the cross association of the viral types with the geographical distribution of this marker can be extremely valuable in epidemiological or archaeovirological studies as well as in forensic investigations, to evaluate the origins of unidentified individuals [35, 36].

Additional features such as encryption and numerous quality and contamination controls make of TRACESPipe a robust tool for comprehensive analysis of genomic data.

Availability of source code and requirements

Lists the following:

- Project name: TRACES Pipeline
- Home page: https://github.com/viromelab/tracespipe
- Operating system(s): Linux / Unix
- Programming language: Shell
- Other requirements: Conda
- License: GNU GPL3.

Availability of supporting data and materials

Supporting data and an archival copy of the code are available via the GigaScience repository GigaDB. Additional file Supplementary information: Supplementary Methods and Results are available via the additional file associated with this article

Additional File

Supplementary information: Supplementary File is available via the additional file associated with this article.

Declarations

List of abbreviations

BWA: Burrows Wheeler Aligner; B19V: Human parvovirus B19; DNA: Deoxyribonucleic acid; dsDNA: double stranded Deoxyribonucleic acid; GPL: GNU Public License; HPV: Human Papillomavirus; HHV: Human Herpesvirus; JCPyV: JC polyomavirus; MT: mitogenome; NCBI: National Center for Biotechnology Information; NGS: Next-generation sequencing; SNP: Single Nucleotide Polymorphism; ssDNA: single stranded deoxyribonucleic acid; TTV: torque teno virus; VARV: variola virus; VCF: variant call format;

Ethical Approval

The study using tissues from autopsies performed at the Department of Forensic Medicine of Helsinki University was reviewed by the Ethics Committee of the Helsinki and Uusimaa Hospital district, dosier number : 164/13/03/00/114.

Competing Interests

The authors declare that they have no competing interests.

Funding

This work was partially funded by FEDER (Programa Operacional Factores de Competitividade-COMPETE), Portuguese National Funds through the FCT-Foundation for Science and Technology (in the context of the projects UID/CEC/00127/2014 and PTCD/EEI-SII/6608/2014), Finnish Medical Foundation, Finnish Cultural Foundation, Juhani Aho Foundation for Medical Research, Jane & Aatos Erkko Foundation, Medicinska Understödsföreningen Liv och Hälsa, the Finnish Society of Sciences and Letters and the University of Helsinki Doctoral Programme in Biomedicine.

Author's Contributions

D.P., A.S, and M.P. conceived and designed the experiments; D.P., M.T., L.P. performed the experiments; D.P., M.T., L.P., K.H., A.S., and M.P. analyzed the data; D.P., M.T., L.P., K.H., A.S., and M.P. wrote the paper.

References

1. Simmonds P. Methods for virus classification and the challenge of incorporating metagenomic sequence data. Journal of General Virology 2015;96(6):1193–1206.
2. Li Y, Wang H, Nie K, Zhang C, Zhang Y, Wang J, et al. VIP: an integrated pipeline for metagenomics of virus identification and discovery. Scientific reports 2016;6:23774.
3. Ren J, Ahlgren NA, Lu YY, Fuhrman JA, Sun F. VirFinder: a novel k-mer based tool for identifying viral sequences from assembled metagenomic data. Microbiome 2017;5(1):69.
4. Rampelli S, Soverini M, Turroni S, Quercia S, Biagi E, Brigidi P, et al. ViromeScan: a new tool for metagenomic viral community profiling. BMC genomics 2016;17(1):165.
5. Laffy PW, Wood-Charlson EM, Turaev D, Weynberg KD, Botté ES, van Oppen MJ, et al. HoloVir: a workflow for investigating the diversity and function of viruses in invertebrate holobionts. Frontiers in microbiology 2016;7:822.
6. Tithi SS, Aylward FO, Jensen RV, Zhang L. FastViromeExplorer: a pipeline for virus and phage identification and abundance profiling in metagenomics data. PeerJ 2018;6:e4227.
7. Vilsker M, Moosa Y, Nooij S, Fonseca V, Ghysens Y, Dumon K, et al. Genome Detective: an automated system for virus identification from high-throughput sequencing data. Bioinformatics 2018;35(5):871–873.
8. Hosseini M, Pratas D, Pinho AJ. Cryfa: a secure encryption tool for genomic data. Bioinformatics 2018;35(1):146–148.
9. Pratas D, Hosseini M, Grilo G, Pinho A, Silva R, Caetano T, et al. Metagenomic Composition Analysis of an Ancient Sequenced Polar Bear Jawbone from Svalbard. Genes 2018;9(9):445.
10. Langmead B, Salzberg SL. Fast gapped-read alignment with Bowtie 2. Nature Methods 2012;9(4):357.
11. Li H. A statistical framework for SNP calling, mutation discovery, association mapping and population genetical parameter estimation from sequencing data. Bioinformatics 2011;27(21):2987–2993.
12. Nurk S, Meleshko D, Korobeynikov A, Pevzner PA. metaSPAdes: a new versatile metagenomic assembler. Genome Research 2017;27(5):824–834.
13. Robinson JT, Thorvaldsdóttir H, Winckler W, Guttman M, Lander ES, Getz G, et al. Integrative genomics viewer. Nature biotechnology 2011;29(1):24.
14. Hernaez M, Pavlichin D, Weissman T, Ochoa I. Genomic Data Compression. Annual Review of Biomedical Data Science 2019;2.
15. Kircher M. Analysis of high-throughput ancient DNA sequencing data. In: Ancient DNA Springer; 2012.p. 197–228.
16. Bolger AM, Lohse M, Usadel B. Trimmomatic: a flexible trimmer for Illumina sequence data. Bioinformatics 2014;30(15):2114–2120.
17. Pratas D, Pinho AJ. Metagenomic composition analysis of sedimentary ancient DNA from the Isle of Wight. In: 2018 26th European Signal Processing Conference (EUSIPCO) IEEE; 2018. p. 1177–1181.
18. Almeida JR, Pinho AJ, Oliveira JL, Fajarda O, Pratas D. GTO: a toolkit to unify pipelines in genomic and proteomic research. bioRxiv 2020;.

19. Wheeler DL, Barrett T, Benson DA, Bryant SH, Canese K, Chetvernin V, et al. Database resources of the national center for biotechnology information. Nucleic Acids Research 2006;35(suppl_1):D5–D12.
20. Zielezinski A, Girgis HZ, Bernard G, Leimeister CA, Tang T Kujiand Dencker, Lau AK, et al. Benchmarking of alignment-free sequence comparison methods. BioRxiv 2019;p. 611137.
21. Pratas D, Hosseini M, Pinho AJ. Substitutional Tolerant Markov Models for Relative Compression of DNA Sequences. In: 11th Int. Conf. on Practical Applications of Computational Biology & Bioinformatics Springer; 2017. p. 265–272.
22. Li H. Tabix: fast retrieval of sequence features from generic TAB-delimited files. Bioinformatics 2011;27(5):718–719.
23. Quinlan AR. BEDTools: the Swiss-army tool for genome feature analysis. Current protocols in bioinformatics 2014;47(1):11–12.
24. Li H, Durbin R. Fast and accurate long-read alignment with Burrows–Wheeler transform. Bioinformatics 2010;26(5):589–595.
25. Pinho AJ, Garcia SP, Pratas D, Ferreira PJ. DNA sequences at a glance. PloS one 2013;8(11):e79922.
26. Li H, Handsaker B, Wysoker A, Fennell T, Ruan J, Homer N, et al. The sequence alignment/map format and SAMtools. Bioinformatics 2009;25(16):2078–2079.
27. Budowle B, Connell ND, Bielecka-Oder A, Colwell RR, Corbett CR, Fletcher J, et al. Validation of high throughput sequencing and microbial forensics applications. Investigative genetics 2014;5(1):9.
28. Andrews RM, Kubacka I, Chinnery PF, Lightowlers RN, Turnbull DM, Howell N. Reanalysis and revision of the Cambridge reference sequence for human mitochondrial DNA. Nature genetics 1999;23(2):147.
29. Anderson S, Bankier AT, Barrell BG, de Bruijn MH, Coulson AR, Drouin J, et al. Sequence and organization of the human mitochondrial genome. Nature 1981;290(5806):457–465.
30. Jónsson H, Ginolhac A, Schubert M, Johnson PL, Orlando L. mapDamage2.0: fast approximate Bayesian estimates of ancient DNA damage parameters. Bioinformatics 2013;29(13):1682–1684.
31. Grüning B, Dale R, Sjödin A, Chapman BA, Rowe J, Tomkins-Tinch CH, et al. Bioconda: sustainable and comprehensive software distribution for the life sciences. Nature methods 2018;15(7):475.
32. Huang W, Li L, Myers JR, Marth GT. ART: a next-generation sequencing read simulator. Bioinformatics 2011;28(4):593–594.
33. Ye J, McGinnis S, Madden TL. BLAST: improvements for better sequence analysis. Nucleic acids research 2006;34(2):W6–W9.
34. Norja P, Eis-Hübinger AM, Söderlund-Venermo M, Hedman K, Simmonds P. Rapid sequence change and geographical spread of human parvovirus B19: comparison of B19 virus evolution in acute and persistent infections. Journal of virology 2008;82(13):6427–6433.
35. Toppinen M, Perdomo M, Palo J, Simmonds P, Lycett S, Söderlund-Venermo M, et al. Bones hold the key to DNA virus history and epidemiology. Scientific reports 2015;5:17226.
36. Forni D, Cagliani R, Clerici M, Pozzoli U, Sironi M. You Will Never Walk Alone: Codispersal of JC Polyomavirus with Human Populations. Molecular biology and evolution 2019;.

Click here to access/download
Supplementary Material
main.blg

Manuscript
Click here to download Personal Cover CoverLetter.pdf

Helsinki, January 16th 2020

Dear Editor:

We are pleased to submit the manuscript entitled "**A hybrid pipeline for reconstruction and analysis of viral genomes at multi-organ level**" for consideration for publication in GigaScience as a technical note.

In this work, we describe a novel bioinformatics pipeline for processing and analysis of virus sequencing data <u>from multiple organ samples</u>. This feature allows for the comparison, investigation and analysis of within-host genomic variability, which is currently not supported by existing pipelines. This capability is however critical to study many aspects of virus biology and pathogenesis and thus, it has a wide application potential.

The pipeline described in our paper is likely to be of interest not only to virologist but also to readers of other areas such as forensics and ancient DNA studies, as it includes many other valuable features like mitochondrial DNA, Y chromosome analysis and DNA damage estimation.

We confirm that this work is original and has not been published elsewhere nor is it currently under consideration for publication elsewhere. We have no conflicts of interest to disclose.

Thank you for your consideration of this manuscript.

Sincerely,

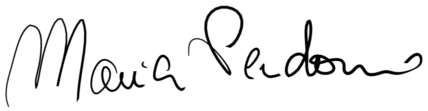

María Fernanda Perdomo
MD PhD
Department of Virology
Faculty of Medicine, University of Helsinki
maria.perdomo@helsinki.fi
+358442036163
