# Supplementary material for: A hybrid pipeline for reconstruction and analysis of viral genomes at multi-organ level
Source: Gigascience. 2020 Aug 20;9(8):giaa086. doi: 10.1093/gigascience/giaa086 (PMC7439602; doi:10.1093/gigascience/giaa086)
Supplement: giaa086_GIGA-D-20-00018_Revision_3 [file giaa086_giga-d-20-00018_revision_3.pdf]

GigaScience

A hybrid pipeline for reconstruction and analysis of viral genomes at multi-organ level

--Manuscript Draft--

**Manuscript Number:**	GIGA-D-20-00018R3
**Full Title:**	A hybrid pipeline for reconstruction and analysis of viral genomes at multi-organ level
**Article Type:**	Technical Note

Funding Information:

Fundação para a Ciência e a Tecnologia (PTCD/EEI-SII/6608/2014)	PhD Diogo Pratas
Fundação para a Ciência e a Tecnologia (UID/CEC/00127/2014)	PhD Diogo Pratas
Suomen Lääketieteen Säätiö (FI)	Not applicable
Jane ja Aatos Erkon Säätiö	Not applicable
Medicinska Understödsföreningen Liv och Hälsa	Not applicable
Suomalainen Tiedeakatemia	Not applicable
Helsingin Yliopiston Tiedesäätiö	Not applicable
Juhani Aho Foundation for Medical Research	Not applicable
Koneen Säätiö	Not applicable
Magnus Ehrnroothin Säätiö	Not applicable

Abstract:

Background: Advances in sequencing technologies have enabled the characterization of multiple microbial and host genomes, opening new frontiers of knowledge while kindling novel applications and research perspectives. Among these, is the investigation of the viral communities residing in the human body and their impact on health and disease. To this end, the study of samples from multiple tissues is critical, yet, the complexity of such analysis calls for a dedicated pipeline. We provide an automatic and efficient pipeline for identification, assembly and analysis of viral genomes, that combines the DNA sequence data from multiple organs. TRACESPipe relies on cooperation between three modalities: compression-based prediction, sequence alignment, and de-novo assembly. The pipeline is ultra-fast and provides, additionally, secure transmission and storage of sensitive data.
Findings: TRACESPipe performed outstandingly when tested on synthetic and ex-vivo datasets, identifying and reconstructing all the viral genomes, including those with high levels of single nucleotide polymorphisms, as well as detecting even minimal levels of genomic variation between different organs.
Conclusions: TRACESPipe introduces the possibility to evaluate within-host variability with its uniqueness to process and analyze simultaneously samples from different sources. This opens up the possibility to investigate viral tissue tropism, evolution, fitness and disease associations. Moreover, additional features such as DNA damage estimation, mitochondrial DNA reconstruction and analysis, and exogenous-source controls expand the utility of this pipeline to other fields such as forensics and ancient DNA studies.
TRACESPipe is released under GPLv3 and is available for free download at https://github.com/viromelab/tracespipe.

**Corresponding Author:**	Diogo Pratas<br><br>PORTUGAL
**Corresponding Author Secondary Information:**	
**Corresponding Author's Institution:**	
**Corresponding Author's Secondary Institution:**	

Powered by Editorial Manager® and ProduXion Manager® from Aries Systems Corporation

**First Author:**	Diogo Pratas
**First Author Secondary Information:**	
**Order of Authors:**	Diogo Pratas
	Mari Toppinen
	Lari Pyöriä
	Klaus Hedman
	Antti Sajantila
	Maria Perdomo
**Order of Authors Secondary Information:**	
**Response to Reviewers:**	Dear Hans,<br><br>Thank you!<br>The tools have been registered.<br>Thank you for spotting the typo in the data availability section.<br>The materials have been submitted to the editorialmanager.<br><br>Best wishes,<br>Diogo
**Additional Information:**	
**Question**	**Response**
Are you submitting this manuscript to a special series or article collection?	No
**Experimental design and statistics**<br><br>Full details of the experimental design and statistical methods used should be given in the Methods section, as detailed in our Minimum Standards Reporting Checklist. Information essential to interpreting the data presented should be made available in the figure legends.<br><br>Have you included all the information requested in your manuscript?	Yes
**Resources**<br><br>A description of all resources used, including antibodies, cell lines, animals and software tools, with enough information to allow them to be uniquely identified, should be included in the Methods section. Authors are strongly encouraged to cite Research Resource Identifiers (RRIDs) for antibodies, model organisms and tools, where possible.	Yes

Have you included the information requested as detailed in our Minimum Standards Reporting Checklist?	
**Availability of data and materials**<br><br>All datasets and code on which the conclusions of the paper rely must be either included in your submission or deposited in publicly available repositories (where available and ethically appropriate), referencing such data using a unique identifier in the references and in the "Availability of Data and Materials" section of your manuscript.<br><br>Have you have met the above requirement as detailed in our Minimum Standards Reporting Checklist?	Yes

Click here to access/download;LaTeX - Main Document (TeX file);main.tex

GigaScience, 2020, 1–10

doi: xx.xxxx/xxxx
Manuscript in Preparation
TECHNICAL NOTE

TECHNICAL NOTE

A hybrid pipeline for reconstruction and analysis of viral genomes at multi-organ level

Diogo Pratas[1,4,5,*], Mari Toppinen[1], Lari Pyöriä[1], Klaus Hedman[1,3], Antti Sajantila[2,‡] and Maria F. Perdomo[1,*]

[1]Department of Virology, University of Helsinki, Finland and [2]Department of Forensic Medicine, University of Helsinki, Finland and [3]HUSLAB, Helsinki University Hospital, Finland and [4]Department of Electronics, Telecommunications and Informatics, University of Aveiro, Portugal and [5]Institute of Electronics and Informatics Engineering of Aveiro, University of Aveiro, Portugal

[*]pratas@ua.pt; maria.perdomo@helsinki.fi
‡ senior author

Abstract

Background: Advances in sequencing technologies have enabled the characterization of multiple microbial and host genomes, opening new frontiers of knowledge while kindling novel applications and research perspectives. Among these, is the investigation of the viral communities residing in the human body and their impact on health and disease. To this end, the study of samples from multiple tissues is critical, yet, the complexity of such analysis calls for a dedicated pipeline. We provide an automatic and efficient pipeline for identification, assembly and analysis of viral genomes, that combines the DNA sequence data from multiple organs. *TRACESPipe* relies on cooperation between three modalities: compression-based prediction, sequence alignment, and *de-novo* assembly. The pipeline is ultra-fast and provides, additionally, secure transmission and storage of sensitive data. **Findings:** TRACESPipe performed outstandingly when tested on synthetic and *ex-vivo* datasets, identifying and reconstructing all the viral genomes, including those with high levels of single nucleotide polymorphisms. It also detected minimal levels of genomic variation between different organs. **Conclusions:** TRACESPipe's uniqueness to process and analyze simultaneously samples from different sources, enables the evaluation of within-host variability. This opens up the possibility to investigate viral tissue tropism, evolution, fitness and disease associations. Moreover, additional features such as DNA damage estimation, mitochondrial DNA reconstruction and analysis, as well as exogenous-source controls expand the utility of this pipeline to other fields such as forensics and ancient DNA studies. TRACESPipe is released under GPLv3 and is available for free download at https://github.com/viromelab/tracespipe.

Key words: efficient pipeline; multi-organ sequencing; viral genomes; genome analysis; parvovirus B19; JC polyomavirus; mitochondrial DNA

Introduction

The field of virology has experienced a revolution along with the introduction of next generation sequencing technologies (NGS) as the number of emerging and newly discovered viruses continues to rise at near exponential rates. Advantages of NGS over traditional methods include multiplex capability, analytical resolution and unbiased exploration of microbial metagenomic composition. Thanks to NGS, long standing questions on the virome and on its interactions with the host can now be investigated. These include the study of the types and genetic diversities of the viral populations residing in different organs of the human body [1]. To this end, the examination of samples from multiple tissues of an individual is essential, yet, the integration and analysis of such data has a high degree of complexity.

Compiled on: July 22, 2020.
Draft manuscript prepared by the author.

Along with its unquestionable impact, NGS has also brought up new challenges due to the volume of data derived. This has rendered necessary the design of automatic workflows, or pipelines, that use high-level algorithms to connect multiple instructions and tools in unique and custom-based architectures. Building a pipeline is far from trivial as multiple factors need to be taken into account, such as sequencing technologies, biological targets, research aims, compatibility between tools, databases and computational resources.

For processing of virus sequencing data, several pipelines exist (e.g. VIP [2], VirFinder [3], ViromeScan [4], HoloVir [5], iVirus [6], VirMAP [7], FastViromeExplorer [8], and GenomeDetective [9]). However, these tools are not optimized for the analysis of data derived from multiple organs, leaving each tissue to be analysed individually and independently, at the expense of much computational time.

In this article, we describe TRACESPipe, the first next-generation sequencing pipeline for identification, analysis and assembly of viral DNA at multi-organ level. For robust mapping, TRACESPipe uses a hybrid approach that combines the results of reference-based and -free methods. Moreover, it includes the analysis of human mitochondrial DNA (mitogenomes), a valuable phylogeographycal marker, to assist in the interpretation of viral findings. Additional features include secure transmission and storage of sensitive data, quality controls, DNA damage estimation and human Y-chromosome analysis.

Methods

TRACESPipe' workflow (Figure 1) begins with encryption using Cryfa [10] to protect sensitive information such as human genomic data. This is a unique feature not commonly embedded in existing pipelines but which is critical when dealing with e.g. clinical or forensic samples. After quality control, the analysis of viral sequences is driven via two parallel approaches: the first one applies initially FALCON-meta [11] to scan the viral reference genomes with highest similarity to the data, followed by alignment of the reads to the identified best references using Bowtie2 [12] and generation of a consensus sequence using BCFtools [13]. The second approach consists of *de-novo* assembly (metaSPAdes [14]) to reconstruct *in silico* viral genomes by building scaffolds from overlapping reads. The alignments and scaffolds derived from each approach are at last combined with a competitive alignment-based approach using BWA [15] and global measures to build a high-quality genome draft. Finally, the multi-organ analysis takes places through a sensitive consensus of the available organ data for each virus. Although the pipeline is completely automatic, the multiple intermediary-alignment phases can be interactively supervised with Integrative Genomics Viewer (IGV) [16].

Figure 1 depicts the architecture of TRACESPipe, where the green line stands for the human mitochondrial flowline. This pipeline has been tested in the analysis of data derived from Illumina HiSeq and NovaSeq platforms. The operating systems required are Linux or Unix. The cygwin [17] can be used as an alternative for Windows operating systems. The installation and configuration procedures, as well as the commands for the runs and structure of the output data are detailed in the Supplementary Section 2 (Reproducibility).

Below we describe the functionalities and options of TRACESPipe, namely data privacy, storage, preparation, and the creation and maintenance of the viral database. Moreover, we describe the TRACESPipe core, the respective controls and additional features.

Data privacy

TRACESPipe provides secure encryption of genomic data using Cryfa [10]. This tool follows industry recommendations for upholding the security of in-transit and at-rest genomic data. Cryfa securely encrypts FASTQ files by a packing transformation after which the information is shuffled and encrypted. The core encryption method uses Advanced Encryption Standard (AES). With this tool TRACESPipe guarantees preservation of the confidentiality, integrity, and authenticity of personal sequencing data.

Data storage

The amount of data resulting from high-throughput sequencing poses a challenge for its immediate and long-term storage. Possible solutions are to discard non-important data, when possible, and/or data compression [18]. The choice of the compressor always comes with a trade-off between compression capacity and/or speed. We opted for relying substantially upon speed.

In TRACESPipe, all temporary data are erased after use, while permanent data are stored using binary file formats (BAM, Bcf) or compressed with lossless approaches. For the data compression, general purpose tools (Gzip and Bzip2) as well as Cryfa [10] are used.

Data preparation

Prior to analysis, the reads need to be trimmed and cleaned from sequence-control genomes (PhiX) and/or reads that are too short, contain sequencing errors or have low quality scores [19].

TRACESPipe uses Trimmomatic [20] to cut the adapter and other Illumina-specific sequences from the reads. Technically, it removes content from an adapters' list having a maximum mismatch that allows a full match of 2. The palindrome and simple clip threshold are set at 30 and 10, respectively. The minimum quality-score required to keep a base at the beginning and at the end are set at 3. Also, it is set to filter low-quality data (sliding window of 4 with an average quality of 15). Reads with lengths below 25 bases are discarded. This threshold was selected to optimize the analysis of highly fragmented DNA from ancient archaeological or forensic samples; yet, these parameter can be tuned to specific needs.

Moreover, TRACESPipe uses MAGNET [21] to remove reads from the PhiX control below a certain threshold of similarity. In TRACESPipe, MAGNET runs with a mixture of three Markov chain models.

Database

High-quality and diverse viral databases increase the accuracy of reference-based assembly, comparative genomics and authentication in metagenomics. TRACESPipe uses four approaches to create and maintain its own database. The default approach downloads automatically all viral sequences from the nucleotide NCBI database into a multi-FASTA using GTO [22] and Entrez [23] through the accession codes. The second approach downloads NCBI (only) references using the same process. The third approach enables adding at any moment a new genome using the accession code or a FASTA file, while the fourth permits to add multiple genomes from a file containing accession codes.

Upon reconstruction of assembled viral sequences, the user has the possibility to add them to the TRACES database (using

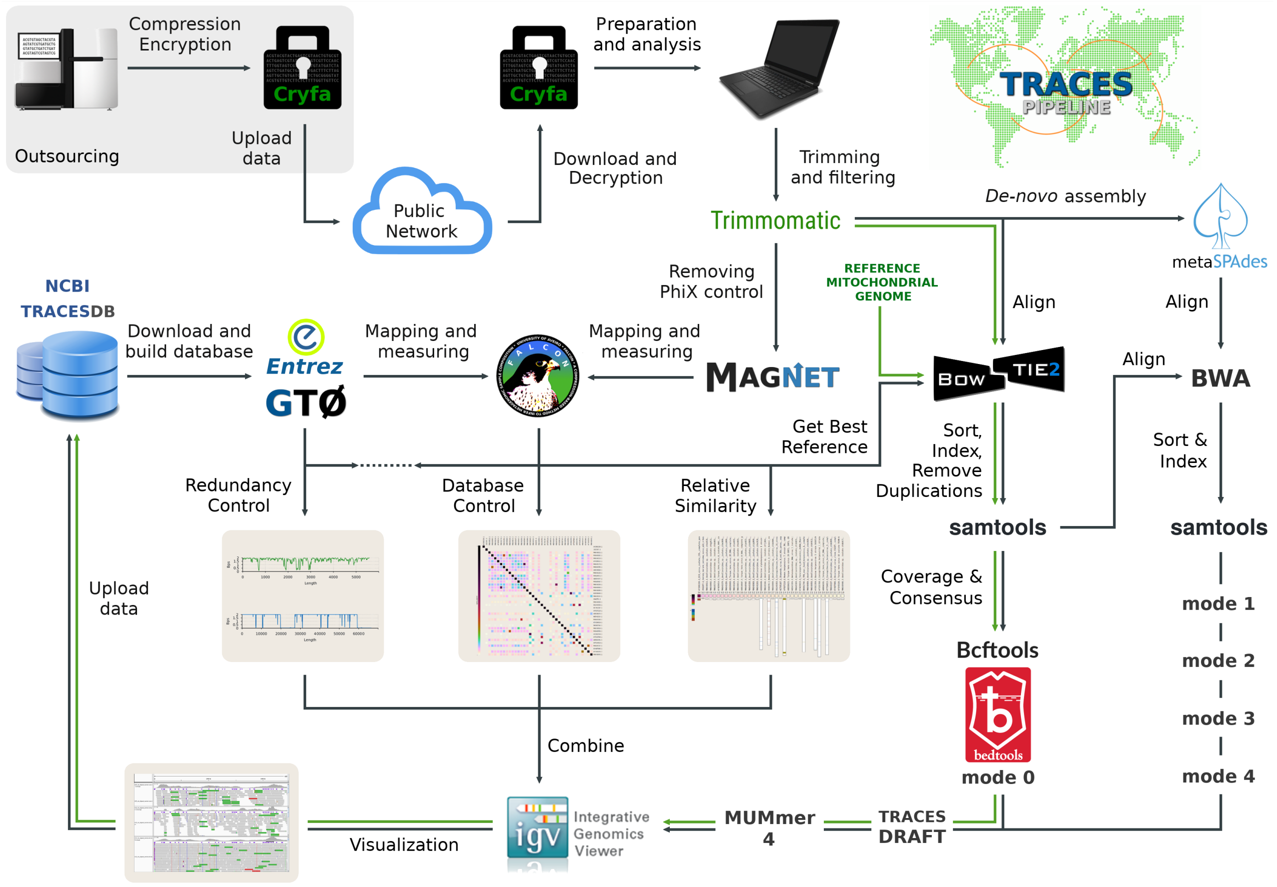

Figure 1. The architecture of TRACESPipe for identification, reconstruction, and analysis of viral and human mitogenomes at multi-organ level. The tools are represented with the respective logos and names. The green link stands for mitogenomes while the dark for the viral flowline.

the third approach), to increase the diversity and quality of the database. For the reconstruction and analysis of non-human viral and mitogenomes, TRACESPipe has also enabled the possibility to manually create the database, select the viruses by host, database type, among other features, using the new NCBI viral interface at [24].

TRACESPipe core

The TRACESPipe core assumes that all the previous steps were taken, i.e. the data preparation and database building. The data analysis combines three modalities:

- compression-based prediction;
- sequence alignment;
- *de-novo* assembly.

The final output is a hybrid approach that merges the viral genome reconstructions derived from these methods.

Compression-based prediction

The alignment of FASTQ reads (e.g. from a Novaseq run) to each of the sequences of the NCBI viral database (around 200.000) would take months (assuming parallelization). The same task becomes almost unfeasible when analysing multiple FASTQ reads from different organs (it would take years). Therefore, an ultra-fast method that identifies and aligns only the most representative references in the reads is essential.

To scan the reads with highest similarity to the reference database, we use FALCON-meta [11], an alignment-free tool [25]. This tool loads the reads into several Markov, and Tolerant Markov models [26] under relative compression and, then, it freezes those models. Subsequently, it uses context mixing for similarity estimation. In-built in this method is the flexibility to account for any polymorphisms or structural variants. The final output is a score representing the similarity of the reads to each reference sequence. The highest similarity values for different categories of viruses are then filtered by name and size, where the highest value stands for the best reference.

TRACESPipe uses FALCON-meta as similarity predictor for single or multiple organs. For the latter, the best reference for each virus is chosen among the most frequent in all the organs.

Alignments to the best reference

After assignment of the best reference by FALCON-meta, the reads are aligned using Bowtie2 [12] with very high sensitive parameters. Extreme-sensitive parameters can also be applied although at substantial cost of computational time. Nevertheless, the analysis can be made with very high sensitive parameters thanks to the selection of a best reference for each virus, instead of whole alignments to each of the existing viral references (linear vs. quadratic complexity).

Subsequently, consensus sequences are built with BCFtools [13] using protocols with specific filters to handle SNPs and indexing support from Tabix [27]. Bases of low quality are designated as N. The variants are stored in BED files using BEDtools [28].

De-novo assembly

The *de-novo* assembly takes place in the pipeline after trimming (data preparation phase) and serves the purposes of validation of the consensus sequences derived from the reference-based alignments and of complementing the viral genome

when the reference is only partial or contains high levels of variation. TRACESPipe uses the core meta assembler of metaSPAdes [14]. This assembler uses an iterative approach to implement a multisized de Bruijn graph algorithm with multiple k-mer sizes. The output of metaSPAdes, besides multiple channels of information (such as coverage), is a multi-FASTA file with scaffolds.

Hybrid reconstruction

Hybrid methodological approaches in genome assembly, i.e. reference-based combined with *de-novo* assembly, provide higher sensitivity and resolution. When the reads are similar to a reference genome, the reference-based approach adds substantially more breadth and depth coverage than the *de-novo* assembly, especially at the tips of the scaffolds or contigs. On the other hand, for novel regions or higher concentration of SNPs (or other variations), *de-novo* assembly provides complementary information in the absence of aligned reads.

The viral genomes display high diversity [29] in terms of mutations rates and lengths. Thus, reconstruction methods need to efficiently adapt to deliver precise and accurate results. For this purpose, TRACESPipe automatically runs with five modes. The first (mode 0) reconstructs a genome exclusively with an alignment-based approach to the best reference, as previously described. This mode is ideal when the number of mutations is very low. The second (mode 1) uses the consensus resulting from the alignments and aligns the de-novo scaffolds using BWA [15], while giving priority to the former. This approach is suitable for low to moderate number of mutations. The third (mode 2) is built as mode 1, but the priority is given instead to the de-novo scaffolds. The alignments are produced with very high sensitivity, forcing the output to be more similar to the de-novo, when the consensus from the alignments is ambiguous or contains gaps. The fourth (mode 3) finds the scaffolds from the de-novo assembly with highest similarities, as reported by FALCON-meta [21], and uses it as a candidate genome. This mode is ideal when a high-quality genome exists in the sample but has extremely high mutations rates. The fifth (mode 4), uses the scaffolds from mode 3 as reference, and aligns the consensus sequence created in mode 1. After applying the five modes, TRACESPipe computes the number of bases produced by each mode (that do not contain gaps) and selects the sequence with the highest number of bases.

Although this process is completely automatic, both the alignments and the consensus sequences from all the modes can be visualized in IGV [16]. This way it is possible to detect and compare multi-organ variability, as well as to enable final reconstruction, supervision, and validation by human inspection.

Combining multi-organ data

When the within-host variability of viral genomes is very low, complete genome assemblies can be built by merging the consensus sequences from each of the organs. TRACESPipe combines multi-organ data using two levels. At the first, the pipeline identifies the most frequent reference among all the organs, and forces its use in the analysis. This is essential for human supervision, as well as direct comparison of the data. The latter is then combined at the second level.

After viral reconstruction of each organ, zero coverage regions can be combined with others of higher depth, from other organs. Hence, an improved and complete genome can be assembled using multiple alignments with very high sensitivity parameters in BWA [15]. Specifically, TRACESPipe enables to produce a consensus of the multi-organ reconstructed data automatically. This feature can be particularly useful in ancient-DNA studies, in which the DNA is frequently fragmented and has a high degree of damage.

Data controls

The pipeline includes three main controls:

- redundancy control;
- database control;
- exogenous control.

These controls are essential to detect the source of abnormal patterns, (i.e. high depth (D) with low breadth (B) coverage), excessive number of flagged genomes in the samples, and presence of exogenous genomes.

Redundancy control

Redundancy control is a way to estimate duplications or low-complexty regions in the sequences. Repetitive elements on the reference genomes may be over-represented by the same reads. Thus, if two regions are very similar, the reads will map to both, creating double the depth coverage. This phenomenon can also be caused by PCR duplicates and sister duplications, in which cases very-high-depth yet low breadth coverage may be seen.

These events can be minimized by sequencing the flanking regions with longer reads (e.g. with a PacBio sequencer), normalization at computational level, or inspection of known repetitive or low-complexity regions together with the depth and breadth coverage profiles. We chose the latter since, besides being very precise and low-cost, it is possible to cross-check the information with similar sub-regions of exogenous content that might be present in the samples.

We use GTO [22] to identify regions of low complexity [30]. It includes a DNA compressor that estimates the content along each genome. We then cross this information with the coverage profiles generated with BEDTools [28] as well as the data from the exogenous control. TRACESPipe includes the possibility to generate coverage profiles, where the depth scale can be set according to a specific value (normalization) for visualization purposes. For an example of the redundancy control, see Figure 3.

Additionally, TRACESPipe uses an optional mode to remove duplications in a traditional way, i.e. using the markdup function from Samtools [31]. When using this option, the alignments will not include reads that have been classified as duplications, instead of only marking them.

Database control

The database includes viruses that share high similarity to other family members (e.g., *Polyomaviridae*) or to the human host (e.g., *Herpesviridae*). The former may result in high-level mapping of the reads to various references. When the references are full genomes, the mapping automatically finds the best reference; however, when partial genomes are also included, the best reference may be attributed to a partial genome in which only conserved regions are present. In order to mitigate this, we apply FALCON-meta to measure the cross-similarity between the best references. By default, TRACESPipe uses a threshold of 40 genomes scoring the highest similarities. We found this value to be most optimal in terms of computational time and precision. However, it is flexible and can be modified to higher threshold values (up to 100 are still affordable), at the cost of longer computational time.

Regarding the cross-similarity to human DNA, a small number of reads may be assigned to a reference virus albeit of human origin. We apply FALCON-meta to measure and localize regions of high similarity between the viruses and human reference genome.

Exogenous control
Exogenous content, i.e. by fungi, bacteria or plants, may display low levels of similarity to the viral or mitogenomes [32]. Thus, as a control, TRACESPipe estimates the content of exogenous sequences with FALCON-meta [11] using databases for each respective type. The download and construction of the reference databases are automatically driven by the pipeline using GTO [22] and Entrez [23]. The most representative genomes can be aligned according to the reference, for further consensus sequence construction and analysis.

Additional features

To assist in the interpretation and analysis of the viral findings, TRACESPipe includes the analysis of human mitogenomes. The reads are aligned exclusively to the revised Cambridge Reference sequence (rCRS) [33, 34] using Bowtie2 [12], and a consensus sequence is generated with Bcftools [13]. Although the human-mitochondrial reference is used by default, TRACESPipe permits the setting of any reference using the genome identifier. Thus, our pipeline is also flexible for the analysis of viruses in other host species.

Also, to control for contamination, TRACESPipe quantifies the Y-chromosome levels through compression-based predictors [11]. The human Y-chromosome reference is compressed relative to the FASTQ reads and subsequently normalized by size in a logarithmic scale. This computation outputs a value between zero and one, where values near one stand for absence, and near zero for full presence. Additional alignments, consensus sequences, and coverage outputs for Y-chromosome are available.

Moreover, TRACESPipe has in-built mapDamage2 [35] for estimation of DNA damage patterns, i.e. the degree of specific alterations in the tips of the reads. This feature is particularly important in the authentication of ancient DNA.

The pipeline also includes a feature to enable specific alignments using automatic search. These alignments can be made according to a sequence identifier or specific pattern name contained in the database (by a FASTA header pattern). For each match, consensus sequences, variant call files and coverage profiles are available.

TRACESPipe includes Blastn search [36] to identify the species most likely resembling the query. The database can be consulted locally, through automatic construction, or remotely. One of the applications of Blastn is the identification of the scaffolds derived from de-novo assembly, which do not match to any viral or human DNA. This search also enables the finding of potential candidates for novel viruses.

Additional output breadth and depth coverage tables (2 dimensional matrix with organ as horizontal and viruses as vertical variables), relative similarity results for each organ, and others can be automatically sent by email (requires email configuration).

TRACESPipe also includes a logging system to record the output provided by each tool as well as debugging messages and system reports, that can be reset at any time.

Finally, there are performance settings, including the specification of the number of threads to be used by the tools. By default, the pipeline calculates and runs with the maximum number of threads available in the system.

Tools

A compilation of the tools integrated into TRACESPipe with the respective home page and reference is available in Table 1. The installation of these tools is fully automated and provided through Conda using a combination of the channels Bioconda [37] and Cobilab [38].

Name	URL	REF
Bcftools	www.htslib.org/doc/bcftools.html	[13]
BEDTools	bedtools.readthedocs.io	[28]
Blastn	https://blast.ncbi.nlm.nih.gov/	[36]
Bowtie2	bowtie-bio.sourceforge.net/bowtie2	[12]
BWA	bio-bwa.sourceforge.net/	[15]
Cryfa	github.com/cobilab/cryfa	[10]
Entrez	www.ncbi.nlm.nih.gov/genome	[23]
FALCON-meta	github.com/cobilab/falcon	[11]
GTO	bioinformatics.ua.pt/gto	[22]
IGV	software.broadinstitute.org/software/igv	[16]
MAGNET	github.com/cobilab/magnet	[21]
mapDamage2	ginolhac.github.io/mapDamage	[35]
metaSPAdes	cab.spbu.ru/software/meta-spades	[14]
MUMmer4	https://mummer4.github.io/	[39]
Samtools	samtools.sourceforge.net	[31]
Tabix	htslib.org/doc/tabix.html	[27]
Trimmomatic	www.usadellab.org/cms/?page=trimmomatic	[20]

Table 1. Tools integrated into the TRACESPipe with the respective name, home page (URL), and reference (REF).

Analyses

We tested the performance of TRACESPipe in analysis of synthetic and real data. The synthetic data were generated using viral and mitogenomes to which specific additional exogenous content and mutation rates had been applied. The *ex-vivo* data includes DNA sequences from different organs collected in connection to postmortem investigations. The procedure can be replicated using the instructions provided in the Supplementary Material, Reproduciblility section.

Synthetic Data

To test TRACESPipe's efficiency to reconstruct genomes, we created ten datasets containing several reference viruses and mitogenomes with specified mutation rates. Then, we simulated the sequencing process with ART [40], configured to mimic reads from Illumina HiSeq 2500, paired-end data, and read length of 150. The fragmentation was defined at 200, while the deviation at 10. The mutation rate, i.e. the simulation of specific SNP percentages, was set with GTO toolkit [22]. The conditions used are described in the grey-background lines of Table 2. After using TRACESPipe for genome reconstruction, we used dnadiff from the MUMmer4 package [39] to evaluate the identity and number of SNPs between the original and the reconstructed sequences. The breadth and depth coverage of the alignments are described in Supplementary Table 1.

In some of the viruses and mitogenomes, up to 20% synthetic mutations were introduced, representing on average 20 SNPs per 100 bases. The whole experiment, including the automatic reconstruction of all genomes, took approximately 10 minutes on a laptop computer.

As described in the methodology, after trimming and filtering, TRACESPipe proceeds with FALCON-meta [11] to find the best virus references for each organ sample. In Figure 2 (upper map) is represented an example of the output after candidate reference discovery. Here, the candidates were VARV, HHV2, HHV3, HHV8, and B19V with normalized relative similarity (NRS) values higher than 96%, while the remaining were around 2%. The bottom map of Figure 2 shows the similarities

	Blood			Bone			Brain			Hair			Heart			Kidney			Liver			Lung			Skin			Teeth		
SEQ	F	D	S	F	D	S	F	D	S	F	D	S	F	D	S	F	D	S	F	D	S	F	D	S	F	D	S	F	D	S
B19V	✓	40	0	✓	30	1	✓	10	0	✗	–	–	✓	20	20	✓	20	0	✗	–	–	✗	–	–	✓	25	0	✓	30	5
	✓	100	0	✓	100	2	✓	100	0	✗	0.0	0	✓	100	0	✓	100	0	✗	0.0	0	✗	0.0	0	✓	100	0	✓	99.9	5
HHV2	✓	40	0	✗	–	–	✗	–	–	✗	–	–	✗	–	–	✓	20	0	✗	–	–	✗	–	–	✗	–	–	✓	30	0
	✓	100	0	✗	0.0	0	✗	0.0	0	✗	0.0	0	✗	0.0	0	✓	100	0	✗	0.0	0	✗	0.0	0	✗	0.0	0	✓	100	0
HHV3	✓	40	0	✗	–	–	✗	–	–	✗	–	–	✗	–	–	✗	–	–	✗	–	–	✗	–	–	✓	25	0	✗	–	–
	✓	100	0	✗	0.0	0	✗	0.0	0	✗	0.0	0	✗	0.0	0	✗	0.0	0	✗	0.0	0	✗	0.0	0	✓	100	0	✗	0.0	0
HHV4	✗	–	–	✗	–	–	✓	10	0	✓	5	0	✗	–	–	✗	–	–	✓	20	1	✓	10	1	✗	–	–	✗	–	–
	✗	0.0	0	✗	0.0	0	✓	99.9	2	✓	98.8	11	✗	0.0	0	✗	0.0	0	✓	99.9	264	✓	99.8	286	✗	0.0	0	✗	0.0	0
HHV8	✓	40	0	✗	–	–	✗	–	–	✗	–	–	✗	–	–	✗	–	–	✗	–	–	✗	–	–	✗	–	–	✗	–	–
	✓	100	0	✗	0.0	0	✗	0.0	0	✗	0.0	0	✗	0.0	0	✗	0.0	0	✗	0.0	0	✗	0.0	0	✗	0.0	0	✗	0.0	0
HPV	✗	–	–	✗	–	–	✗	–	–	✓	5	10	✓	20	10	✗	–	–	✓	20	0	✗	–	–	✗	–	–	✗	–	–
	✗	0.0	0	✗	0.0	0	✗	0.0	0	✓	98.8	0	✓	100	0	✗	0.0	0	✓	100	0	✗	0.0	0	✗	0.0	0	✗	0.0	0
TTV	✗	–	–	✓	30	10	✗	–	–	✗	–	–	✗	–	–	✓	20	15	✗	–	–	✗	–	–	✓	25	0	✓	30	0
	✗	0.0	0	✓	100	0	✗	0.0	0	✗	0.0	0	✗	0.0	0	✓	100	0	✗	0.0	0	✗	0.0	0	✓	100	0	✓	100	0
VARV	✓	40	0	✗	–	–	✓	10	0	✗	–	–	✓	20	5	✗	–	–	✓	20	0	✓	10	0	✗	–	–	✗	–	–
	✓	100	0	✗	0.0	0	✓	99.9	2	✗	0.0	0	✓	100	21	✗	0.0	0	✓	100	0	✓	99.9	1	✗	0.0	0	✗	0.0	0
MT	✓	40	0	✓	30	0	✓	10	1	✓	5	0	✓	20	0	✓	20	1	✓	20	2	✓	10	0	✓	25	0	✓	30	5
	✓	100	0	✓	100	0	✓	99.5	1	✓	98.8	1	✓	99.9	0	✓	99.9	0	✓	99.9	0	✓	99.5	1	✓	100	0	✓	99.7	0

Table 2. Benchmark of TRACESPipe in viral and mitogenomes assembly from 10 different organs using FASTQ data simulated with different SNPs and coverage rates; simulation using ART and GTO. The grey background is the statistical ground truth (simulation conditions), while the white background represents the evaluation of TRACESPipe output using dnadiff. The F stands for the existence or not of the respective virus in the organ, where the ✓ stands for viral or mitogenome detection in the sample, while ✗ for the opposite. For the simulation conditions (grey background), the D stands for depth coverage and S for the applied percentage of SNPs. For the evaluation (white background), the D stands for the identity and S for the number of SNPs found after full genome reconstruction. The genome sequences (SEQ) were: B19V - parvovirus B19, HHV - human Herpesvirus (multiple types). HPV - human Papillomavirus, TTV - torque teno virus, VARV: variola virus, and MT: human mitogenome. This experiment can be replicated using the script Benchmark.sh.

between candidate pairs. This is critical to detect low level similarities between the respective references. In blood the five genomes were easily detected. Subsequently, the reads were aligned to the best reference for each genome, a consensus sequence created and combined with de-novo assembled scaffolds.

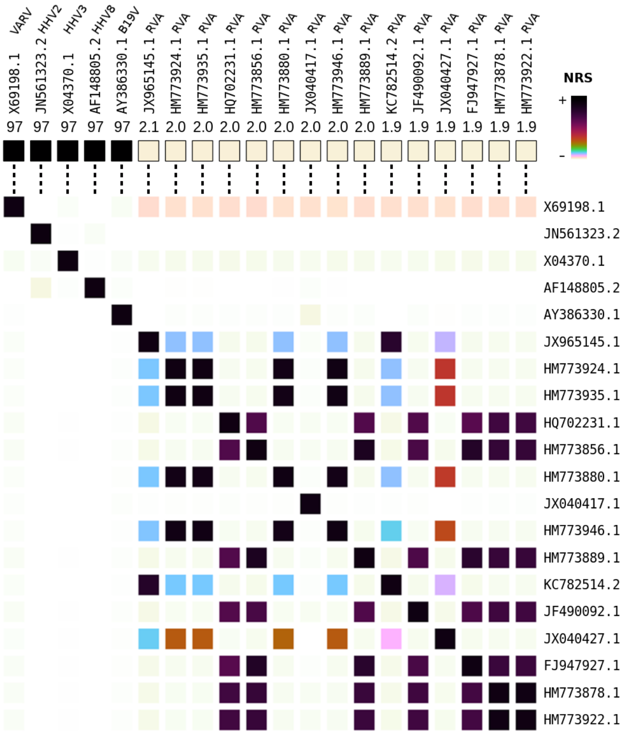

Figure 2. Normalized Relative Similarity (NRS) for the synthetic blood sample provided by TRACESPipe. The upper map depicts the highest NRS values of the reads according to the references and the lower map the cross similarity between each of reference pair.

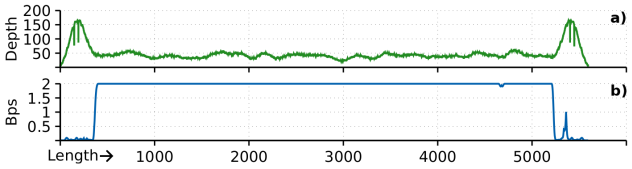

Figure 3. Redundancy controls with coverage (a) and complexity (b) profiles for a B19V DNA sequence identified in an organ. Depth stands for depth coverage while Bps for Bits per base. Lower values of Bps mean higher redundancy. The length scale is in nucleotides.

We found that the highest SNP values reported by dnadiff were for HHV4. These corresponded to regions that are very complex to assemble, both by alignment-based and de-novo approaches, due to statistical ambiguity created by the sequencing noise (multiple repeats and regions of low complexity). Supplementary Figure 4 shows the amount of low complexity (redundant) regions of HHV4 compared to the remaining herpesvirus. Since the genome is near 170kb long, the number of SNPs easily escalate when noise or mutations are added. Moreover, in some cases, we found slightly higher coverage values than those simulated in the intermediary state of alignments (Supplementary table 1). These were given by similarity between different regions, as we opted not to normalize the coverage, nor to apply any equivalent method, but instead to use complexity analysis after duplicate removal. Accordingly, we crossed the complexity profiles with the coverage profiles. In the tips of the B19V genome two areas of high depth coverage were distinguishable, the inverted terminal repeats (ITR), classified as low by our complexity analysis. An example of this analysis is presented in Figure 3, with the B19V DNA identified in blood.

When assessing the individual identity percentage (D - grey background), for a simulated coverage depth of 40 (D - white background), we recovered full genomes without any alterations. Yet, even at low-depth coverage and high levels of SNPs, TRACESPipe was able to reconstruct the genomes with

excellent identity. The lowest values were found for HHV4 in hair and liver (98.6). The hair dataset was simulated with 5x coverage, re-creating regions with gaps or base call ambiguity, while the lung was simulated with 10x coverage and one percent of random mutations. A high-mutation-rate test was conducted for TTV in kidney, in which a 20x coverage and a 15% mutation rate were simulated. Also in this case, TRACESPipe was able to reconstruct the genome with 100% identity and without SNPs in relation to the original sequence. Furthermore, an extreme test was run for B19V in the heart, in which a 20x coverage and 20% mutation rate (1 SNP every 5 bases) were mimicked. Despite these conditions, TRACESPipe was able to reconstruct efficiently the B19V genome showing an identity of 100% without SNPs according to the original sequence. For a representation the dissimilarities between 0% and 1% SNPs, see Supplementary Figure 1.

As shown in Table 2, all the viral and mitogenomes in the samples were identified and efficiently reconstructed (without false positives). A FASTA sequence for each genome was generated along with the necessary controls.

In addition, we evaluated the automatic detection and reconstruction of hybrid viral genomes (defined as combinations of viral sequences). For this purpose, we re-created concatenations of extractions from B19V and VARV sequences using different mutation rates in blood, brain, and bone. The simulation process presented in HybridSpecies.sh is described in Supplementary Section 2. Thereafter, we simulated the sequencing process as previously described, and evaluated the differences between the original hybrid and the reconstructed genomes. The results are presented in Supplementary Table 2 showing full reconstruction with 100% identity.

Together, these results prove the efficiency of TRACESPipe in the identification and reconstruction of viral and human mitochondrial genomes, at multi-organ level, even when prompted with low coverage and high mutation rates.

Real Data

We tested the performance of TRACESPipe in the identification of viral DNA reads derived from different tissues of a recently deceased individual. The organs analyzed were bone, bone marrow, brain, heart, kidney, liver, lung, blood and skin. Each sample was processed individually in the laboratory prior to sequencing in Novaseq (Illumina) with 150 paired-end reads. After de-multiplexing, the sequenced reads were split according to the organ of origin. TRACESPipe identified several genomes, of which JC polyomavirus (Figure 5), human parvovirus B19 (Figure 6), and the human mitogenome are here presented as examples. The percentages of breadth coverage of the mapped reads against the best reference for each organ are depicted in Figure 4a together with the percentage of aligned bases and nucleotide identity for JCPyV and B19V in Figure 4b and c, respectively. The alignments of the reads for JCPyV and the human mitogenome in selected organs can be seen in Supplementary Figure 3.

A Blastn [41] search of the generated consensus sequences of JCPyV from kidney and liver showed an average nucleotide identity of 99% (only few gaps). All the SNPs were congruent between organs, with high coverage. In the skin, the number of reads that aligned to the reference was insufficient; yet, identical SNPs were detected. Figure 5 depicts the alignments and consensus of JCPyV for the organs with highest identity along with the genome map and complexity profile. JCPyV does not contain large redundant parts, enabling easier reconstruction of the complete genome.

Similar analysis was performed for B19V (Figure 6), which displayed a lower number of SNPs compared to JCPyV. Also in this case, B19V showed extremely low DNA variability between organs, allowing for reconstruction of a full consensus derived from the merging of each of the organ sequences.

The mitogenome consensus sequences of ten organs were almost identical (Supplementary Figure 2). The only exception was colon in which a single SNP was absent. We verified that the area where this SNP was located, was only covered minimally. Thus, if we would have relied on the data from colon exclusively, this mutation would have been missed. This finding emphasizes the benefits of comparing the data from different organs as part of the validation process. The final consensus, derived from all the organs, showed 100% in both identity and aligned bases and the presence of 18 SNPs with respect to the reference (Supplementary Figure 5 includes the positions and variations).

The genomes of B19V, JCPyV and human mitogenome were fully assembled with coverages of >25x, >40x, and >80x, respectively. These genomes are available as supplementary material and were uploaded into the TRACES database, freely available as FASTA format files [42]. Additionally, these genomes have been updated into the GenBank with the following accession codes: MT682520 (B19V), MT682521 (JCPyV), MT682522 (mitogenome). The reads to generate the analysis are available in SRA under the code: PRJNA644600.

The congruent patterns of SNPs across multiple tissues, both for the viral (B19V and JCPyV) and mitogenomes suggests that the within-host varibility is minimal. This confers an advantage for the final output of the data, in terms of quality and resolution, and demonstrates the value of this pipeline in combining the information at multi-organ level.

Conclusions

TRACESPipe is an automatic and efficient pipeline for the reconstruction and analysis of viral genomes. It profits from the synergy between reference-based and -free approaches to rise the quality and certainty of prediction to a high level. Indeed, the pipeline performed outstandingly in assignment and reconstruction of viral genomes even when high mutation rates were simulated.

As a unique feature, it supports the merging of data from multiple organ samples. This gives an advantage over existing tools, by permitting the evaluation of intra-host genomic diversity. In terms of the viral populations persisting in the body, this opens the way for the investigation of a diverse range of topics, such as viral tissue tropism, evolution, fitness and disease associations.

Moreover, the extremely low within-host variability of viral genomes and human mitogenomes in different organs, as observed here, may signify and advantage for efficient and complete genome assembly. In fact, the quality of data could be significantly improved by merging complementary sequencing reads between organs towards a robust sequence genome. This may be particularly useful in the scenario of highly fragmented DNA samples, with genomic regions missing, degraded or with high-degree damage, as is frequently the case of ancient DNA.

Another special component of TRACESPipe is the analysis of mitogenomes. Besides serving as a control for external contamination, the cross-association of the viral types with the geographical distribution of this marker can be extremely valuable in epidemiological or archaeovirological studies as well as in forensic investigations, to evaluate the origins of unidentified individuals [43, 44].

Additional features such as encryption and numerous quality and contamination controls make TRACESPipe a robust tool for comprehensive analysis of genomic data.

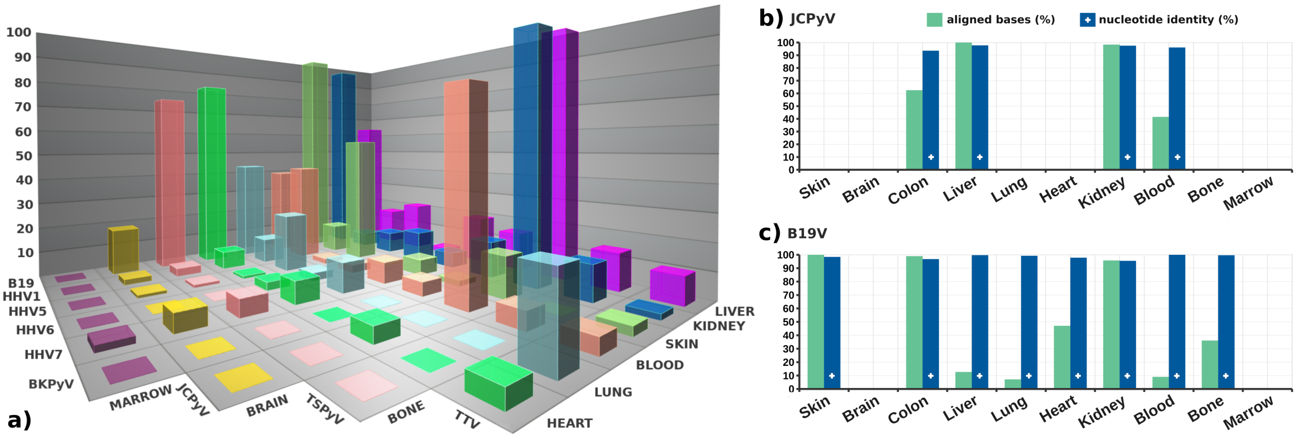

Figure 4. a) Breadth coverage percentage (z-axis) of the (real) mapped reads against the best reference virus for each organ sample. The plot is restricted to viral types with a minimum similarity of 10% in at least one of the organs. The bottom corner had shallow values, which due to space constraints were not included. b,c) Percentage of aligned bases (green) and nucleotide identity (blue) between the best reference and reconstructed genomes of JCPyV and B19V, respectively, calculated using dnadiff. Low breadth coverages may not have corresponding aligned-data values as they may have fallen under the minimal quality or similarity thresholds. The latter was set before the run to exclude noise.

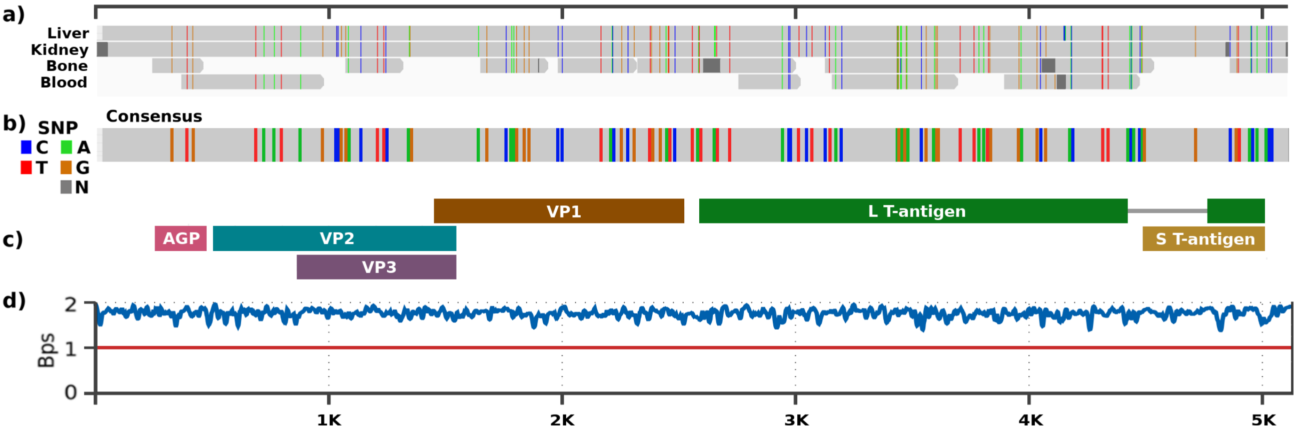

Figure 5. Visualization of the consensus alignments of JCPyV, with the basic structure and complexity profile. a) JCPyV consensus sequences from four organs aligned to the *U61771.1* reference using BWA. Vertical lines stand for SNPs with the respective nucleotide. The dark grey regions stand for gaps (N); b) final consensus merged from a), with SNPs thickened for visualization purposes; c) JCPyV structure with main proteins; d) complexity profile; Bps values <1 correspond to repetitive data. The JCPyV consensus sequences were computed after duplicate removal. a,b) maps were adapted from the IGV after TRACESPipe computation.

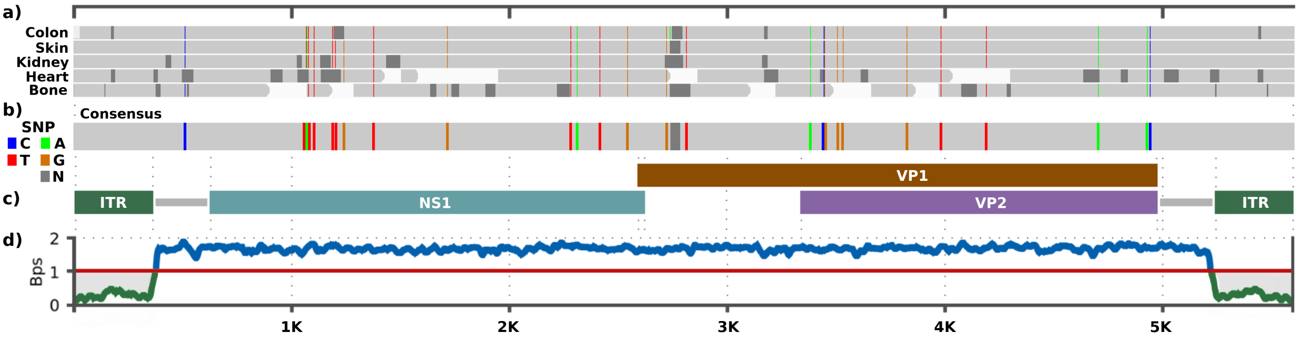

Figure 6. Visualization of the consensus alignments for B19V with the basic structure and complexity profile. a) B19V consensus sequences from five organs aligned to the *KM*393164.1 reference using BWA. Vertical lines stand for SNPs with the respective nucleotide. The dark grey regions stand for gaps (N); b) final consensus built from a), with SNPs thickened for visualization purposes; c) B19V structure including main proteins and inverted terminal repeats (ITR) ; d) complexity profile; where lower regions (ITR) represent repetitive data (Bps< 1). a,b) maps were adapted from the IGV after TRACESPipe computation.

Availability of source code and requirements

- Project name: TRACES Pipeline
- Home page: https://github.com/viromelab/tracespipe
- Operating system(s): Linux / Unix
- Programming language: Shell
- License: GNU GPL3
- RRID: SCR_018831
- Biotools: tracespipe

Availability of supporting data and materials

Raw data are available at the SRA [45] (PRJNA644600). Genome assembly data products are available at the GenBank [46] with

the codes MT682520 (B19V), MT682521 (JCPyV), MT682522 (mitogenome). All supporting data and materials are available at the GigaScience database (GigaDB) [47].

Additional File

Supplementary information: Supplementary File is available via the additional file associated with this article.

Declarations

List of abbreviations

BWA: Burrows Wheeler Aligner; B19V: Human parvovirus B19; DNA: deoxyribonucleic acid; dsDNA: double stranded Deoxyribonucleic acid; GPL: GNU Public License; HPV: human papillomavirus; HHV: human herpesvirus; JCPyV: JC polyomavirus; MT: mitogenome; NCBI: National Center for Biotechnology Information; NGS: next-generation sequencing; NRS: Normalized Relative Similarity; SNP: Single Nucleotide Polymorphism; ssDNA: single stranded deoxyribonucleic acid; TTV: torque teno virus; VARV: variola virus; VCF: variant call format;

Ethical Approval

The study using tissues from autopsies performed at the Department of Forensic Medicine of Helsinki University was reviewed by the Ethics Committee of the Helsinki and Uusimaa Hospital district, dosier number : 164/13/03/00/114.

Competing Interests

The authors declare that they have no competing interests.

Funding

This work was partially funded by national funds through the FCT in the context of the project UIDB/00127/2020. Also by the Finnish Medical Foundation, Finnish Cultural Foundation, Juhani Aho Foundation for Medical Research, Jane & Aatos Erkko Foundation, Medicinska Understödsföreningen Liv och Hälsa, Kone Foundation, Magnus Ehrnrooth Foundation, the Finnish Society of Sciences and Letters, the Research Funds of University of Helsinki and Helsinki University Hospital. D.P. is funded by national funds through FCT - Fundação para a Ciência e a Tecnologia, I.P., under the Scientific Employment Stimulus - Institutional Call - CI-CTTI-94-ARH/2019.

Author's Contributions

D.P., A.S, and M.P. conceived and designed the experiments; D.P., M.T., L.P. performed the experiments; D.P., M.T., L.P., K.H., A.S., and M.P. analyzed the data; D.P., M.T., L.P., K.H., A.S., and M.P. wrote the paper.

References

1. Simmonds P. Methods for virus classification and the challenge of incorporating metagenomic sequence data. Journal of General Virology 2015;96(6):1193–1206.
2. Li Y, Wang H, Nie K, Zhang C, Zhang Y, Wang J, et al. VIP: an integrated pipeline for metagenomics of virus identification and discovery. Scientific reports 2016;6:23774.
3. Ren J, Ahlgren NA, Lu YY, Fuhrman JA, Sun F. VirFinder: a novel k-mer based tool for identifying viral sequences from assembled metagenomic data. Microbiome 2017;5(1):69.
4. Rampelli S, Soverini M, Turroni S, Quercia S, Biagi E, Brigidi P, et al. ViromeScan: a new tool for metagenomic viral community profiling. BMC genomics 2016;17(1):165.
5. Laffy PW, Wood-Charlson EM, Turaev D, Weynberg KD, Botté ES, van Oppen MJ, et al. HoloVir: a workflow for investigating the diversity and function of viruses in invertebrate holobionts. Frontiers in microbiology 2016;7:822.
6. Bolduc B, Youens-Clark K, Roux S, Hurwitz BL, Sullivan MB. iVirus: facilitating new insights in viral ecology with software and community data sets imbedded in a cyberinfrastructure. The ISME journal 2017;11(1):7–14.
7. Ajami NJ, Wong MC, Ross MC, Lloyd RE, Petrosino JF. Maximal viral information recovery from sequence data using VirMAP. Nature communications 2018;9(1):1–9.
8. Tithi SS, Aylward FO, Jensen RV, Zhang L. FastViromeExplorer: a pipeline for virus and phage identification and abundance profiling in metagenomics data. PeerJ 2018;6:e4227.
9. Vilsker M, Moosa Y, Nooij S, Fonseca V, Ghysens Y, Dumon K, et al. Genome Detective: an automated system for virus identification from high-throughput sequencing data. Bioinformatics 2018;35(5):871–873.
10. Hosseini M, Pratas D, Pinho AJ. Cryfa: a secure encryption tool for genomic data. Bioinformatics 2018;35(1):146–148.
11. Pratas D, Hosseini M, Grilo G, Pinho A, Silva R, Caetano T, et al. Metagenomic Composition Analysis of an Ancient Sequenced Polar Bear Jawbone from Svalbard. Genes 2018;9(9):445.
12. Langmead B, Salzberg SL. Fast gapped-read alignment with Bowtie 2. Nature Methods 2012;9(4):357.
13. Li H. A statistical framework for SNP calling, mutation discovery, association mapping and population genetical parameter estimation from sequencing data. Bioinformatics 2011;27(21):2987–2993.
14. Nurk S, Meleshko D, Korobeynikov A, Pevzner PA. metaSPAdes: a new versatile metagenomic assembler. Genome Research 2017;27(5):824–834.
15. Li H, Durbin R. Fast and accurate long-read alignment with Burrows-Wheeler transform. Bioinformatics 2010;26(5):589–595.
16. Robinson JT, Thorvaldsdóttir H, Winckler W, Guttman M, Lander ES, Getz G, et al. Integrative genomics viewer. Nature biotechnology 2011;29(1):24.
17. The Cygwin project. https://www.cygwin.com/. Accessed 20 June 2020.
18. Hernaez M, Pavlichin D, Weissman T, Ochoa I. Genomic Data Compression. Annual Review of Biomedical Data Science 2019;2.
19. Kircher M. Analysis of high-throughput ancient DNA sequencing data. In: Ancient DNA Springer; 2012.p. 197–228.
20. Bolger AM, Lohse M, Usadel B. Trimmomatic: a flexible trimmer for Illumina sequence data. Bioinformatics 2014;30(15):2114–2120.
21. Pratas D, Pinho AJ. Metagenomic composition analysis of sedimentary ancient DNA from the Isle of Wight. In: 2018 26th European Signal Processing Conference (EUSIPCO) IEEE; 2018. p. 1177–1181.
22. Almeida JR, Pinho AJ, Oliveira JL, Fajarda O, Pratas D. GTO: a toolkit to unify pipelines in genomic and proteomic research. SoftwareX 2020;12: 100535.
23. Wheeler DL, Barrett T, Benson DA, Bryant SH, Canese K, Chetvernin V, et al. Database resources of the national center for biotechnology information. Nucleic Acids Research

2006;35(suppl_1):D5–D12.
24. The NCBI Viral database. https://www.ncbi.nlm.nih.gov/labs/virus/vssi/. Accessed 20 June 2020.
25. Zielezinski A, Girgis HZ, Bernard G, Leimeister CA, Tang T Kujiand Dencker, Lau AK, et al. Benchmarking of alignment-free sequence comparison methods. Genome biology 2019; 20(1):144.
26. Pratas D, Hosseini M, Pinho AJ. Substitutional Tolerant Markov Models for Relative Compression of DNA Sequences. In: 11th Int. Conf. on Practical Applications of Computational Biology & Bioinformatics Springer; 2017. p. 265–272.
27. Li H. Tabix: fast retrieval of sequence features from generic TAB-delimited files. Bioinformatics 2011;27(5):718–719.
28. Quinlan AR. BEDTools: the Swiss-army tool for genome feature analysis. Current protocols in bioinformatics 2014;47(1):11–12.
29. Paez-Espino D, Eloe-Fadrosh EA, Pavlopoulos GA, Thomas AD, Huntemann M, Mikhailova N, et al. Uncovering Earth's virome. Nature 2016;536(7617):425–430.
30. Pinho AJ, Garcia SP, Pratas D, Ferreira PJ. DNA sequences at a glance. PloS one 2013;8(11):e79922.
31. Li H, Handsaker B, Wysoker A, Fennell T, Ruan J, Homer N, et al. The sequence alignment/map format and SAMtools. Bioinformatics 2009;25(16):2078–2079.
32. Budowle B, Connell ND, Bielecka-Oder A, Colwell RR, Corbett CR, Fletcher J, et al. Validation of high throughput sequencing and microbial forensics applications. Investigative genetics 2014;5(1):9.
33. Andrews RM, Kubacka I, Chinnery PF, Lightowlers RN, Turnbull DM, Howell N. Reanalysis and revision of the Cambridge reference sequence for human mitochondrial DNA. Nature genetics 1999;23(2):147.
34. Anderson S, Bankier AT, Barrell BG, de Bruijn MH, Coulson AR, Drouin J, et al. Sequence and organization of the human mitochondrial genome. Nature 1981;290(5806):457–465.
35. Jónsson H, Ginolhac A, Schubert M, Johnson PL, Orlando L. mapDamage2.0: fast approximate Bayesian estimates of ancient DNA damage parameters. Bioinformatics 2013;29(13):1682–1684.
36. Zhang Z, Schwartz S, Wagner L, Miller W. A greedy algorithm for aligning DNA sequences. Journal of Computational biology 2000;7(1-2):203–214.
37. Grüning B, Dale R, Sjödin A, Chapman BA, Rowe J, Tomkins-Tinch CH, et al. Bioconda: sustainable and comprehensive software distribution for the life sciences. Nature methods 2018;15(7):475.
38. The Cobilab project. https://github.com/cobilab. Accessed 20 June 2020.
39. Marçais G, Delcher AL, Phillippy AM, Coston R, Salzberg SL, Zimin A. MUMmer4: a fast and versatile genome alignment system. PLoS computational biology 2018;14(1):e1005944.
40. Huang W, Li L, Myers JR, Marth GT. ART: a next-generation sequencing read simulator. Bioinformatics 2011;28(4):593–594.
41. Ye J, McGinnis S, Madden TL. BLAST: improvements for better sequence analysis. Nucleic acids research 2006;34(2):W6–W9.
42. The Virome group website. https://viromelab.github.io/. Accessed 20 June 2020.
43. Toppinen M, Perdomo M, Palo J, Simmonds P, Lycett S, Söderlund-Venermo M, et al. Bones hold the key to DNA virus history and epidemiology. Scientific reports 2015;5:17226.
44. Forni D, Cagliani R, Clerici M, Pozzoli U, Sironi M. You Will Never Walk Alone: Codispersal of JC Polyomavirus with Human Populations. Molecular biology and evolution 2019.
45. The Sequence Read Archive project. https://www.ncbi.nlm.nih.gov/sra. Accessed 20 June 2020.
46. The GenBank project. https://www.ncbi.nlm.nih.gov/genbank/. Accessed 20 June 2020.
47. Pratas D, Toppinen M, Pyöriä L, Hedman K, Sajantila A, Perdomo MF. Supporting data for "A hybrid pipeline for reconstruction and analysis of viral genomes at multi-organ level". GigaScience Database 2020. http://dx.doi.org/10.5524/100771.

Click here to access/download;Manuscript;main.pdf

Your PDF file "main.pdf" cannot be opened and processed. Please see the common list of problems, and suggested resolutions below.

Reason:

Other Common Problems When Creating a PDF from a PDF file

You will need to convert your PDF file to another format or fix the current PDF file, then re-submit it.

Click here to access/download
Supplementary Material
TRACESPipe_Supplementary_Material.pdf
