# Supplementary material for: A hybrid pipeline for reconstruction and analysis of viral genomes at multi-organ level
Source: Gigascience. 2020 Aug 20;9(8):giaa086. doi: 10.1093/gigascience/giaa086 (PMC7439602; doi:10.1093/gigascience/giaa086)
Supplement: giaa086_Supplemental_File [file giaa086_supplemental_file.pdf]

Supplementary material of
“A hybrid pipeline for reconstruction and analysis of viral genomes at multi-organ level”

D. Pratas, M. Toppinen, L. Pyöriä, K. Hedman, A. Sajantila, M. Perdomo

1 Supplementary Figures and Tables

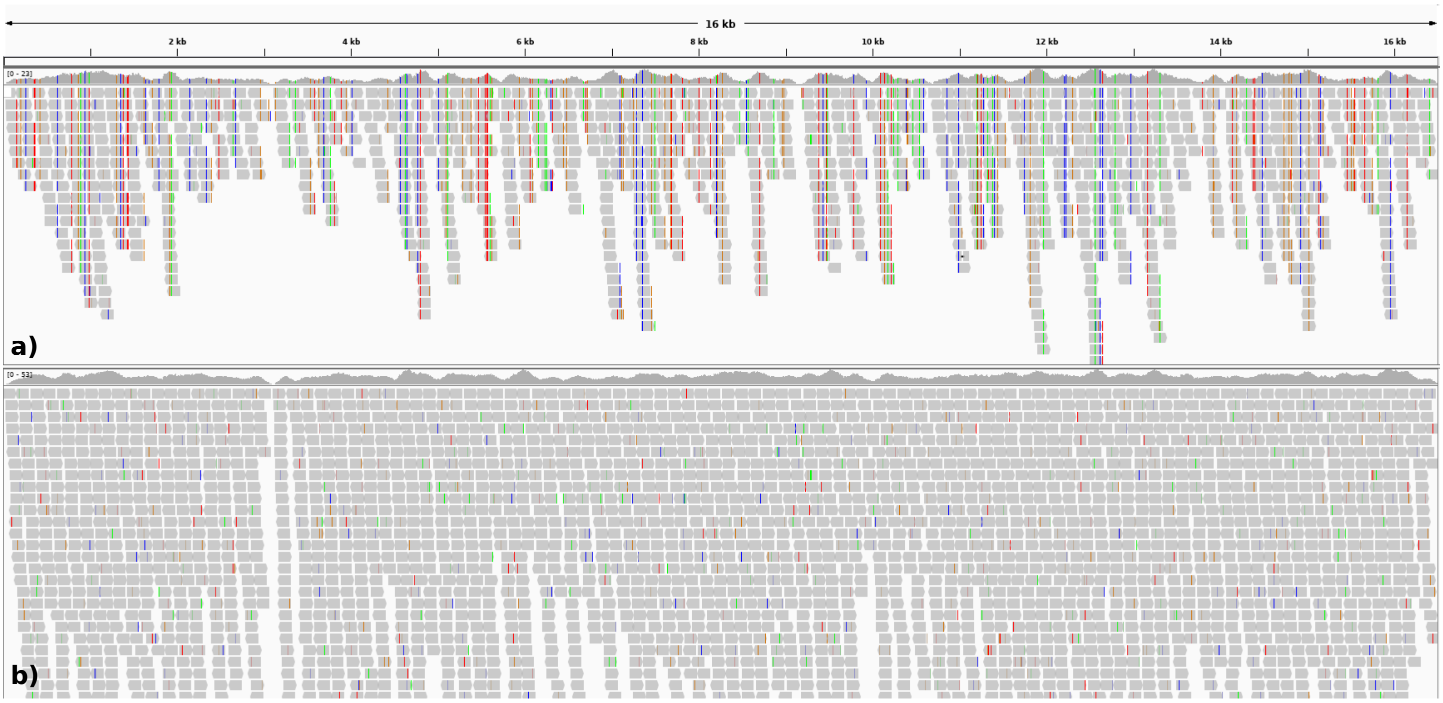

Figure S1: Alignments using Bowtie2 of simulated mitochondrial reads relative to the reference genome. a) the sequence is mutated with 1% substitutions in a brain sample, simulated with 10x depth coverage; b) sequence without mutations from a bone sample with a simulated depth coverage of 30. The identified SNPs are highlighted with vertical colored stripes. Visual map extracted from IGV.

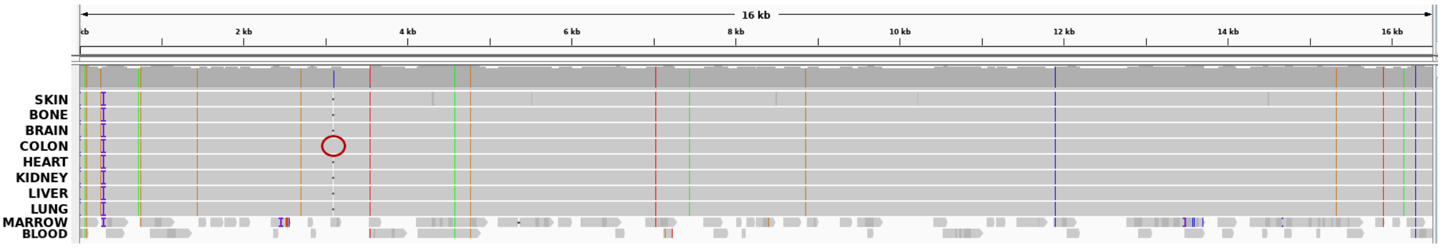

Figure S2: Alignments using BWA of the consensus sequences of real mitochondrial sequences from multiple organs. The identified SNPs are highlighted with vertical colored stripes. Consensus SNPs: 72 T→A, 93 A→G, 263 A→G, 309 +CT, 722 C→A, 750 A→G, 1438 A→G, 2706 A→G, 3106 -C, 3549 C→T, 4580 G→A, 4769 A→G, 7028 C→T, 7444 G→A, 8860 A→G, 11899 T→C, 15326 A→G, 15904 C→T, 16153 G→A, 16298 T→C. Visual map extracted from IGV.

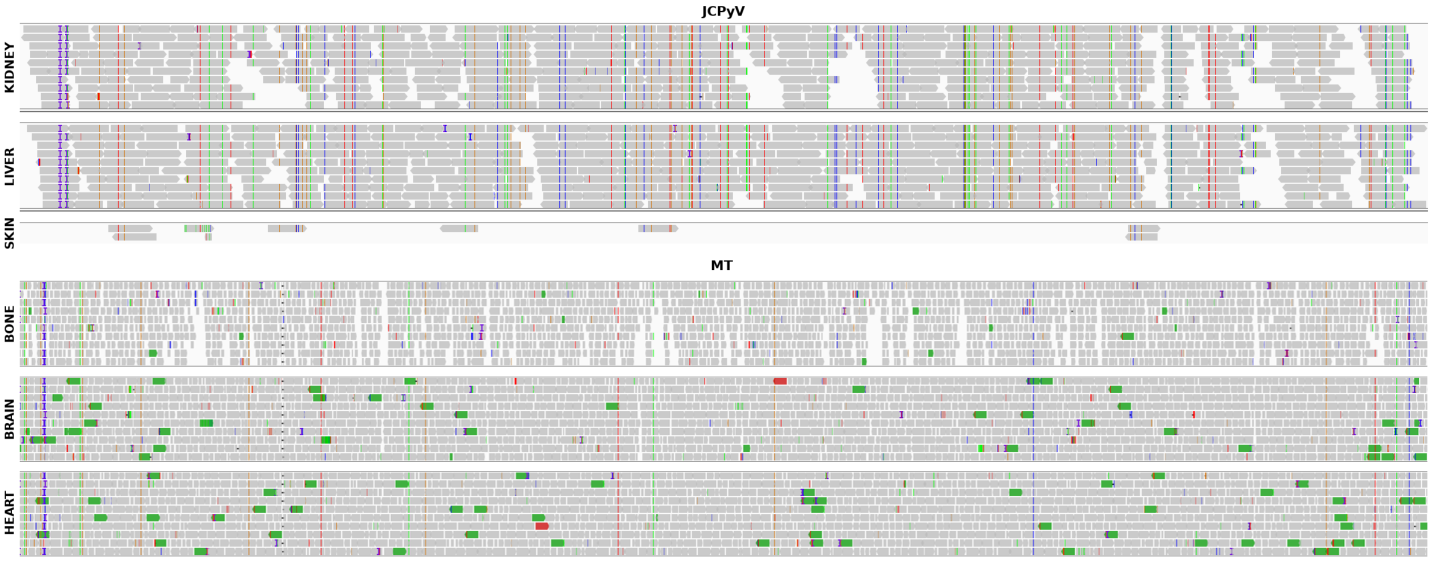

Figure S3: Visualization of the read alignments of each organ sample for JCPyV and the human mitogenome (MT). The maps have been adapted from the IGV. Each map shows the complete alignments in the respective scale. The JCPyV maps include duplicate removal while the MT maps contain the duplications, as an example of TRACESPipe being able to filter or maintain the duplications (reads highlighted green by IGV show possible duplications). Vertical lines stand for SNPs. Red reads refer to an inferred insert size that is larger than expected (possible evidence of a deletion).

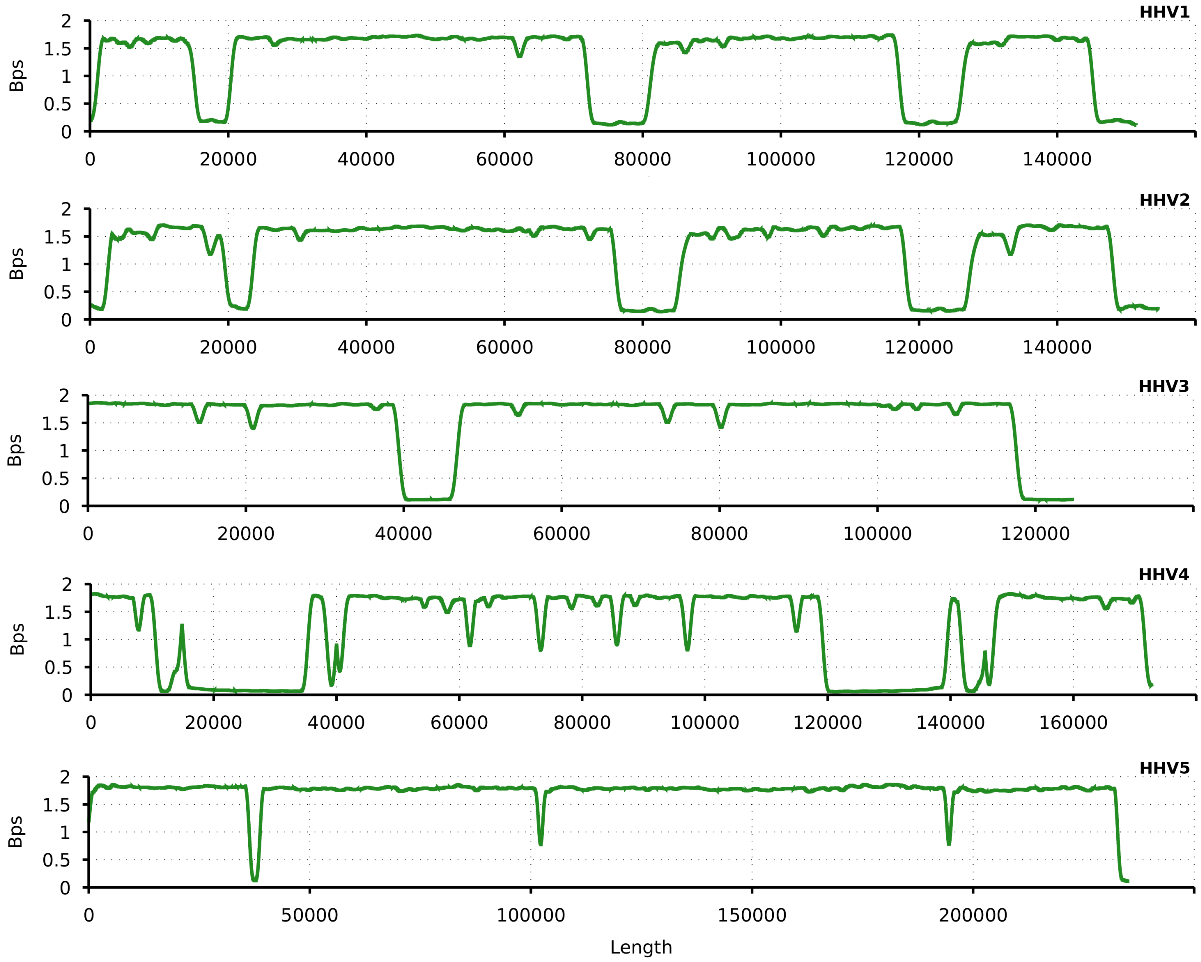

Figure S4: Complexity profiles for several Human Herpesvirus (HHV1, HHV2, HHV3, HHV4, and HHV5). Lower regions correspond to close or distant repetitive regions. The profiles were computed with TRACESPipe using GTO.

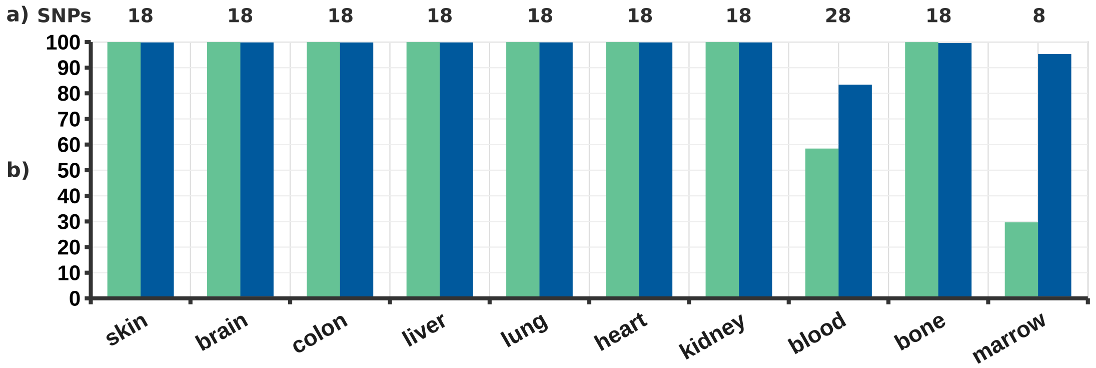

Figure S5: a) Number of SNPs; b) percentage of aligned bases (green) and nucleotide identity (blue) of the human mitogenome reference relative to the reconstructed sequence. The number of SNPs, percentage of aligned bases, and nucleotide identity have been automatically computed with TRACESPipe using dnadiff from the Mummer4 package.

	Blood			Bone			Brain			Hair			Heart			Kidney			Liver			Lung			Skin			Teeth		
SEQ	F	D	S	F	D	S	F	D	S	F	D	S	F	D	S	F	D	S	F	D	S	F	D	S	F	D	S	F	D	S
B19V	✓	40	0	✓	30	1	✓	10	0	✗	-	-	✓	20	20	✓	20	0	✗	-	-	✗	-	-	✓	25	0	✓	30	5
	✓	48.3	100	✓	36.0	100	✓	12.4	100	✗	0.0	0	✓	0.1	5.5	✓	24.9	100	✗	0.0	0	✗	0.0	0	✓	28.2	100	✓	32.3	100
HHV2	✓	40	0	✗	-	-	✗	-	-	✗	-	-	✗	-	-	✓	20	0	✗	-	-	✗	-	-	✗	-	-	✓	30	0
	✓	58.3	100	✗	0.0	0	✗	0.0	0	✗	0.0	0	✗	0.0	0	✓	28.7	100	✗	0.0	0	✗	0.0	0	✗	0.0	0	✓	43.4	100
HHV3	✓	40	0	✗	-	-	✗	-	-	✗	-	-	✗	-	-	✗	-	-	✗	-	-	✗	-	-	✓	25	0	✗	-	-
	✓	45.3	100	✗	0.0	0	✗	0.0	0	✗	0.0	0	✗	0.0	0	✗	0.0	0	✗	0.0	0	✗	0.0	0	✓	28.8	100	✗	0.0	0
HHV4	✗	-	-	✗	-	-	✓	10	0	✓	5	0	✗	-	-	✗	-	-	✓	20	1	✓	10	1	✗	-	-	✗	-	-
	✗	0.0	0	✗	0.0	0	✓	51.7	99.9	✓	24.4	96.6	✗	0.0	0	✗	0.0	0	✓	92.5	100	✓	51.6	99.9	✗	0.0	0	✗	0.0	0
HHV8	✓	40	0	✗	-	-	✗	-	-	✗	-	-	✗	-	-	✗	-	-	✗	-	-	✗	-	-	✗	-	-	✗	-	-
	✓	106	100	✗	0.0	0	✗	0.0	0	✗	0.0	0	✗	0.0	0	✗	0.0	0	✗	0.0	0	✗	0.0	0	✗	0.0	0	✗	0.0	0
HPV	✗	-	-	✗	-	-	✗	-	-	✓	5	10	✓	20	10	✗	-	-	✓	20	0	✗	-	-	✗	-	-	✗	-	-
	✗	0.0	0	✗	0.0	0	✗	0.0	0	✓	3.3	86.4	✓	12.5	98	✗	0.0	0	✓	19.9	99.8	✗	0.0	0	✗	0.0	0	✗	0.0	0
TTV	✗	-	-	✓	30	10	✗	-	-	✗	-	-	✗	-	-	✓	20	15	✗	-	-	✗	-	-	✓	25	0	✓	30	0
	✗	0.0	0	✓	18.3	87.6	✗	0.0	0	✗	0.0	0	✗	0.0	0	✓	2.0	28.9	✗	0.0	0	✗	0.0	0	✓	24.2	96.5	✓	29.1	99.7
VARV	✓	40	0	✗	-	-	✓	10	0	✗	-	-	✓	20	5	✗	-	-	✓	20	0	✓	10	0	✗	-	-	✗	-	-
	✓	43.7	100	✗	0.0	0.0	✓	10.9	99.8	✗	0.0	0.0	✓	21.8	100	✗	0.0	0.0	✓	22.1	100	✓	11.1	99.8	✗	0.0	0.0	✗	0.0	0.0
MT	✓	40	0	✓	30	0	✓	10	1	✓	5	0	✓	20	0	✓	20	1	✓	20	2	✓	10	0	✓	25	0	✓	30	5
	✓	39.2	99.9	✓	29.5	99.8	✓	9.8	99.3	✓	4.9	96.1	✓	19.7	99.7	✓	19.7	99.7	✓	19.8	99.8	✓	9.8	99.4	✓	24.6	99.8	✓	28.7	99.3

Table S1: Benchmark of TRACESPipe (depth and breadth coverage) in viral and mitogenomes from 10 different organs. In each SEQ line, the upper line is the statistical ground truth (simulation conditions), while the bottom line represents the TRACESPipe output. The F stands for the existence or not of the respective virus in the organ sample, where the ✓stands for viral or mitochondrial genome detection in the sample, while ✗ for the opposite. The D stands for the depth coverage and S for the breadth coverage. To replicate use script Benchmark.sh from the repository.

Organ name	Virus name	Genome length	Aligned bases (%)	Identity (%)	Number of SNPs
Bood	B19V	5773	100.00	100.00	0
Bood	VARV	5773	100.00	100.00	0
Bone	B19V	4991	100.00	100.00	0
Bone	VARV	4991	100.00	100.00	0
Brain	B19V	6773	100.00	100.00	0
Brain	VARV	6773	100.00	100.00	0

Table S2: Benchmark of TRACESPipe when hybrid viral species with mutated parts are present in the data. The FASTQ data was simulated with ART. The aligned bases, genome identity and numbers of SNPs refer to the comparison between the original and reconstructed sequences. To replicate use script HybridSpecies.sh from the repository.

2 Reproducibility

2.1 Installation

To install TRACESPipe, the following commands must be executed

```
git clone https://github.com/viromelab/tracespipe.git
```

```
cd tracespipe/src/
chmod +x TRACES*.sh
./TRACESPipe.sh --install
./TRACESPipe.sh --get-all-aux
```

2.2 Configuration

Since the pipeline considers multi-organs, there is a simple configuration process that must be followed, namely:

- Adding the **FASTQ** files **gziped** at the **input_data** folder.
- Adding a file exclusively with name **meta_info.txt** at the folder **meta_data**.

This **meta_info.txt** file needs to specify the organ type (single word name) and the filenames for the paired end reads. An example of the content of meta_info.txt is the following:

```
skin:V1_S44_R1_001.fastq.gz:V1_S44_R2_001.fastq.gz
brain:V2_S29_R1_001.fastq.gz:V2_S29_R2_001.fastq.gz
colon:V3_S45_R1_001.fastq.gz:V3_S45_R2_001.fastq.gz
```

2.3 Benchmark

2.3.1 Synthetic benchmark

The following commands run the synthetic benchmark

```
cd ../demos
./Benchmark.sh 2>> tmp
```

The output provided by TRACESPipe is the following

```
blood
B19  5596(100.00%) 100.00  0
HV2  154675(100.00%) 100.00  0
HV3  124875(99.99%)  100.00  0
HV8  137961(99.99%)  100.00  0
VARV  185559(99.99%)  100.00  0
mtDNA  16557(99.93%) 99.98 0

bone
B19  5596(100.00%) 99.96 2
TTV  2784(99.96%)  100.00  0
mtDNA  16546(99.86%) 99.98 0

brain
B19  5596(100.00%) 100.00  0
HV4  172743(99.99%)  99.93 2
VARV  185424(99.92%)  99.87 2
mtDNA  16539(99.82%) 99.53 1

hair
HV4  168866(97.74%)  98.77 11
HPV  7070(95.89%)  98.84 0
mtDNA  16127(97.33%) 98.79 1

heart
B19  5588(100.00%) 100.00  0
HPV  7351(99.72%)  100.00  0
VARV  185571(100.00%) 99.99 21
mtDNA  16539(99.82%) 99.92 0

kidney
B19  5596(100.00%) 100.00  0
HV2  154675(100.00%) 100.00  0
TTV  2756(99.96%)  100.00  0
mtDNA  16539(99.82%) 99.92 0

liver
HV4  172728(99.98%)  99.85 264
HPV  7358(99.81%)  100.00  0
VARV  185555(99.99%)  100.00  0
mtDNA  16549(99.88%) 99.87 0

lung
```

```
HV4 172699(99.96%)  99.80 286
VARV  185388(99.90%)  99.91 1
mtDNA 16539(99.82%) 99.53 1

skin
B19 5596(100.00%) 100.00  0
HV3 124880(100.00%) 100.00  0
TTV 2687(96.48%)  100.00  0
mtDNA 16546(99.86%) 99.98 0

teeth
B19 5594(99.96%)  99.91 5
HV2 154675(100.00%) 100.00  0
TTV 2775(99.64%)  100.00  0
mtDNA 16510(99.64%) 99.69 0
```

2.3.2 Hybrid species test

The following commands (at demos/ folder) run the hybrid species Benchmark

```
./HybridSpecies.sh 2>> tmp
```

The output provided by TRACESPipe is the following

```
Bood
B19 5773(100.00%) 100.00  0
VARV  5773(100.00%) 100.00  0

Bone
B19 4991(100.00%) 100.00  0
VARV  4991(100.00%) 100.00  0

Brain
B19 6773(100.00%) 100.00  0
VARV  6773(100.00%) 100.00  0
```

2.4 Data analysis

The reads were copied into the input_data and the whole data analysis was configured with

```
skin:V17_S57_R1_001.fastq.gz:V17_S57_R2_001.fastq.gz
brain:V18_S31_R1_001.fastq.gz:V18_S31_R2_001.fastq.gz
colon:V19_S32_R1_001.fastq.gz:V19_S32_R2_001.fastq.gz
liver:V20_S33_R1_001.fastq.gz:V20_S33_R2_001.fastq.gz
lung:V21_S58_R1_001.fastq.gz:V21_S58_R2_001.fastq.gz
heart:V22_S42_R1_001.fastq.gz:V22_S42_R2_001.fastq.gz
kidney:V23_S59_R1_001.fastq.gz:V23_S59_R2_001.fastq.gz
blood:V24_S60_R1_001.fastq.gz:V24_S60_R2_001.fastq.gz
bone:V53_S5_R1_001.fastq.gz:V53_S5_R2_001.fastq.gz
marrow:V54_S6_R1_001.fastq.gz:V54_S6_R2_001.fastq.gz
```

The command for the entire ran was

```
./TRACESPipe.sh
  --run-meta                   # It runs the full viral metagenomic composition analysis
  --inter-sim-size 20          # Control: detect similarity between the best 20 references
  --run-all-v-alig             # It runs all the viral alignments and creates consensus sequences
  --run-mito                   # It runs all the mtDNA alignments and creates consensus sequences
  --remove-dup                 # It removes duplications
  --run-de-novo                # It runs de-novo assembly
  --run-hybrid                 # It combines alignment-based with de-novo assembly
  --min-similarity 5           # It only aligns and assembles sequences with similarity above 5%
  --view-top 5                 # It print the best 5 references (not mandatory but gives running info)
  --best-of-bests              # It chooses and forces to use the best reference among the organs
  --very-sensitive             # It uses very sensitive parameters for alignments
  --run-diff                   # It runs diff between best reference & reconstructed (SNPs & identity)
  --run-multiorgan-consensus   # It runs alignments/consensus of all the reconstructed organ sequences
```

The full output is automatically provided at the output_data folder according to

```
TRACES_results/                # where the metagenomic analysis files and control will appear
TRACES_results/profiles/       # where the redundancy profiles appear

TRACES_viral_alignments/       # where viral alignments and index will appear
TRACES_viral_consensus/        # where viral consensus (FASTA) will appear
TRACES_viral_bed/              # where viral BED files will appear (SNPs and Coverage)
```

```
TRACES_viral_statistics/        # where viral statistics appear (depth/wide coverage)

TRACES_mtdna_alignments/        # where mtdna alignments and index will appear
TRACES_mtdna_consensus/         # where mtdna consensus (FASTA) will appear
TRACES_mtdna_bed/               # where mtdna BED files will appear (SNPs and Coverage)
TRACES_mtdna_statistics/        # where mtdna statistics appear (depth/wide coverage)
TRACES_mtdna_authentication/    # where mtdna species and population authentication appears

TRACES_cy_alignments/           # where cy alignments and index will appear
TRACES_cy_consensus/            # where cy consensus (FASTA) will appear
TRACES_cy_bed/                  # where cy BED files will appear (SNPs and Coverage)
TRACES_cy_statistics/           # where cy statistics appear (depth/wide coverage)

TRACES_specific_alignments/     # where specific alignments and index will appear
TRACES_specific_consensus/      # where specific consensus (FASTA) will appear
TRACES_specific_bed/            # where specific BED files will appear
TRACES_specific_statistics/     # where specific statistics appear (depth/wide coverage)

TRACES_mtdna_damage_<ORGAN>/    # where the mtdna damage estimation files will appear

TRACES_denovo_<ORGAN>/          # where the output of de-novo assembly appears

TRACES_hybrid_alignments/       # where the hybrid data appears
TRACES_hybrid_consensus/        # where the hybrid data appears
TRACES_hybrid_bed/              # where the hybrid data appears

TRACES_hybrid_R2_alignments/    # where the second round hybrid data appears
TRACES_hybrid_R2_consensus/     # where the second round hybrid data appears
TRACES_hybrid_R2_bed/           # where the second round hybrid data appears

TRACES_hybrid_R3_alignments/    # where the third round hybrid data appears
TRACES_hybrid_R3_consensus/     # where the third round hybrid data appears
TRACES_hybrid_R3_bed/           # where the third round hybrid data appears

TRACES_hybrid_R4_alignments/    # where the fourth round hybrid data appears
TRACES_hybrid_R4_consensus/     # where the fourth round hybrid data appears
TRACES_hybrid_R4_bed/           # where the fourth round hybrid data appears

TRACES_hybrid_R5_consensus/     # where the automatic chosen hybrid consensus
                                # appears (diff will be made using this data)

TRACES_multiorgan_alignments/   # where the multi-organ alignments data appears
TRACES_multiorgan_consensus/    # where the multi-organ consensus data appears

TRACES_diff/                    # where the dnadiff results appear (identity & SNPs)

TRACES_blasts/                  # where the specific blasted results appears
```
