# Supplementary material for: A hybrid pipeline for reconstruction and analysis of viral genomes at multi-organ level
Source: Gigascience. 2020 Aug 20;9(8):giaa086. doi: 10.1093/gigascience/giaa086 (PMC7439602; doi:10.1093/gigascience/giaa086)
Supplement: giaa086_Response_to_Reviewer_Comments_Original_Submission [file giaa086_response_to_reviewer_comments_original_submission.pdf]

Author's Response To Reviewer Comments

Close

Dear Editor,
We greatly appreciate the opportunity given to revise our manuscript. We would like to thank the Reviewers, whose suggestions allowed us to improve our pipeline, and the manuscript, in many ways. We have now addressed the points raised by them as outlined in blue in this revision letter. Following their suggestions, appropriate changes have been introduced to the manuscript, as shown in orange. We trust we have been able to address their concerns and that our manuscript is now suitable for publication at GigaScience.

Reviewer 1

Reviewer: The manuscript entitled ”A hybrid pipeline for reconstruction and analysis of viral genomes at multi-organ level ”, by Pratas et al. describes their development of a novel bioinformatics pipeline named TRACESPipe. Overall this software specializes in assembling and analyzing viral genomes from multiple organ sites. As such, it will enable the rapid analysis of various types of datasets that explore intra-host viral diversity. The workflow is very logical and expresses an impressive knowledge of the tools involved as well as the file formats that are produced. The described methods are appropriate for this study and the authors do an excellent job to ensure that multiple approaches are used to facilitate performing the necessary controls on the data being analyzed. The authors do a good job with ensuring that the conclusions drawn are supported by the data and results reported elsewhere in the manuscript.

Authors: We thank you for the revision and appreciate your comments.

Reviewer: The manuscript could be improved by including additional text to describe: 1) Why 40 was selected as the optimal number of genes with high similarity scores? Can the authors provide additional justification and/or data to reinforce this decision?

Authors: The number 40 stands for the number of reference genomes that are compared all with all. The purpose is to identify possible candidates with given cross-similarity. This comparison has a quadratic complexity and, hence, it may be time-consuming for higher numbers. The number 40 was chosen as a balance between computational time and precision. However, values up to 100 are still affordable. The method permits to use multi-thread to reduce the computational time.
This value can be parameterized through the flag: --inter -sim -size
Following the Reviewer’s request, we have expanded the description on this subject.

Reviewer: The abbreviations used in the caption for Table 2. Although these abbreviations are defined in the List of Abbreviations at the end of the manuscript, readability could be improved by including the relevant abbreviations in the Table caption.1

Authors: We thank the Reviewer for pointing this out. We added the information to the table caption.

Reviewer: Although not required for the current publication, it may be helpful for the authors to consider the below recommendations as part of future development plans: 1) Enable the analysis of mitochondrial sequence from non-human primates. These animals are often used as model organisms for a variety of viruses. As such, the user base of the TRACESPipe software could be expanded by providing this capability.2) Adapt the installation process such that TRACESPipe can be installed into an auto-generated Conda environment (i.e. ”conda install -c bioconda TRACESPipe”)?

Authors: We thank the Reviewer for these suggestions. With regard to the first point, we have now enabled the analysis of any mitochondrial genome in TRACESPipe, using the following command:
TRACESPipe.sh --change-mito
As it stands now, any identifier (ID) can be used. TRACESPipe will automatically download the genome with the respective ID and use it as reference mitochondrial sequence.
Regarding the second point, we thank the Reviewer for the suggestion of including TRACESPipe in

Bioconda. We are big fans of Bioconda and all the conda applications. In fact, we have our own conda channel (cobilab). TRACESPipe installs the software tools automatically using Bioconda and Cobilab channels, with:
TRACESPipe --install
Setting TRACESPipe in Bioconda or cobilab is feasible, however TRACESPipe clone and configuration requires the following steps:
git clone https://github.com/viromelab/tracespipe.git
cd tracespipe/src/
chmod +x TRACES *.sh
TRACESPipe.sh --install #(here bioconda and cobilad are used)
Since it considers multiple organs, there is a simple configuration process that must be followed:
Adding the FASTQ files gziped at the folder: inputdata.
Then, adding a file exclusively with name metainfo.txt at the folder metadata.
This file needs to specify the organ type (with a single word name) and the file names for the paired-end reads. An example of the content of metainfo.txt is the following:
skin:V1_S44_R1_001.fastq.gz:V1_S44_R2_001.fastq.gz
brain:V2_S29_R1_001.fastq.gz:V2_S29_R2_001.fastq.gz
colon:V3_S45_R1_001.fastq.gz:V3_S45_R2_001.fastq.gz.
Then, to get automatically the auxiliary sequences, at the src/ folder, run:
TRACESPipe.sh --get -all -aux
This action permits the analysis of multiple organs from one individual. However, it does not support the analysis of several individuals under the same framework. For this, the main folder must be copied into multiple folders, where under each folder runs the analysis of one individual. According to this, a basic Bioconda installation would not work for multiple individuals. Therefore, to avoid overlaps, we will maintain the clone installation. We will consider the multi-individual development and, subsequently, a full Conda feature, in future versions of TRACESPipe.

Reviewer 2

Reviewer: In this manuscript, the authors present a new pipeline for reconstruction of virus genomes from multiple organs simultaneously. This will be a useful pipeline for analyzing virus data from processing raw reads to downstream analysis as this tool can start working from the raw read data, can do both the alignment of the read and then assembly of the virus genome as well as report the variants found in the reconstructed genomes which will be helpful for the downstream analysis.

Authors: We thank the Reviewer for the comments. The pipeline includes also the analysis of the human-host genome, to which hybrid assembly and variation can be also applied. As requested by reviewer 1, we now included the possibility to use any mitochondrial genome.

Reviewer: Major concerns: 1. As TRACESPipe is a computational pipeline for analyzing virus data, the features of TRACESPipe should be compared with other existing pipelines, i.e., iVirus [1], VirMap [2] to highlight the novel features of TRACESPipe tool and to highlight the difference of this tool from other existing tools.

Authors: We thank the Reviewer for pointing out iVirus and VirMAP. We have now added both references to the repertoire already cited in the manuscript. TRACESPipe stands out from other existing pipelines for its ability to assemble and compare directly sequences derived from multiple organs. Also, not included by many, is the simultaneous run of both reference-based and de-novo assemblies. In addition, TRACESPipe includes unique features such as the analysis of mitochondrial DNA and damage patterns, which are crucial for the investigation of ancientDNA.

Reviewer:2. As reconstruction of virus genome seems the main feature of TRACESPipe tool, this feature should be evaluated more thoroughly. For synthetic datasets, the authors showed if the tool can detect the presence of the virus and the breadth and depth coverage of the reconstructed genome in Table 2. Besides this, in order to ensure the quality of the reconstructed genome, the authors should compare the genome length of the reconstructed genome with the original one. This length comparison will show the percentage of the genome recovered by the tool. To check the quality of the reconstructed genome, the identity of the recovered genome with the original one should be reported. The identity can be computed by several ways,i.e., the average nucleotide identity can be computed by Mummer ”dnadiff” program, or average nucleotide identity can be plotted by Mummerplot, or a similarity plot can be generated by Blast. Similarly, for real data, the assembled genomes should be evaluated more

thoroughly. At least for the three reconstructed genomes reported in the paper (B19V, JCPyV, and human mitogenome), the length of assembled genome should be compared with the original one. Also, identity of the newly constructed genomes with the original one should be reported.

Authors: We thank the Reviewer for this suggestion. We have now included the dnadiff program from MUMmer4 as an automatic tool to measure the genome identity (including the percentage of aligned bases and the number of SNPs) between the reference and the reconstructed genome. The same approach was also included for the mitogenome reconstruction. Moreover, as an auxiliary tool, we included blastn as a local and remote feature. As for the quality measures, TRACESPipe can now automatically compute the breadth and depth coverage, genome identity, percentage of aligned bases, number of SNPs, and genome similarity. We implemented these new features on the analysis of the synthetic data (Table 2) and showed that TRACESPipe is able to reconstruct the genomes with very high quality, even when high mutation rates and low coverage were simulated. Correspondingly, we also included the comparisons for the real reconstructed genomes of B19V, JCPyV and the human mitogenome. These results are now presented in Figure 4 (b,c)and in the Supplementary Figure 5.3

Reviewer:3. As multiple instances of the same virus can be assembled from different organs, clarify how they are going to be evaluated. For example, for real data, JCPyV virus was reconstructed from both kidney and liver data. Give explanation on which instance of the assembled genome was picked up, what was the criteria of identifying an assembly as a better one, was this process automatic or human intervention was needed. If human intervention was needed, then give more explanation on how you had chosen a better assembly for the JCPyV virus so that the future users of the tool will be aware of the process.

Authors: There are two levels of interaction with the data: The first level is used to automatically choose the best reference, either for each organ, or to all organs (by calculating a best of bests). The latter is critical for direct comparison of the alignments derived from multiple organs. The second level creates a consensus from the merged reconstructed genomes. We have now included Figures 5 and 6, and Supplementary Figure2 to show the consensus and other characteristics (alignments, SNPs, genome structure, and complexity pro-files) depicting how the combination. The whole experiment is run automatically using a single command, provided in the Supplementary Section Reproducibility (Data analysis).

Reviewer:4. For real data, from figure 3 we can see that a number of viruses were present in the data. But, only three reconstructed genomes were reported (B19V, JCPyV, and human mitogenome). Include the assembly result for other viruses also, i.e., how much of those viruses were recovered by TRACESPipe tool.

Authors: TRACESPipe was designed and tailored-made for the analysis of within-host variability of viral sequences derived from multiple organs. In fact, we are currently evaluating soft and hard tissues from recently deceased individuals using this tool. The three reconstructed genomes are reported as examples to the pipeline description.

Reviewer: Minor concerns: 1. For synthetic datasets, mention total number of datasets.

Authors:This is now stated in the manuscript. We thank the Reviewer for the observation

Reviewer:2. For both synthetic and real data, provide a bit more details of applying different steps of TRACESPipe tool. Describe outcomes of applying different modules (compression-based prediction, sequence alignment, de novo assembly) of the tool to the synthetic and read datasets. Also describe outcomes of applying different controls (redundancy control, database control, exogenous control) of the tool to those datasets.

Authors: We thank the Reviewer for the suggestion. This is now included in the text and supplementary material.

Reviewer:3. In ”Real Data” section, in 2nd paragraph, ”an identity of 99%”, here specify what type of identity it is, average nucleotide identity or amino acid identity. Also, specify how this identity was calculated.

Authors:We refer to average nucleotide identity. This is now specified in the text.

References:
1. Bolduc B, Youens-Clark K, Roux S, Hurwitz BL, Sullivan MB. iVirus: facilitating new insights in viralecology with software and community data sets imbedded in a cyberinfrastructure. The ISME journal. 2017Jan;11(1):7-14.
2. Ajami NJ, Wong MC, Ross MC, Lloyd RE, Petrosino JF. Maximal viral information recovery fromsequence data using VirMAP. Nature communications. 2018 Aug 10;9(1):1-9.

Close
