# Supplementary material for: A hybrid pipeline for reconstruction and analysis of viral genomes at multi-organ level
Source: Gigascience. 2020 Aug 20;9(8):giaa086. doi: 10.1093/gigascience/giaa086 (PMC7439602; doi:10.1093/gigascience/giaa086)
Supplement: giaa086_Response_to_Reviewer_Comments_Revision_1 [file giaa086_response_to_reviewer_comments_revision_1.pdf]

Author's Response To Reviewer Comments

Close

Dear Editor,

Thank you for accepting our article.
All the changes have been made.

Best regards,
Diogo

Close
