# Supplementary material for: A hybrid pipeline for reconstruction and analysis of viral genomes at multi-organ level
Source: Gigascience. 2020 Aug 20;9(8):giaa086. doi: 10.1093/gigascience/giaa086 (PMC7439602; doi:10.1093/gigascience/giaa086)
Supplement: giaa086_Response_to_Reviewer_Comments_Revision_2 [file giaa086_response_to_reviewer_comments_revision_2.pdf]

Author's Response To Reviewer Comments

Close

Dear Hans,

Thank you!
The tools have been registered.
Thank you for spotting the typo in the data availability section.
The materials have been submitted to the editorialmanager.

Best wishes,
Diogo

Close
