# Supplementary material for: A hybrid pipeline for reconstruction and analysis of viral genomes at multi-organ level
Source: Gigascience. 2020 Aug 20;9(8):giaa086. doi: 10.1093/gigascience/giaa086 (PMC7439602; doi:10.1093/gigascience/giaa086)
Supplement: giaa086_Reviewer_1_Report_Original_Submission — Brett E. Pickett, Ph.D. -- 2/13/2020 Reviewed [file giaa086_reviewer_1_report_original_submission.pdf]

Reviewer Report

Title: A hybrid pipeline for reconstruction and analysis of viral genomes at multi-organ level

Version: Original Submission **Date:** 2/13/2020

Reviewer name: Brett E. Pickett, Ph.D.

Reviewer Comments to Author:

The manuscript entitled "A hybrid pipeline for reconstruction and analysis of viral genomes at multi-organ level ", by Pratas et al. describes their development of a novel bioinformatics pipeline named TRACESPipe. Overall this software specializes in assembling and analyzing viral genomes from multiple organ sites. As such, it will enable the rapid analysis of various types of datasets that explore intra-host viral diversity. The workflow is very logical and expresses an impressive knowledge of the tools involved as well as the file formats that are produced.

The described methods are appropriate for this study and the authors do an excellent job to ensure that multiple approaches are used to facilitate performing the necessary controls on the data being analyzed.

The authors do a good job with ensuring that the conclusions drawn are supported by the data and results reported elsewhere in the manuscript.

The manuscript could be improved by including additional text to describe:

1) Why 40 was selected as the optimal number of genes with high similarity scores? Can the authors provide additional justification and/or data to reinforce this decision?

2) The abbreviations used in the caption for Table 2. Although these abbreviations are defined in the List of Abbreviations at the end of the manuscript, readability could be improved by including the relevant abbreviations in the Table caption.

Although not required for the current publication, it may be helpful for the authors to consider the below recommendations as part of future development plans:

1) Enable the analysis of mitochondrial sequence from non-human primates. These animals are often used as model organisms for a variety of viruses. As such, the userbase of the TRACESPipe software could be expanded by providing this capability.

2) Adapt the installation process such that TRACESPipe can be installed into an auto-generated Conda environment (i.e. "conda install -c bioconda TRACESPipe")?

Level of Interest

Please indicate how interesting you found the manuscript: Choose an item.

Quality of Written English

Please indicate the quality of language in the manuscript: Choose an item.

Declaration of Competing Interests

Please complete a declaration of competing interests, considering the following questions:

- Have you in the past five years received reimbursements, fees, funding, or salary from an organisation that may in any way gain or lose financially from the publication of this manuscript, either now or in the future?
- Do you hold any stocks or shares in an organisation that may in any way gain or lose financially from the publication of this manuscript, either now or in the future?
- Do you hold or are you currently applying for any patents relating to the content of the manuscript?
- Have you received reimbursements, fees, funding, or salary from an organization that holds or has applied for patents relating to the content of the manuscript?
- Do you have any other financial competing interests?
- Do you have any non-financial competing interests in relation to this paper?

If you can answer no to all of the above, write 'I declare that I have no competing interests' below. If your reply is yes to any, please give details below.

I declare that I have no competing interests.

I agree to the open peer review policy of the journal. I understand that my name will be included on my report to the authors and, if the manuscript is accepted for publication, my named report including any attachments I upload will be posted on the website along with the authors' responses. I agree for my report to be made available under an Open Access Creative Commons CC-BY license (http://creativecommons.org/licenses/by/4.0/). I understand that any comments which I do not wish to be included in my named report can be included as confidential comments to the editors, which will not be published.

Choose an item.

To further support our reviewers, we have joined with Publons, where you can gain additional credit to further highlight your hard work (see: https://publons.com/journal/530/gigascience). On publication of this paper, your review will be automatically added to Publons, you can then choose whether or not to claim your Publons credit. I understand this statement.

Yes Choose an item.
