# Supplementary material for: A hybrid pipeline for reconstruction and analysis of viral genomes at multi-organ level
Source: Gigascience. 2020 Aug 20;9(8):giaa086. doi: 10.1093/gigascience/giaa086 (PMC7439602; doi:10.1093/gigascience/giaa086)
Supplement: giaa086_Reviewer_2_Report_Original_Submission — Saima Sultana Tithi, Ph.D. -- 2/22/2020 Reviewed [file giaa086_reviewer_2_report_original_submission.pdf]

Reviewer Report

Title: A hybrid pipeline for reconstruction and analysis of viral genomes at multi-organ level

Version: Original Submission **Date:** 2/22/2020

Reviewer name: Saima Sultana Tithi, Ph.D.

Reviewer Comments to Author:

In this manuscript, the authors present a new pipeline for reconstruction of virus genomes from multiple organs simultaneously. This will be a useful pipeline for analyzing virus data from processing raw reads to downstream analysis as this tool can start working from the raw read data, can do both the alignment of the read and then assembly of the virus genome as well as report the variants found in the reconstructed genomes which will be helpful for the downstream analysis.

Major concerns:

1. As TRACESPipe is a computational pipeline for analyzing virus data, the features of TRACESPipe should be compared with other existing pipelines, i.e., iVirus [1], VirMap [2] to highlight the novel features of TRACESPipe tool and to highlight the difference of this tool from other existing tools.
2. As reconstruction of virus genome seems the main feature of TRACESPipe tool, this feature should be evaluated more thoroughly. For synthetic datasets, the authors showed if the tool can detect the presence of the virus and the breadth and depth coverage of the reconstructed genome in Table 2. Besides this, in order to ensure the quality of the reconstructed genome, the authors should compare the genome length of the reconstructed genome with the original one. This length comparison will show the percentage of the genome recovered by the tool. To check the quality of the reconstructed genome, the identity of the recovered genome with the original one should be reported. The identity can be computed by several ways, i.e., the average nucleotide identity can be computed by Mummer "dnadiff" program, or average nucleotide identity can be plotted by Mummerplot, or a similarity plot can be generated by Blast.
Similarly, for real data, the assembled genomes should be evaluated more thoroughly. At least for the three reconstructed genomes reported in the paper (B19V, JCPyV, and human mitogenome), the length of assembled genome should be compared with the original one. Also, identity of the newly constructed genomes with the original one should be reported.
3. As multiple instances of the same virus can be assembled from different organs, clarify how they are going to be evaluated. For example, for real data, JCPyV virus was reconstructed from both kidney and liver data. Give explanation on which instance of the assembled genome was picked up, what was the criteria of identifying an assembly as a better one, was this process automatic or human intervention was needed. If human intervention was needed, then give more explanation on how you had chosen a better assembly for the JCPyV virus so that the future users of the tool will be aware of the process.
4. For real data, from figure 3 we can see that a number of viruses were present in the data. But, only three reconstructed genomes were reported (B19V, JCPyV, and human mitogenome). Include the assembly result for other viruses also, i.e., how much of those viruses were recovered by TRACESPipe tool.

Minor concerns:
1. For synthetic datasets, mention total number of datasets.
2. For both synthetic and real data, provide a bit more details of applying different steps of TRACESPipe tool. Describe outcomes of applying different modules (compression-based prediction, sequence alignment, de novo assembly) of the tool to the synthetic and read datasets. Also describe outcomes of applying different controls (redundancy control, database control, exogenous control) of the tool to those datasets.
3. In "Real Data" section, in 2nd paragraph, "an identity of 99%", here specify what type of identity it is, average nucleotide identity or amino acid identity. Also, specify how this identity was calculated.
[1] Bolduc B, Youens-Clark K, Roux S, Hurwitz BL, Sullivan MB. iVirus: facilitating new insights in viral ecology with software and community data sets imbedded in a cyberinfrastructure. The ISME journal. 2017 Jan;11(1):7-14.
[2] Ajami NJ, Wong MC, Ross MC, Lloyd RE, Petrosino JF. Maximal viral information recovery from sequence data using VirMAP. Nature communications. 2018 Aug 10;9(1):1-9.

Level of Interest

Please indicate how interesting you found the manuscript: Choose an item.

Quality of Written English

Please indicate the quality of language in the manuscript: Choose an item.

Declaration of Competing Interests

Please complete a declaration of competing interests, considering the following questions:

- Have you in the past five years received reimbursements, fees, funding, or salary from an organisation that may in any way gain or lose financially from the publication of this manuscript, either now or in the future?
- Do you hold any stocks or shares in an organisation that may in any way gain or lose financially from the publication of this manuscript, either now or in the future?
- Do you hold or are you currently applying for any patents relating to the content of the manuscript?
- Have you received reimbursements, fees, funding, or salary from an organization that holds or has applied for patents relating to the content of the manuscript?
- Do you have any other financial competing interests?
- Do you have any non-financial competing interests in relation to this paper?

If you can answer no to all of the above, write 'I declare that I have no competing interests' below. If your reply is yes to any, please give details below.

I declare that I have no competing interests.

I agree to the open peer review policy of the journal. I understand that my name will be included on my report to the authors and, if the manuscript is accepted for publication, my named report including any attachments I upload will be posted on the website along with the authors' responses. I agree for my report to be made available under an Open Access Creative Commons CC-BY license (http://creativecommons.org/licenses/by/4.0/). I understand that any comments which I do not wish to be included in my named report can be included as confidential comments to the editors, which will not be published.

Choose an item.

To further support our reviewers, we have joined with Publons, where you can gain additional credit to further highlight your hard work (see: https://publons.com/journal/530/gigascience). On publication of this paper, your review will be automatically added to Publons, you can then choose whether or not to claim your Publons credit. I understand this statement.

Yes Choose an item.
